# Comparing neoantigen cancer vaccines and immune checkpoint therapy unveils an effective vaccine and anti-TREM2 macrophage-targeting dual therapy

**DOI:** 10.1016/j.celrep.2024.114875

**Published:** 2024-10-23

**Authors:** Sunita Keshari, Alexander S. Shavkunov, Qi Miao, Akata Saha, Tomoyuki Minowa, Martina Molgora, Charmelle D. Williams, Mehdi Chaib, Anna M. Highsmith, Josué E. Pineda, Sayan Alekseev, Elise Alspach, Kenneth H. Hu, Marco Colonna, Kristen E. Pauken, Ken Chen, Matthew M. Gubin

**Affiliations:** 1 Department of Immunology, The University of Texas MD Anderson Cancer Center, Houston, TX, USA; 2 Department of Bioinformatics and Computational Biology, The University of Texas MD Anderson Cancer Center, Houston, TX, USA; 3 Department of Pathology and Immunology, Washington University School of Medicine in Saint Louis, St. Louis, MO, USA; 4 Program of Biology, The University of Texas at San Antonio, San Antonio, TX, USA; 5 Department of Molecular Microbiology and Immunology, Saint Louis University School of Medicine, St. Louis, MO, USA; 6 The Parker Institute for Cancer Immunotherapy, The University of Texas MD Anderson Cancer Center, Houston, TX, USA; 7 The James P. Allison Institute, The University of Texas MD Anderson Cancer Center, Houston, TX, USA; 8 Present address: Department of Immunology, H. Lee Moffitt Cancer Center & Research Institute, Tampa, FL, USA; 9 Lead contact

## Abstract

The goal of therapeutic cancer vaccines and immune checkpoint therapy (ICT) is to promote T cells with antitumor capabilities. Here, we compared mutant neoantigen (neoAg) peptide-based vaccines with ICT in preclinical models. NeoAg vaccines induce the most robust expansion of proliferating and stem-like PD-1^+^TCF-1^+^ neoAg-specific CD8 T cells in tumors. Anti-CTLA-4 and/or anti-PD-1 ICT promotes intratumoral TCF-1^−^ neoAg-specific CD8 T cells, although their phenotype depends in part on the specific ICT used. Anti-CTLA-4 also prompts substantial changes to CD4 T cells, including induction of ICOS^+^Bhlhe40^+^ T helper 1 (Th1)-like cells. Although neoAg vaccines or ICTs expand iNOS^+^ macrophages, neoAg vaccines maintain CX3CR1^+^CD206^+^ macrophages expressing the TREM2 receptor, unlike ICT, which suppresses them. TREM2 blockade enhances neoAg vaccine efficacy and is associated with fewer CX3CR1^+^CD206^+^ macrophages and induction of neoAg-specific CD8 T cells. Our findings highlight different mechanisms underlying neoAg vaccines and different forms of ICT and identify combinatorial therapies to enhance neoAg vaccine efficacy.

## In brief

Keshari et al. demonstrate in preclinical models that neoantigen peptide-based vaccines, anti-PD-1, and anti-CTLA-4 each induce partially distinct tumor microenvironment remodeling. Targeting intratumoral macrophages via TREM2 blockade extends the therapeutic window of neoantigen vaccines and is associated with a decrease in CX3CR1^+^CD206^+^ macrophages and the promotion of neoantigen-specific T cells.

## Graphical Abstract

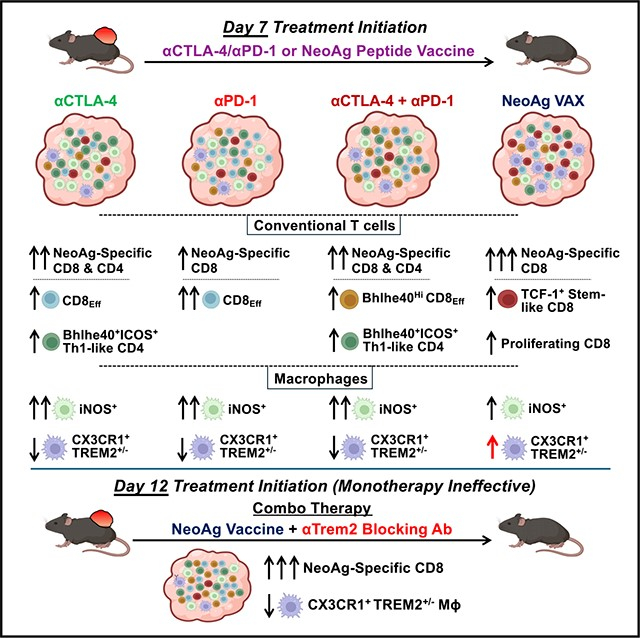

## INTRODUCTION

For many cancer immunotherapies, T cell recognition of tumor antigens is critical for efficacy.^1–4^ Tumor-specific neoantigens (neoAgs) formed from somatic alterations are largely omitted from immune tolerance and are exclusively expressed in cancer cells, making them favorable cancer vaccine targets.^1,3,4^ NeoAg vaccines have shown promise in early-phase clinical trials,^5–14^ but many fundamental questions regarding neoAg vaccines remain unclear,^15^ including how to best combine vaccines with other therapeutic modalities.

We previously used immunogenomic/mass spectrometry approaches to identify neoAgs and demonstrated that neoAg cancer vaccines could provoke tumor rejection in methylcholanthrene (MCA)-induced sarcoma models.^16^ Others used similar approaches to identify neoAgs.^3,6,7,17–20^ We further showed that neoAgs are major targets of T cells reactivated by immune checkpoint therapy (ICT) and that anti-CTLA-4 and anti-PD-1 ICT induces changes in both CD4 and CD8 T cells within the tumor microenvironment (TME),^16,21–24^ consistent with other studies.^25,26^ While both conventional CD4 and CD8 T cells drive immunotherapeutic responses to cancer,^16,23,27–29^ CD8 T cells are often the most potent direct inducers of tumor cell death.^30^ Intratumoral CD8 T cells expressing activation markers, including inhibitory receptors like PD-1, LAG-3, and TIM-3, often exist in a terminally differentiated state ranging from short-lived cytotoxic effectors to dysfunctional/exhausted states.^30–33^ The latter are characterized by reduced function, sustained inhibitory receptor expression, and unique transcriptional and epigenetic profiles, distinguishing them from memory and stem-like T cells (progenitor/precursor exhausted [Pex] CD8 T cells).^30,32,33^ Whereas the transcription factor TCF-1 supports stemness or memory-like attributes^34,35^ in Pex/Pex-like CD8 T cells, chronic antigen exposure and/or signals within the TME promote TOX expression, leading to a fixed epigenetic landscape in terminally dysfunctional/exhausted CD8 T (Tex) cells.^33,36–40^ PD-1^hi^TOX^+^TCF-1^−^ Tex cells likely lack the ability to recover potent effector function following immunotherapies such as PD-1/PD-L1 blockade.^30,31,41,42^ Instead, PD-1^+^TCF-1^+^ Pex/Pex-like CD8 T cells within tumors/lymph nodes expand and differentiate into PD-1^+^TCF-1^−^ CD8 T effector-like cells in response to anti-PD-1/PD-L1.^30–32,43–45^

While tumor lymphoid and myeloid immune compositions play a major role in response to immunotherapy,^24–26,46–51^ the heterogeneity and dynamics of immune infiltrates in response to immunotherapies such as ICT and neoAg cancer vaccines are not thoroughly characterized. We developed preclinical models to interrogate potential synergies between the mechanisms underlying neoAg synthetic long peptide (SLP) cancer vaccines and different ICTs. NeoAg SLP vaccines induced the most robust expansion of polyfunctional neoAg-specific CD8 T cells, including proliferating and stem-like/Pex-like CD8 T cells. Anti-CTLA-4 and/or anti-PD-1 ICT increased the frequency and effector function of intratumoral neoAg-specific CD8 T cells, with anti-CTLA-4-containing treatments also dramatically altering CD4 T cells. Both neoAg SLP vaccines and ICT promoted expansion of intratumoral M1-like inducible nitric oxide synthase (iNOS)^+^ macrophages, and while ICT reduced the frequency of M2-like CX3CR1^+^CD206^+^ macrophages, including those expressing the TREM2 receptor, CX3CR1^+^CD206^+^ macrophages were largely maintained in neoAg SLP vaccine-treated mice. NeoAg SLP vaccination in combination with either antiCTLA-4 or anti-PD-1 displayed enhanced efficacy, further supporting the rationale of combining neoAg SLP vaccines with ICT. Finally, based on our observations in the macrophage compartment upon neoAg SLP vaccination, we assessed a combination of neoAg SLP vaccination and anti-TREM2, demonstrating enhanced efficacy of this combination associated with a decrease in CX3CR1^+^CD206^+^ macrophages and promotion of interferon-γ (IFN-γ)^+^ neoAg-specific CD8 T cells.

## RESULTS

### NeoAg SLP vaccines and ICT induce T cell-dependent long-term tumor protection

**Figure 1.**
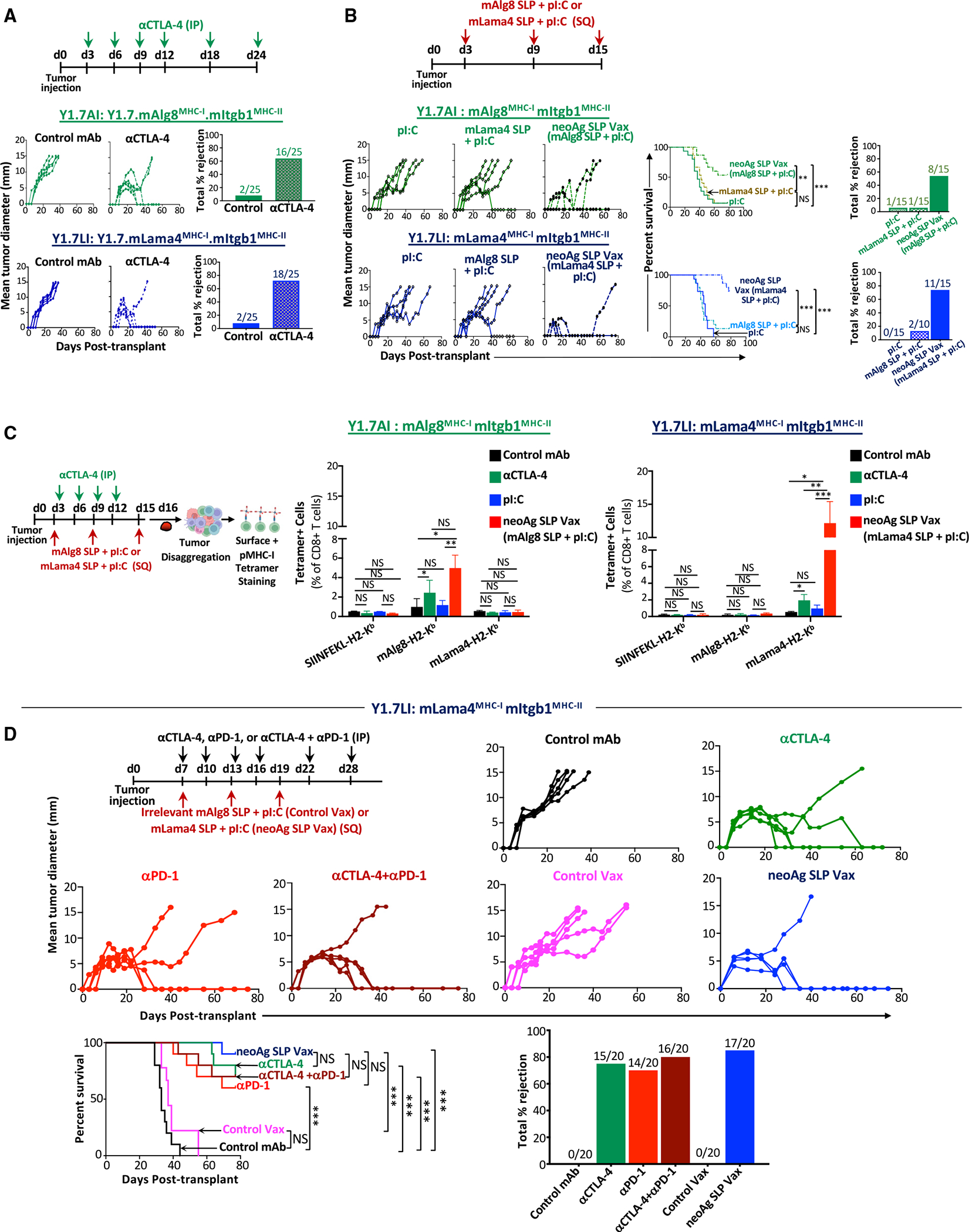
NeoAg SLP vaccines or ICTs inhibit neoAg-expressing *Braf*^*V600E*^*Pten*^−/−^*Cdkn2a*^−/−^ melanoma outgrowth (A) Tumor growth and percentage tumor rejection in mice transplanted with Y1.7mA^MHC-I^.mI^MHC-II^ (Y1.7AI) or Y1.7mL^MHC-I^.mI^MHC-II^ (Y1.7LI) melanoma cells and treated with control mAb or anti-CTLA-4 ICT starting on day 3 post-tumor transplant. (B) Tumor growth, cumulative mouse survival, and percentage tumor rejection in Y1.7AI and Y1.7LI melanoma-bearing mice treated with mAlg8 or mLama4 neoAg SLP (plus pI:C) vaccines or pI:C alone starting on day 3 post-tumor transplant. (C) mAlg8 or mLama4 tetramer-specific CD8 T cells in Y1.7AI and Y1.7LI tumors treated with control mAb, anti-CTLA-4, pI:C, mAlg8 SLP + pI:C (neoAg SLP vaccine for Y1.7AI), or mLama4 SLP + pI:C (neoAg SLP vaccine for Y1.7LI) as in (A) and (B) and harvested on day 16 post-tumor transplant. SIINFEKL-H2-K^b^ tetramer served as an irrelevant control. (D) Tumor growth, cumulative mouse survival, and percentage tumor rejection in Y1.7LI tumor-bearing mice treated with control mAb, anti-CTLA-4, anti-PD-1, anti-CTLA-4 + anti-PD-1, irrelevant (for Y1.7LI) mAlg8 SLP + pI:C (control vax), or relevant mLama4 SLP + pI:C (neoAg SLP vax) starting on day 7 post-tumor transplant. For (A), (B), and (D), tumor growth is presented as mean tumor diameter of individual mice, tumor rejection graphs display cumulative percentage of mice with complete tumor rejection, and cumulative survival curves include mice from at least three independent experiments (**p < 0.01, ***p < 0.001; NS, not significant; log-rank [Mantel-Cox] test). Bar graphs in (C) display mean ± SEM and are representative of at least three independent experiments (*p < 0.05, **p < 0.01, ***p < 0.005; NS, not significant; unpaired, two-tailed Student’s t test). See also Figure S1.

For this study, we modified the genetically engineered mouse model (GEMM)-derived *Braf*^*V600E*^*Pten*^−/−^*Cdkn2a*^−/−^ YUMM1.7 melanoma line^52^ to express different combinations of major histocompatibility complex (MHC) class I (MHC-I) and MHC-II neoAgs. GEMM tumors are generally poorly immunogenic; however, they can be engineered to express neoAgs to study tumor-immune system interactions.^23,53–56^ We engineered YUMM1.7 to express known tumor neoAgs via introduction of minigenes encoding the G1254V mutation in Lama4 (mLama4^MHC-I^), the A506T mutation in Alg8 (mAlg8^MHC-I^), and the N710Y mutation in Itgb1 (mItgb1^MHC-II^) neoAgs^16,23^ in various combinations: mLama4^MHC-I^ + mItgb1^MHC-II^ (Y1.7LI line) or mAlg8^MHC-I^ + mItgb1^MHC-II^ (Y1.7AI line) (Figure S1A). Consistent with prior observations,^57^ the parental YUMM1.7 melanoma line was insensitive to anti-CTLA-4 and/or anti-PD-1 ICT (Figure S1B). In contrast, expression of mLama4^MHC-I^ or mAlg8^MHC-I^ neoAg along with mItgb1^MHC-II^ neoAg rendered YUMM1.7 melanoma lines (Y1.7LI and Y1.7AI) sensitive to anti-CTLA-4 ICT (Figure 1A).

We next asked whether therapeutic cancer vaccines composed of the SLP containing the minimal MHC-I neoAg epitope^16^ and the adjuvant poly(I:C) (pI:C) could induce regression of the Y1.7LI and Y1.7AI neoAg-expressing lines. Tumor-bearing mice treated with pI:C alone displayed outgrowth of Y1.7LI or Y1.7AI melanoma, whereas vaccines comprising relevant neoAg SLP + pI:C (neoAg SLP vax) induced rejection or delayed outgrowth of Y1.7LI or Y1.7AI (Figure 1B). NeoAg vaccine-induced tumor rejection was dependent upon specific neoAg expression, as mAlg8 SLP + pI:C did not induce Y1.7LI (mLama4 expressing) tumor rejection and vice versa with Y1.7AI (mAlg8 expressing) (Figure 1B). Mice that rejected Y1.7AI or Y1.7LI tumors upon neoAg SLP vax or anti-CTLA-4 were rechallenged in the absence of additional treatment with the same tumor lines 60+ days after rejection of the primary tumors. Upon secondary challenge, no detectable tumor was observed, indicating long-term protection against rechallenge with the same tumor (Figure S1C). In contrast, both Y1.7-neoAg-expressing lines grew out when injected into naive mice in the absence of treatment. When mice that previously rejected Y1.7LI tumors were rechallenged with parental YUMM1.7, progressive tumor growth was observed (Figure S1D), indicating that immunity was neoAg specific.

Y1.7AI or Y1.7LI tumors from anti-CTLA-4-treated mice contained greater frequencies of mAlg8- or mLama4-specific CD8 T cells compared to mice receiving control monoclonal antibody (mAb) (Figures 1C and S1E). Whereas pI:C alone had little effect on the frequency of neoAg-specific CD8 T cells, neoAg SLP vax induced a 4-fold or more increase in mAlg8- or mLama4-specific CD8 T cells (Figures 1C and S1E), including those co-expressing the inhibitory receptors PD-1 and TIM-3 (Figure S1F); however, this itself does not necessarily indicate reduced function.^30,58^

Next, we focused on Y1.7LI and delayed treatment initiation until day 7 post-transplant, a time point at which anti-CTLA-4, anti-PD-1, anti-CTLA-4 plus anti-PD-1, or neoAg SLP vax still induced tumor rejection in a majority of mice (Figure 1D). ICT- and neoAg SLP vax-induced tumor rejection was dependent on both CD4 and CD8 T cells, since depleting either subset abolished therapeutic efficacy (Figure S2A). Y1.7LI-rechallenged mice that rejected Y1.7LI upon neoAg SLP vax or anti-CTLA-4 and/or anti-PD-1 initiated on day 7, but not untreated naive mice, showed no detectable tumor upon secondary challenge (Figure S2B).

### TME remodeling induced by neoAg SLP vaccines and ICT

**Figure 2.**
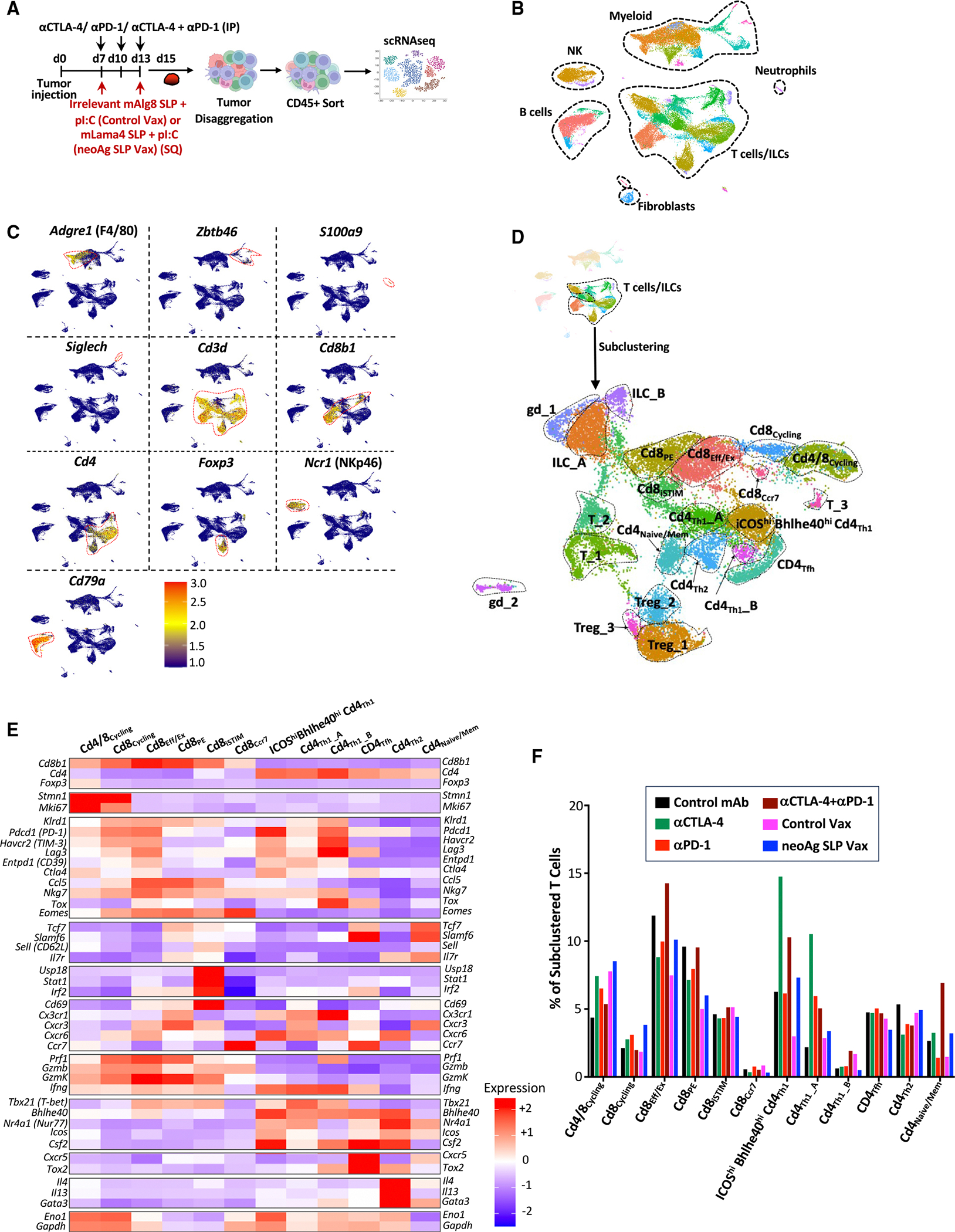
scRNA-seq of intratumoral immune cells from Y1.7LI melanoma-bearing mice treated with neoAg SLP vaccines or ICT (A) Y1.7LI melanoma-bearing mice were treated as indicated beginning on day 7 post-tumor transplant. Tumors from individual mice were harvested on day 15, pooled, and processed, and live CD45^+^ cells were sorted and analyzed by scRNA-seq. (B) Uniform manifold approximation and projection (UMAP) plot from scRNA-seq of intratumoral CD45^+^ cells with annotated cell types. (C) Feature plot showing lineage-specific transcripts. (D) Feature plots displaying subclustering of activated T cell-containing clusters. (E) Heatmap displaying average expression of select transcripts by cluster. (F) Frequency of subclustered T cell-containing clusters by treatment. See also Figure S4 and Table S1.

We next used an unbiased approach to assess whether effective neoAg SLP vaccines induced TME alterations that are distinct from or overlapping with different forms of ICT. Y1.7LI tumor-bearing mice were treated with control mAb, anti-CTLA-4 and/or anti-PD-1, control vax, or neoAg SLP vax beginning on day 7 (Figure 2A). Tumors were harvested on day 15 (a critical time point prior to tumor rejection), and CD45^+^ cells were sorted for single-cell RNA sequencing (scRNA-seq), revealing clusters of myeloid cells and lymphocytes (Figures 2B and 2C). scRNA-seq and flow cytometry both indicated that immunotherapy altered the proportions of different myeloid and lymphoid subsets (Figure S3A).

To gain more insights into how the different immunotherapies altered intratumoral T cells, clusters containing activated T cells were subclustered, yielding multiple subsets of conventional T cells, Foxp3^+^CD4^+^ T regulatory cells (Tregs), gamma delta T cells (gdT), and innate lymphoid cells (ILCs) (Figures 2D, 2E, S3B–S3E, and S4A; Table S1). Cluster Cd4/8_Cycling_ contained a mix of Tregs, CD4 T cells, and CD8 T cells and displayed a cell proliferation/cycling transcriptional signature (Figures 2D–2F, S4A, S4B; Table S1). Anti-CTLA-4-, anti-PD-1-, control vax-, or neoAg SLP vax-treated mice contained a greater frequency of cells within Cd4/8_Cycling_ compared to control mAb (Figure S4C). Anti-CTLA-4 (±anti-PD-1) reduced proliferating Tregs and expanded CD4 T cells within Cd4/8_Cycling_, while the ratio of proliferating CD8 T cells to Tregs or CD4 T cells was higher with anti-PD-1 (Figures S4D–S4F). NeoAg SLP vax contained the greatest ratio of cycling CD8 T cells to other T cells in this cluster compared to all other conditions (Figure S4E).

We identified five exclusively CD8 T cell clusters, spanning a range of activation states, including proliferating (Cd8_Cycling_), CD69^hi^ IFN-stimulated (Cd8_iSTIM_), PD-1^+^TCF-1^+^ stem-like/Pex-like (Cd8_PE_), and PD-1^+^TCF-1^−^ terminal effectors or dysfunctional/exhausted CD8 T cells (Cd8_Eff/Ex_) (Figures 2D, 2E, S4A, and S5A–S5C; Table S1).

### Anti-PD-1 expands neoAg-specific CD8 effector T cells, including those with high Bhlhe40 expression, particularly when combined with anti-CTLA-4

**Figure 3.**
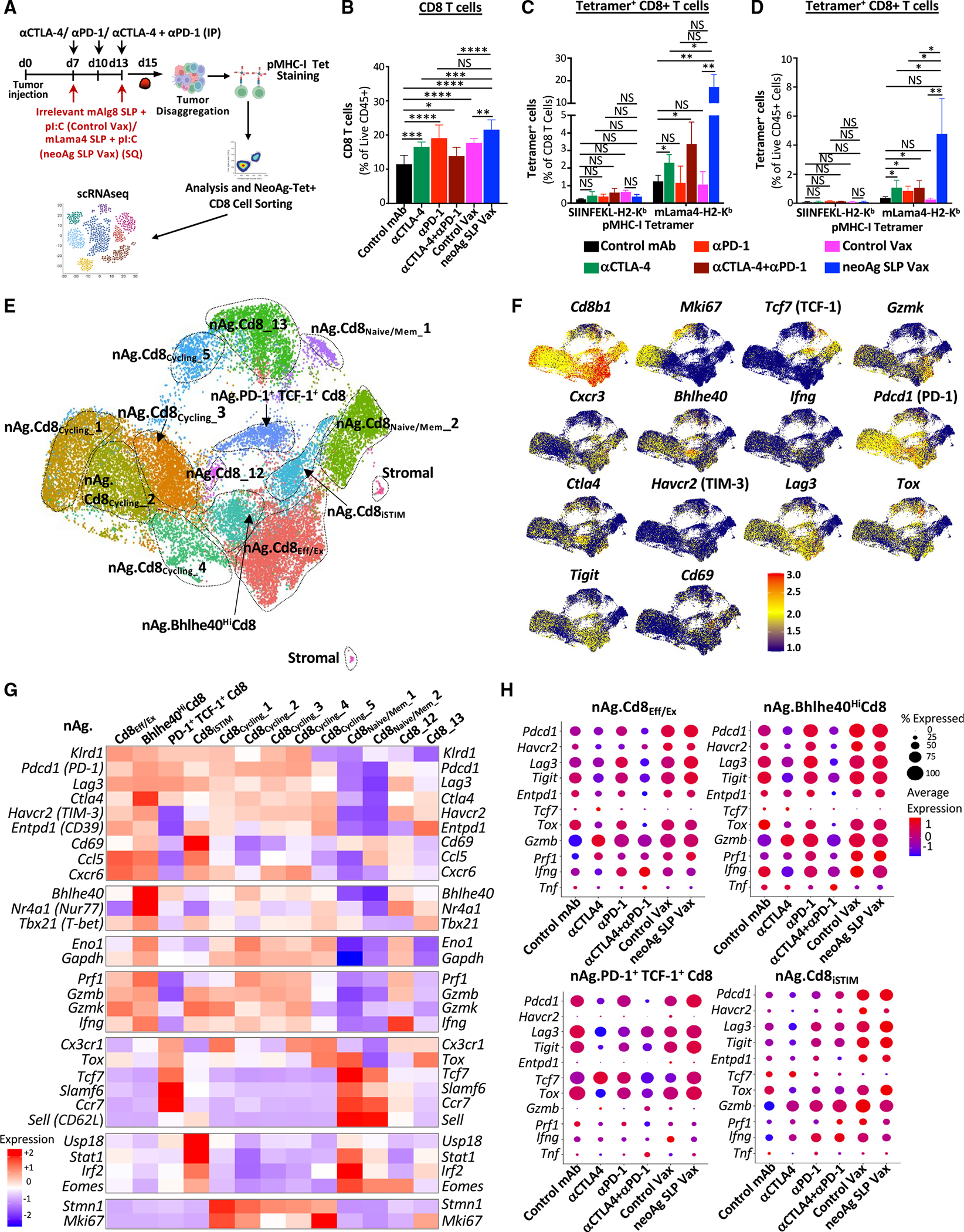
Characterization of neoAg-specific CD8 T cells in neoAg SLP vaccine- and ICT-treated mice (A) Y1.7LI melanoma-bearing mice were treated as indicated beginning on day 7 post-tumor transplant. Tumors from individual mice were harvested on day 15, pooled, processed, and stained with mLama4-H2-K^b^ tetramers for analysis (B–D) or for sorting of mLama4 tetramer-positive CD8 T cells for scRNA-seq (E–H). (B) Graph displaying CD8 T cells as a percentage of intratumoral live CD45^+^ cells. (C and D) Graphs displaying irrelevant SIINFELK tetramer- or mLama4 tetramer-positive CD8 T cells as a percentage of (C) CD8 T cells and (D) CD45^+^ cells. (E) UMAP plot and cell-type annotations from scRNA-seq of mLama4 neoAg-specific CD8 T cells. (F) Feature plots displaying expression of select phenotype and lineage transcripts. (G) Heatmap displaying average expression of select transcripts by cluster. (H) scRNA-seq dot plot depicting select transcripts within select mLama4 neoAg-specific CD8 T cell clusters by treatment. Bar graphs in (B), (C), and (D) display the mean ± SEM and are representative of at least three independent experiments (*p < 0.05, **p < 0.01, ***p < 0.005, ****p < 0.0001; NS, not significant, unpaired t test). See also Figures S6A and S6B and Table S1.

We and others previously demonstrated that tumor-specific CD8 T cells have unique properties and that immunotherapy primarily affects tumor-reactive versus bystander CD8 T cells.^16,21,22,59–61^ Therefore, we analyzed mLama4 neoAg-specific CD8 T cells (Figure 3A). Anti-CTLA-4 and/or anti-PD-1 increased the overall frequency of intratumoral CD8 T cells (Figure 3B). Anti-CTLA-4 (±anti-PD-1) also drove a significant increase in mLama4-specific CD8 T cells as a percentage of CD8 T cells or CD45^+^ cells. Anti-PD-1 significantly increased mLama4-specific CD8 T cells as a percentage of CD45^+^ cells (Figures 3C, 3D, and S6A). NeoAg SLP vaccines drove the greatest increase in mLama4-specific CD8 T cells, from less than 2% (control mAb or control vax) to over 20% of CD8 T cells, which accounted for over 4% of intratumoral CD45^+^ cells (Figures 3C, 3D, and S6A).

**Figure 4.**
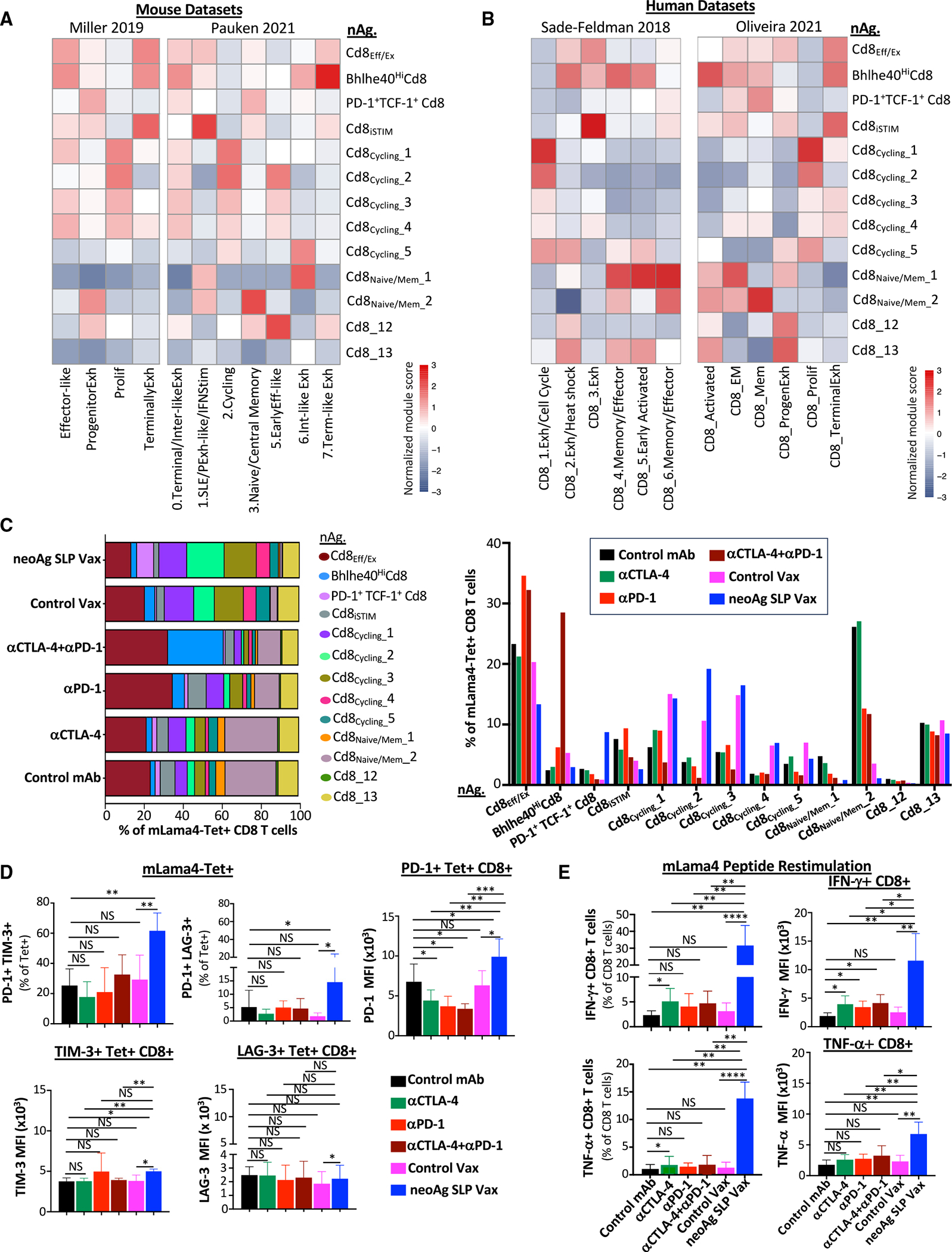
NeoAg SLP vaccines and ICT induce overlapping and distinct alterations to neoAg-specific CD8 T cells (A) Heatmaps comparing features (module scores) of mLama4 neoAg-specific CD8 T cell clusters (rows) to published mouse CD8 T cell gene signatures (columns) identified/annotated (e.g., “effector-like”) by Miller et al.^31^ and Pauken et al.^60^ (B) Heatmaps comparing features (module scores) of mLama4 neoAg-specific CD8 T cell clusters (rows) to published human CD8 T cell gene signatures (columns) identified/annotated (e.g., “CD8_1.Exh/Cell Cycle”) by Sade-Feldman et al.^41^ and Oliveira et al.^59^ (C) Frequency of mLama4 neoAg-specific CD8 T cells within each cluster by treatment depicted in two ways. (D) Graphs displaying percentage of PD-1^+^TIM-3^+^ or PD-1^+^LAG-3^+^ or PD-1, TIM-3, or LAG-3 mean fluorescence intensity (MFI) on PD-1^+^, TIM-3^+^, or LAG-3^+^ mLama4-specific CD8 T cells in Y1.7LI tumors. (E) Graph displaying IFN-γ^+^ or TNF-α^+^ CD8 T cells and IFN-γ or TNF-α MFI as assessed by ICS of mLama4 peptide-restimulated CD8 T cells isolated from Y1.7LI tumors. For (D) and (E), mice were treated beginning on day 7 post-tumor transplant and harvested on day 15. Bar graphs display the mean ± SEM and are representative of at least three independent experiments (*p < 0.05, **p < 0.01, ***p < 0.005, ****p < 0.0001; NS, not significant, unpaired t test). See also Figure S6C and Table S2.

Since scRNA-seq of CD45^+^ cells did not distinguish neoAg-specific CD8 T cells, we profiled sorted neoAg-specific CD8 T cells by scRNA-seq (Figure 3A). NeoAg-specific CD8 T cell clusters were annotated based on expression of select transcripts and comparisons with both mouse and human published datasets^31,41,59,60^ (Figures 3E–3H, 4A, 4B, S6B, and S7A; Table S1).

Clusters nAg.Cd8_Eff/Ex_ and nAg.Bhlhe40^Hi^Cd8 expressed *Pdcd1*, *Havcr2* (TIM-3), *Lag3*, and *Tigit*, as well as effector transcripts (e.g., *Nkg7*, *Ccl5*, *Gzmb*, *Gzmk*, *Prf1*, and *Cxcr6*). These two clusters also expressed *Tox* and exhibited little to no detectable expression of *Tcf7* (TCF-1) (Figures 3F–3H and S6B), consistent with activated effector or Tex CD8 T cells. Whereas the proportion of cells in nAg.Cd8_Eff/Ex_ increased with anti-PD-1 (±anti-CTLA-4), neoAg SLP vax reduced the proportion of nAg.Cd8_Eff/Ex_ cells compared to control mAb or control vax (Figure 4C). The top defining marker of cluster nAg.Bhlhe40- ^Hi^Cd8 was *Bhlhe40* (Figure 3G; Table S1), a transcription factor we previously demonstrated was upregulated in tumor-specific T cells and required for CD4 and/or CD8 T cell effector function and response to ICT.^24^ In addition to *Bhlhe40* (as well as *Pdcd1*, *Havcr2*, and *Lag3*), this cluster notably expressed other transcripts induced by T cell receptor (TCR) activation (e.g., *Ctla4*, *Cd69*, *Nr4a1*, and *Nr4a3*) and displayed high expression of *Tbx21* (T-bet) and *Ifng* (Figures 3G, 3H, and S6B). Compared to control mAb treatment, where nAg.Bhlhe40^Hi^Cd8 represented ~2.4% of mLama4-specific CD8 T cells, an ~2.6-fold increase occurred with anti-PD-1 (Figure 4C). Strikingly, anti-CTLA-4 and anti-PD-1 combination ICT increased this cluster to over 28% of mLama4-specific CD8 T cells.

In addition to increasing the frequency of cells within nAg. Cd8_Eff/Ex_ and nAg.Bhlhe40^Hi^Cd8, combination ICT increased the expression of *Bhlhe40*, *Fasl*, *Il7r*, *Icos*, and *Cd28* while decreasing *Tox*, *Pdcd1*, *Lag3*, *Entpd1*, and *Tigit* expression within both clusters (Figures 3H and S6B). The decrease in *Tox*, *Pdcd1*, *Lag3*, *Entpd1*, and *Tigit* was also observed with anti-CTLA-4 monotherapy (Figures 3H and S6B). In contrast, increased *Bhlhe40* expression was most prominent in the presence of anti-PD-1. Other features (e.g., increased *Icos*, *Cd28*, and *Fasl*) were unique to anti-CTLA-4 and anti-PD-1 combination ICT (Figure S6B).

### NeoAg SLP vaccination promotes PD-1^+^TCF-1^+^ stem-like/Pex-like and proliferating neoAg-specific CD8 T cells

NeoAg SLP vax drove an over 3- and 8-fold increase in the frequency of mLama4-specific CD8 T cells within cluster nAg.PD-1^+^TCF-1^+^Cd8 compared to control mAb and control vax, respectively (Figure 4C). Cluster nAg.PD-1^+^TCF-1^+^Cd8 displayed high expression of *Pdcd1*; low to moderate expression of *Ifng*, *Gzmk*, and *Prf1*; and little to no detectable expression of *Havcr2* or *Entpd1* (Figures 3G and 3H). nAg.PD-1^+^TCF-1^+^Cd8 also expressed *Ccr7*, *Bach2*, *Slamf6*, and *Tcf7* (TCF-1), indicative of plastic or stem-like features observed in Pex/Pex-like CD8 T cells (Figures 3G and 3H; Table S1). nAg.PD-1^+^TCF-1^+^Cd8 also expressed *Xcl1* (Table S1), encoding a chemoattractant for Xcr1^+^ type I conventional dendritic cells (cDC1s).^62^ While neoAg SLP vax promoted this population, the proportion of neoAg-specific CD8 T cells within this cluster was reduced with combination anti-CTLA-4 and anti-PD-1 (Figure 4C).

We annotated five clusters of “cycling” neoAg-specific CD8 T cells displaying a range of activation states (Figure 3G; Table S1). The frequency of total cells within cycling clusters was modestly increased by anti-CTLA-4 or anti-PD-1 ICT, whereas combination ICT decreased the frequency (Figure S7B). Within nAg.Cd8_Cycling__1, nAg.Cd8_Cycling__3, and nAg.Cd8_Cycling__4, either control vax or neoAg SLP vax increased the frequency of neoAg-specific CD8 T cells compared to control mAb (Figure 4C). nAg.Cd8_Cycling__2 represented 3.79% of neoAg-specific CD8 T cells with control mAb treatment and 10.6% under control vax conditions, whereas under neoAg SLP vax conditions, the frequency of cells within this cluster increased to 19.2% of neoAg-specific CD8 T cells (Figure 4C). Compared to the other cycling clusters, nAg.Cd8_Cycling__2 expressed higher *Tnfrsf4* (OX40), *Tnfrsf9* (4-1BB), *Prf1*, and *Ifng* (Figures 3G and S6B). Although both control vax and neoAg SLP vax promoted cycling neoAg-specific CD8 T cells, far more neoAg-specific CD8 T cells were observed within tumors treated with neoAg SLP vax compared to control vax (Figures 3C and 3D). These differences are likely because under control vax, significantly more intratumoral CD8 T cells are undergoing apoptosis than with neoAg SLP vax or control mAb (Figure S7C).

Most neoAg-specific CD8 T cells expressed PD-1 protein, with similar frequencies of PD-1^+^TIM-3^+^ or PD-1^+^LAG-3^+^ neoAg-specific CD8 T cells observed between the different ICT treatment conditions (Figures 4D and S6C). Expression of PD-1 on a per-cell basis was lower in ICT-treated groups. In contrast, an increase in the percentage of PD-1^+^TIM-3^+^ or PD-1^+^LAG-3^+^ neoAg-specific CD8 T cells was observed in mice treated with neoAg SLP vax compared to control mAb or control vax (Figures 4D and S6C). Intracellular cytokine staining (ICS) on isolated intratumoral CD8 T cells restimulated with the mLama4 neoAg peptide revealed that neoAg SLP vax or anti-CTLA-4 increased the frequency of IFN-γ^+^ or tumor necrosis factor (TNF)-α^+^ CD8 T cells, with neoAg SLP vax inducing the greatest expansion (>5-fold) compared to control mAb or control vax (Figure 4E). Among mLama4 neoAg-stimulated IFN-γ^+^ CD8 T cells, expression of IFN-γ increased significantly with anti-CTLA-4 and/or anti-PD-1, with neoAg SLP vax prompting the most robust increase (Figure 4E).

### Anti-CTLA-4 promotes Th1-like CD4 T cells expressing ICOS and Bhlhe40

**Figure 5.**
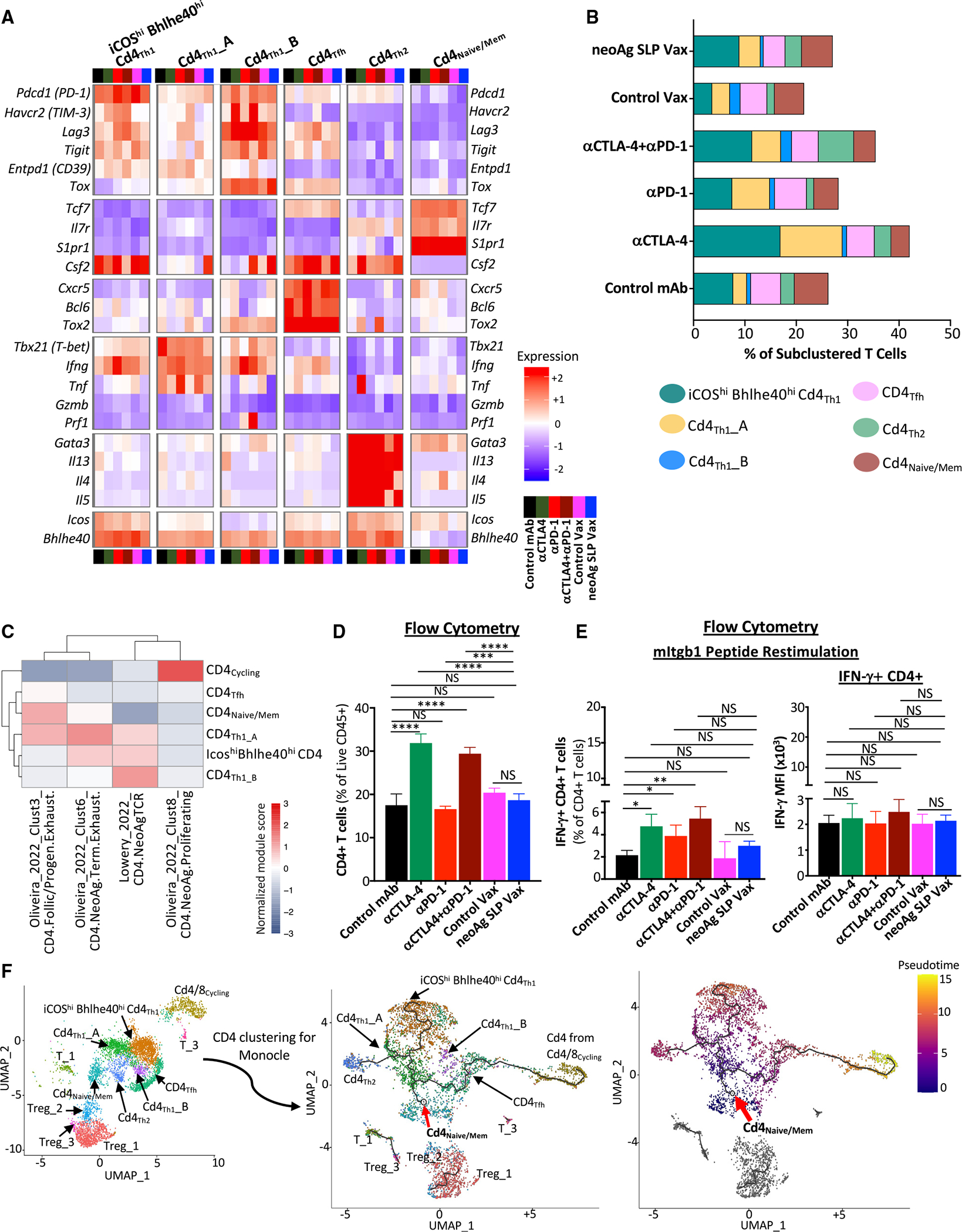
Anti-CTLA-4 induces ICOS^+^Bhlhe40^+^ Th1-like CD4 T cells (A) Heatmap displaying normalized expression of select genes in each CD4 T cell cluster by treatment. (B) Bar graphs depicting frequency of CD4 T cells within each cluster by treatment. (C) Heatmap comparing features (module scores) of CD4 T cell clusters (rows) to published human CD4 T cell gene signatures (columns) of neoAg-specific CD4 T cells identified/annotated by Oliveira et al.^67^ and Lowery et al.^61^ (D) CD4 T cell frequency in Y1.7LI tumors as determined by flow cytometry. (E) Graph displaying IFN-γ^+^ CD4 T cells and IFN-γ MFI of IFN-γ^+^ CD4 T cells as assessed by ICS on mItgb1 peptide-restimulated CD4 T cells isolated from Y1.7LI tumors. (F) UMAP plot displaying exclusively CD4 T cell-containing clusters (left). Monocle 3-guided CD4 T cell trajectory graph overlaid on UMAP (middle) (red arrow indicates inferred pseudotime origin). CD4 T cell clusters overlaid on Monocle3 pseudotime plot (right). For (D) and (E), mice were treated beginning on day 7 post-tumor transplant and harvested on day 15. Bar graphs display the mean ± SEM and are representative of at least three independent experiments (*p < 0.05, **p < 0.01, ***p < 0.005, ****p < 0.0001; NS, not significant, unpaired t test). See also Figure S8 and Table S2.

Since effective neoAg SLP vax or ICT required not only CD8, but also CD4, T cells (Figure S2A), we examined CD4 T cells. Anti-CTLA-4 induced a higher frequency of conventional CD4 T cells and reduced the percentage of Tregs as assessed by scRNA-seq of CD45^+^ cells and flow cytometry (Figures S3A, S3B, S4D, and S4F). Notably, anti-CTLA-4 (±anti-PD-1) induced subpopulations of Th1-like cells^63,64^ expressing *Ifng* and *Bhlhe40*, including cluster ICOS^hi^Bhlhe40^hi^CD4_Th1_, which also highly expressed *Icos*,^25,65,66^ *Pdcd1*, *Ctla4*, *Cxcr6*, *Csf2* (GM-CSF), *Fasl*, *Furin*, and *Tnfaip3* (Figures 2E, 5A, 5B, and S4A). ICOS^hi^Bhlhe40^hi^ CD4_Th1_ displayed enrichment in interleukin (IL)-2 STAT5 and IL-6 JAK STAT3 signaling, TNF-α signaling via NF-κB, and IFN-γ response gene sets, among others (Figure S8A). Although neoAg SLP vax exhibited a greater frequency of cells within this cluster compared to control vax, the frequency under neoAg SLP vax conditions was similar to that of control mAb (Figure 5B). Cd4_Th1__A also expressed *Icos* and *Bhlhe40*, but to less of an extent than ICOS^hi^Bhlhe40^hi^CD4_Th1_, and was further distinguished by lower *Furin*, *Cxcr6*, *Runx3*, *Tnfaip3*, *Pdcd1*, *Havcr2*, and *Lag3* expression and higher *Tbx21* expression (Figures 5A and S4A). While anti-CTLA-4 and/or anti-PD-1 or neoAg SLP vax increased the frequency of CD4_Th1__A, the increase was most pronounced with anti-CTLA-4 (Figure 5B). CD4_Th1__B was the smallest Th1-like cluster, and only subtle changes to its frequency occurred with treatment apart from control vax and combination ICT, where an increase was observed (Figure 5B). Comparison with published gene signatures of neoAg-specific CD4 T cells indicated that ICOS^hi^Bhlhe40^hi^CD4_Th1_, CD4_Th1__A, and CD4_Th1__B displayed enrichment in signatures of tumor-specific CD4 T cells (Lowery_2022_CD4.neoAgTCR) derived from transcriptomic analysis of neoAg-specific TCR clonotypes from human metastatic tumor samples^61^ (Figure 5C). ICOS^hi^Bhlhe40^hi^CD4_Th1_, CD4_Th1__A, and CD4_cycling_ displayed features of neoAg-specific conventional CD4 T cells (isolated from human melanoma) that were previously described and phenotypically annotated (Oliveira_2022_Clust6_CD4.neoAg.Term.Exhaust, Oliveira_2022_Clust3_CD4.Follic/Progen.Exhaust, and Oliveira_2022_Clust8_CD4.neoAg.Proliferating)^67^ (Figure 5C).

The increase in IFN-g-expressing Th1-like cells most prominently induced by anti-CTLA-4 was reflected by ICS on intratumoral CD4 T cells restimulated *ex vivo* with the mItgb1 MHC-II neoAg peptide. Anti-CTLA-4 (±anti-PD-1) induced the strongest increase in the overall frequency of conventional CD4 T cells and IFN-γ^+^ CD4 T cells (Figures 5D and 5E), with anti-PD-1 also increasing IFN-γ ^+^ CD4 T cells (Figure 5E). Interestingly, under combination ICT, a small cluster (Cd4_Th2_) expressing *Icos* and *Bhlhe40*, as well as *Furin*, *Tnfaip3*, *Cd28*, and *Il7r*, was noted. Un-like the other ICOS^+^Bhlhe40^+^ clusters, *Ifng*, *Havcr2*, and *Lag3* were barely detectable, and instead, Cd4_Th2_ expressed *Gata3*, *Il4*, *Il5*, and *Il13*, indicative of Th2-like cells^63,64^ (Figures 5A, S4A, and S8B).

Monocle^68^ pseudotime/trajectory analysis of scRNA-seq data suggested that the starting point for conventional CD4 T cells corresponds to cells within cluster Cd4_Naive/Mem_ (expressing *Tcf7*, *Il7r*, and *S1pr1*) or CD4 T cells within cluster Cd4/8_Cycling_ (Figure 5F) with Cd4_Tfh_ (displaying T follicular helper-like transcriptional features^64^) connecting Cd4/8_Cycling_ CD4 T cells to the main trajectory toward Cd4_Naive/Mem_ and the branch to more activated, polarized CD4 T cells. Notably, a pseudotime trajectory branchpoint occurs whereby activated CD4 T cells occupy Th1-like ICOS^hi^Bhlhe40^hi^Cd4_Th1_ driven by anti-CTLA-4 (±anti-PD-1) or encounter another branch whereby they become either Th1-like cells within Cd4_Th1__A or Th2-like Cd4_Th2_, with Cd4_Th1__A being induced by anti-CTLA-4 and/or anti-PD-1 or neoAg SLP vax and Cd4_Th2_ primarily being driven by combination anti-CTLA-4 and anti-PD-1 (Figure 5F).

We also identified three Treg clusters (Figure S3B). Treg_1 and Treg_3 appeared to be the most activated (Figure S4A). Mice treated with anti-CTLA-4 ± anti-PD-1 experienced a decrease in the frequency of Treg_1 and Treg_3 (Figure S3B). The anti-CTLA-4 mAb we used (mouse IgG2b; clone 9D9) is known to partially deplete Tregs, especially those highly expressing CTLA-4.^22,24–26,69–71^ Alterations to the overall frequency of Tregs most prominently observed with anti-CTLA-4 were corroborated by flow cytometry analysis (Figure S3A).

### Intratumoral myeloid compartment during neoAg SLP vaccine or ICT treatment

**Figure 6.**
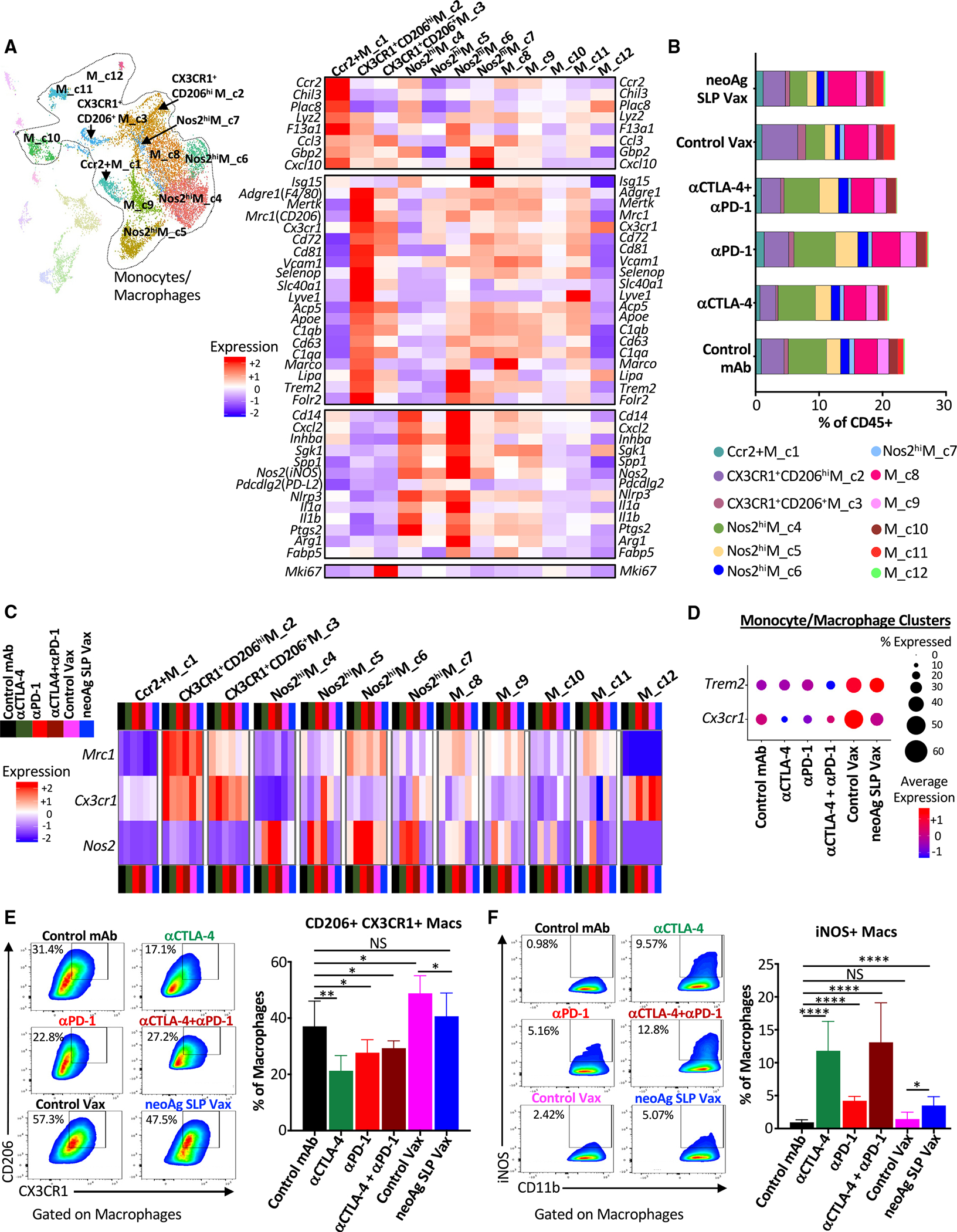
ICT promotes partially distinct macrophage remodeling from neoAg SLP vaccines (A) UMAP displaying subclustering of select myeloid clusters from CD45^+^ scRNA-seq and heatmap displaying normalized expression of select genes by monocyte/macrophage cluster. (B) Bar graphs depicting frequency of monocytes/macrophages in each cluster by treatment. (C) Heatmap displaying normalized expression of *Mrc1* (CD206), *Cx3cr1*, and *Nos2* (iNOS) in each monocyte/macrophage cluster by treatment. (D) scRNA-seq dot plot depicting *Trem2* and *Cx3cr1* expression in combined monocyte/macrophage clusters. (E and F) Representative flow cytometry plots and graphs displaying intratumoral (E) CX3CR1^+^CD206^+^ macrophages or (F) iNOS^+^ macrophages from Y1.7 melanoma-bearing mice treated beginning on day 7 post-tumor transplant and harvested on day 15. For (E) and (F), dot plots displaying CX3CR1^+^CD206^+^ and iNOS^+^ macrophages are gated on macrophages, and bar graphs display the mean ± SEM and are representative of at least three independent experiments (*p < 0.05, **p < 0.01, ****p < 0.0001; NS, not significant, unpaired t test). See also Figures S10 and S12.

To characterize monocytes/macrophages and DCs, we subclustered myeloid cell-containing clusters excluding the single neutrophil cluster (Figures 2B, 2C, 6A, and S9A). In addition to a plasmacytoid DC (pDC) cluster, four other DC clusters were identified (Figures S9A–S9E). Cluster CD103^+^cDC1 expressed multiple cDC1 transcripts including *Itgae* (*Cd103*), *Xcr1*, and *Clec9a* (Figures S9B and S9E). CD63^+^Ccr7^+^cDC and Ccr7^+^cDC expressed *Ccr7*, *Cd1d1*, *Cd200*, *Fscn1*, *Cd274* (PD-L1), and *Pdcd1lg2* (PD-L2). Compared to Ccr7^+^cDC, CD63^+^Ccr7^+^cDC expressed higher *Cd63*, *Cd40*, *Btla*, and *Cd70* (Figures S9D and S9E). These two migratory cDC clusters are consistent with mregDCs, which are cDC1s and cDC2s that express maturation as well as immunoregulatory markers (although they are not necessarily immunosuppressive).^72,73^ In addition, two small undefined clusters expressed transcripts predominantly associated with non-myeloid cells and instead expressed by lymphocytes/ILCs (Undef_1) and Tregs (Undef_2).

### Distinct macrophage remodeling induced by neoAg SLP vaccines and ICT

Monocytes/macrophages represented a plurality of intratumoral CD45^+^ cells and displayed a range of phenotypic states^74,75^ (Figures 6A, S3A, and S10A–S10C). Ccr2^+^M_c1 was a small (~1% of myeloid compartment) cluster that displayed transcripts consistent with monocytes, including *Ccr2* and *Chil3*, and the frequency of cells within this cluster remained largely unchanged (Figures 6A, 6B, and S10C).

Macrophages within CX3CR1^+^CD206^hi^M_c2 highly expressed *Cx3cr1* (fractalkine receptor transcript), *Mrc1* (CD206), *Trem2*,^76–78^ *Vcam1*, *Cd63*, and *Cd72*. A reduced frequency of CX3CR1^+^CD206^hi^M_c2 macrophages was observed with anti-CTLA-4 ± anti-PD-1 compared to control mAb, with expression of *Cx3cr1* within this cluster decreasing under all ICT treatment conditions or with neoAg SLP vax (Figures 6A–6C, S10A, and S10C). CX3CR1^+^CD206^+^ M_c3 was a small cluster that also expressed *Cx3cr1*, as well as *Mrc1*, *Trem2*, *Vcam1*, and *Cd72*, with the latter transcripts being expressed less than in CX3CR1^+^CD206^hi^M_c2 (Figure 6A). CX3CR1^+^CD206^+^M_c3 also displayed high expression of *Mki67*. M_c8 macrophages expressed *Cx3cr1* and *Mrc1* under control mAb conditions, with ICT reducing expression of *Cx3cr1* within these clusters (Figures 6C and S10A). Other clusters also expressed *Cx3cr1* under certain treatment conditions, but overall, monocytes/macrophages from ICT-treated mice displayed reduced expression of *Cx3cr1* compared to control mAb (Figures 6A, 6C, 6D, S10A, and S10C). In contrast, monocytes/macrophages from control vax and neoAg SLP vax groups displayed similar or even higher expression of *Cx3cr1* compared to control mAb. It was also notable that *Trem2* was expressed highest in control vax and neoAg SLP vax groups, with macrophages co-expressing *Cx3cr1* and *Mrc1* also expressing *Trem2* (Figures 6D and S10C).

Several monocyte/macrophage clusters expressed *Nos2* (iNOS) (Figures 6C, S10B, and S10C). While the overall frequency of these iNOS^+^ M1-like clusters only modestly increased with ICT, the frequency of cells within these clusters expressing *Nos2* and/or *Nos2* expression increased under all ICT conditions (Figures 6B, 6C, and S10B). Nos2^hi^M_c4 and Nos2^hi^M_c6 expressed *Nos2*, *Il1a*, *Il1b*, *Cxcl2*, *Inhba*, and *Nfkb1*, signatures of inflammatory macrophages (Figures 6A and S10C). While Nos2^hi^M_c4 displayed classic features of M1-like macrophages, including low *Mrc1* expression, Nos2^hi^ M_c6 moderately expressed *Mrc1* and exhibited higher *F13a1*, *Trem2*, and *Il1a* compared to Nos2^hi^M_c4 (Figures 6A and S10B). Nos2^hi^M_c4 displayed high expression of *Cxcl9* and *Spp1*,^79^ with expression of the latter diminished with ICT or neoAg SLP vax (Figure S10C). Nos2^hi^M_c5 highly expressed *Nos2* in the presence of ICT, with ICT also increasing the frequency of macrophages within this cluster (Figures 6B, 6C, and S10B). This cluster also expressed moderate levels of *Mki67* and other cellcycle-related transcripts, indicative of iNOS^+^ macrophages with proliferative capabilities (Figures 6A and S10C). Nos2^hi^M_c7 was the smallest iNOS^+^ macrophage cluster and, in addition to *Nos2* expression under ICT conditions, Nos2^hi^M_c7 expressed IFN-stimulated genes (ISGs) (Figures 6A, 6C, S10B, and S10C).

These same overall patterns were manifested at the protein level, where in anti-CTLA-4- and/or anti-PD-1-treated mice, the frequency of intratumoral CX3CR1^+^CD206^+^ macrophages decreased with a concomitant increase in iNOS^+^ macrophages (Figures 6E and 6F), consistent with our findings in mouse MCA sarcomas.^22,24^ While neoAg SLP vaccine-treated mice also displayed a greater frequency of iNOS^+^ macrophages compared to control mAb or control vax, CX3CR1^+^CD206^+^ macrophages were only slightly reduced by neoAg SLP vax compared to control vax but were maintained at a frequency similar to that seen in control mAb-treated mice (Figures 6E and 6F). These results reveal that, despite a relatively similar abundance of CX3CR1^+^CD206^+^ macrophages that were previously associated with progressively growing tumors,^22,24^ neoAg SLP vax induces tumor regression equivalent to ICT when initiated at day 7 post-transplant.

### ICT and myeloid-targeting strategies broaden the therapeutic window for neoAg SLP vaccines

We noted changes that were not only shared between treatment conditions, but also distinct depending upon which treatment was employed, prompting us to ask whether neoAg SLP vax could synergize with ICT. While neoAg SLP vax or ICT led to robust rejection of Y1.7LI when initiated on day 7 post-transplant (Figure 1D), a majority of mice displayed tumor outgrowth when treatment with anti-CTLA-4, anti-PD-1, or neoAg SLP vax was initiated on day 12 post-transplant (Figure S11A). We therefore used a day 12 treatment start to assess whether combining neoAg SLP vax with anti-CTLA-4 or anti-PD-1 improved efficacy. Mice treated with neoAg SLP vax in combination with anti-CTLA-4 or anti-PD-1 displayed enhanced tumor control compared to control vax + anti-PD-1 or control vax + anti-CTLA-4 (Figure S11A). Further, neoAg SLP vax used in combination with anti-CTLA-4 or anti-PD-1 provided superior tumor growth inhibition compared to combination anti-CTLA-4 and anti-PD-1. We also assessed neoAg SLP vax and ICT combination therapy using the MC38 tumor model, which has several known endogenous MHC-I tumor neoAgs^20,80,81^ that we previously confirmed were expressed in our MC38 line. As with Y1.7LI, neoAg SLP vax in combination with anti-CTLA-4 or anti-PD-1 provided superior protection versus monotherapy against MC38 outgrowth (Figure S11B).

**Figure 7.**
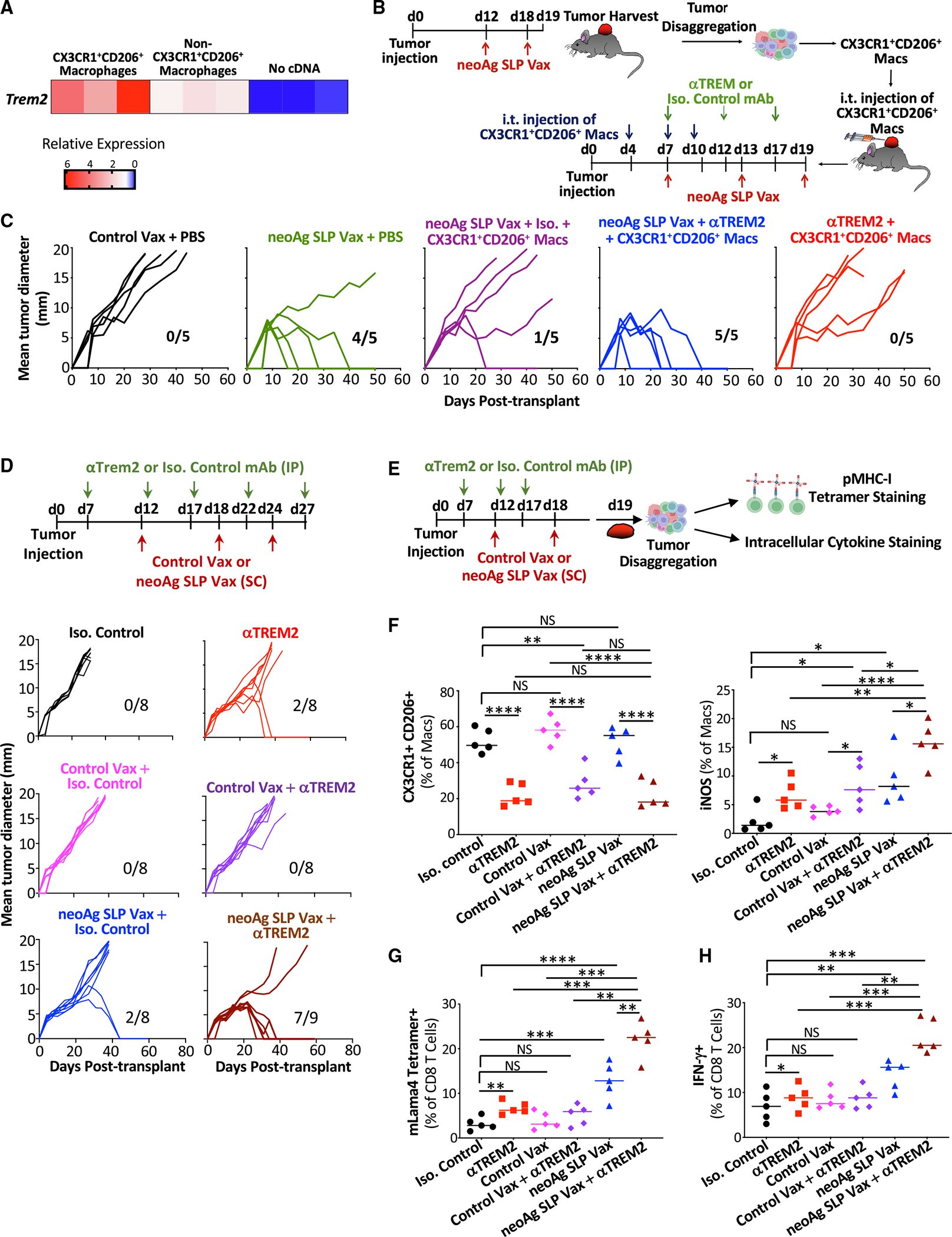
Blockade of TREM2 remodels the macrophage compartment and facilitates anti-tumor immunity in combination with neoAg SLP vaccines (A) *Trem2* mRNA detection by quantitative reverse-transcriptase PCR (qRT-PCR) on sorted intratumoral CX3CR1^+^CD206^+^ macrophages and non-CX3CR1^+^CD206^+^ macrophages isolated on day 19 post-tumor transplant from Y1.7LI tumor-bearing mice treated with neoAg SLP vax on days 12 and 18. (B) Schematic depicting the experiment in (C). (C) Tumor growth in mice transplanted with Y1.7LI melanoma cells and receiving intratumoral injections of CX3CR1^+^CD206^+^ macrophages or PBS and treated with control vax, neoAg SLP vax, anti-TREM2, neoAg SLP vax + isotype control mAb (Iso), or neoAg SLP vax + anti-TREM2 as indicated in (B). (D) Tumor growth in Y1.7 melanoma-bearing mice treated with Iso, anti-TREM2, Iso + control vax, Iso + neoAg SLP vax, anti-TREM2 + control vax, or anti-TREM2 + neoAg SLP vax. (E) Schematic depicting experiments in (F)–(H). (F) Graphs displaying frequency of intratumoral CX3CR1^+^CD206^+^ macrophages and iNOS^+^ macrophages. (G) Graph displaying frequency of mLama4 tetramer-positive CD8 T cells. (H) Graph displaying IFN-g^+^ CD8 T cells as assessed by ICS of mLama4 peptide-restimulated CD8 T cells isolated from Y1.7LI tumors. For (A), RNA was isolated from macrophages from two individual mice (two independent experiments). For (C) and (D), fractions indicate number of mice rejecting tumors/number of mice used in the experiment. Scatterplots in (F)–(H) display data for individual mice and are representative of two independent experiments (*p < 0.05, **p < 0.01, ***p < 0.001, ****p < 0.0001; NS, not significant, unpaired t test).

We were struck by the notable presence of intratumoral CX3CR1^+^CD206^+^ macrophages that also express the TREM2 receptor transcript in neoAg SLP vax-treated mice (Figures 6A and 6D). We reasoned that CX3CR1^+^CD206^+^ macrophages might play a role in blunting neoAg SLP vaccine efficacy in the day 12 post-transplant treatment initiation setting. In the day 12 neoAg SLP vax setting, expression of *Trem2* was indeed enriched on sorted intratumoral CX3CR1^+^CD206^+^ macrophages (compared to the non-CX3CR1^+^CD206^+^ macrophages) (Figure 7A). We intratumorally injected sorted CX3CR1^+^CD206^+^ macrophages from day 12 neoAg SLP vax-treated mice into a separate cohort of Y1.7LI tumor-bearing mice on days 4, 7, and 10 post-transplant and initiated neoAg SLP vax on day 7 (Figure 7B). As expected, neoAg SLP vax initiated on day 7 induced tumor regression in the absence of additional exogenous CX3CR1^+^CD206^+^ macrophages (Figure 7C). In contrast, intratumoral injection of additional CX3CR1^+^CD206^+^ macrophages rendered neoAg SLP vax initiated on day 7 ineffective. The inhibition of neoAg SLP vax efficacy by the injected CX3CR1^+^CD206^+^ macrophages was reversed by giving a non-depleting TREM2-blocking mAb. We previously demonstrated that anti-TREM2 reshaped tumor-associated macrophages, reducing CX3CR1^+^CD206^+^ macrophages while concomitantly expanding macrophages expressing immunostimulatory molecules.^77^ We hypothesized that targeting the CX3CR1^+^CD206^+^ macrophages in our vaccine setting could extend the therapeutic window for neoAg SLP vaccines. Indeed, we found that anti-TREM2 used in combination with neoAg SLP vax (initiated on day 12 post-transplant) enhanced efficacy in Y1.7LI tumor-bearing mice (Figure 7D). This enhanced efficacy was associated with a reduction of intratumoral CX3CR1^+^ CD206^+^ macrophages, promotion of iNOS^+^ macrophages, and increased IFN-γ^+^ mLama4 neoAg-specific CD8 T cells (Figures 7E–7H). Altogether, these findings support the rationale for combination neoAg-based therapies, including those targeting the myeloid compartment.

## DISCUSSION

In this study, we compared distinct immunotherapies in mouse melanoma models with relevant genetic perturbations^52^ and defined neoAgs. Although prior studies have explored neoAg vaccines,^16,17,20,82–85^ few compared them extensively to different ICTs as we did in this study, making several key observations. First, neoAg SLP vaccines and ICT work by mechanisms related to the CD8 T cell response, with key differences in the overall magnitude of the response and phenotype of neoAg-specific CD8 T cells observed. NeoAg SLP vaccines induce the greatest expansion of functional intratumoral neoAg-specific CD8 T cells, including proliferating and PD-1^+^TCF-1^+^ stem-like (Pex/Pex-like) CD8 T cells. Systemic nanoparticle neoAg vaccines were also shown to generate TCF-1^+^ stem-like neoAg-specific CD8 T cells in mice.^83^ Anti-PD-1, especially in combination with anti-CTLA-4, induced Bhlhe40^hi^ neoAg-specific CD8 T cells. We previously found that ICT promotes Bhlhe40 upregulation in tumor neoAg-specific T cells and that expression of Bhlhe40 in CD4 and/or CD8 T cells is vital for effective anti-CTLA-4 or anti-PD-1 ICT,^24^ with a separate study also demonstrating that Bhlhe40 is critical in CD8 T cells for anti-PD-1/PD-L1 efficacy.^86^ A more recent study identified Bhlhe40 as modulating a key differentiation point between progenitor and intermediate subsets of exhausted T cells in an *in vitro* exhaustion model and chronic lymphocytic choriomeningitis virus (LCMV) infection.^87^ Altogether, our data suggest that anti-PD-1 predominantly provokes neoAg-specific effector CD8 T cells, and anti-CTLA-4, either directly or indirectly through induction of CD4 T helper cells, promotes neoAg-specific effector CD8 T cells with reduced expression of inhibitory receptors. In contrast, the predominant mechanisms underlying neoAg vaccines are likely driven by activation of naive CD8 T cells to PD-1^+^TCF-1^+^ stem-like/Pex-like CD8 T cells, which may provide a source of precursor cells that become PD-1^+^TCF-1^−^ CD8 effector T cells and robust expansion of neoAg-specific CD8 T cells.

In addition to modulating CD8 T cells, ICT impacted the CD4 T cell compartment as well. Anti-CTLA-4 notably induced ICOS^+^ Th1-like CD4 T cells displaying high expression of Bhlhe40,^24^ again consistent with a critical role for Bhlhe40 in not only CD8, but also CD4, T cells. Bhlhe40 has been shown to promote Th1 cells,^88–90^ and subsets of Bhlhe40^+^ Th1-like CD4 T cells were found to be enriched in patients with colorectal cancer with microsatellite instability, which display more favorable outcomes in response to anti-CTLA-4.^91^ Further, studies in both preclinical models and patients with melanoma revealed that anti-CTLA-4 induces ICOS^+^ CD4 T cells expressing IFN-g,^65,66^ together suggesting the human relevance of our findings.

Although CD4 T cells and MHC-II neoAgs are critical components of anti-tumor immunity,^23,27,28,56,92–94^ we chose to utilize an SLP vaccine against a single MHC-I neoAg to definitively link the MHC-I neoAg vaccine response to a specific defined neoAg. The neoAg SLP vaccines we used predominantly altered CD8 T cells, although these neoAg SLP vaccines still require CD4 T cells for efficacy. While SLPs offer several advantages over short peptides, including the potential to provoke both CD4 and CD8 T cell responses,^95,96^ the neoAg SLPs we used (mAlg8/mLama4) provoke only neoAg-specific CD8 T cell responses.^16^ Whether incorporating an MHC-II neoAg such as mItgb1 enhances the efficacy of neoAg vaccines in our models is of future interest. A recent study found that inclusion of low doses of MHC-II-restricted neoAg SLPs (along with MHC-I-restricted neoAg SLPs) promoted tumor rejection, whereas neoAg SLP vaccines containing higher concentrations of the same MHC-II neoAg induced type 1 regulatory T cells (Tr1) and blunted tumor rejection.^97^ Although this inhibition could be overcome with additional treatment modalities, it nevertheless suggests that inclusion of MHC-II neoAgs improves vaccine efficacy, but the dose is critical.

While certain alterations induced by combination ICT were distinct from either anti-CTLA-4 or anti-PD-1, several features of combination ICT were also observed with anti-CTLA-4, whereas other changes were more akin to those observed with anti-PD-1. These findings add to the accumulating evidence that the enhanced anti-tumor activity of combination anti-CTLA-4 and anti-PD-1 is mediated not only through additive effects, but also through mechanisms distinct from the monotherapies.^22,26,98^ While anti-CTLA-4 and anti-PD-1 combination ICT outperforms monotherapy in patients with metastatic melanoma, immune-related adverse events are problematic.^99–102^ We found that neoAg SLP vaccines combined with either anti-CTLA-4 or anti-PD-1 drove anti-tumor responses against Y1.7LI or MC38 that were equal to or better than combination ICT. Therefore, combining neoAg vaccines with favorable safety profiles^6,7,9^ along with single-agent ICT^83–85,103,104^ may yield robust anti-tumor immunity with less toxicity.

Beyond T cells, we noted that both ICT and neoAg SLP vax increased M1-like iNOS^+^ macrophages. Whereas ICT reduced the frequency of intratumoral M2-like CX3CR1^+^CD206^+^ macrophages, neoAg SLP vaccine-treated mice displayed an equal or greater frequency of CX3CR1^+^CD206^+^ macrophages compared to control mAb- or ICT-treated mice, albeit less than with control vax. Our study (in particular the scRNA-seq data) also supports the concept that, although macrophages may have “M1-like” or “M2-like” features, they display a spectrum of activation states and do not fit exclusively into M1/M2 states.^74,105^

The differences noted between neoAg SLP vaccines and ICT on the macrophage compartment likely involve multiple signals within the TME. We previously found that ICT-driven induction of iNOS^+^ macrophages was dependent upon IFN-γ, whereas ICT-driven depletion of CX3CR1^+^CD206^+^ macrophages was partially independent of IFN-γ.^22^ In our vaccine setting, we hypothesize that T cell-derived IFN-γ and other factors drive monocyte polarization to iNOS^+^ macrophages upon entering the tumor, but other signals promote CX3CR1^+^CD206^+^ macrophages as well. These signals are yet unknown but are likely induced by the pI:C (contained in control vax and neoAg SLP vax), which acts as an endosomal TLR3 agonist to induce a type I IFN response and can also activate RIG-I/MDA-5 in the cytosol to promote IL-12 production.^106,107^ Although type I IFN is usually associated with M1-like inflammatory macrophages, a recent study found that pI:C-induced type I IFN unexpectedly provoked IL-4 production by monocytes and skewed tumor macrophages toward an M2-like phenotype.^108^ In the MC38 tumor model, the Seder group recently found that a systemic neoAg-TLR7/8 agonist nanoparticle vaccine altered the intratumoral monocyte/macrophage compartment by potently reducing *Chil3*^*+*^ monocytes.^82^ We did not observe neoAg SLP vax-induced alterations in Chil3^+^ monocytes, which represented ~1% of cells in Y1.7LI, much less than in MC38.

CX3CR1^+^CD206^+^ macrophages from neoAg SLP vaccine-treated mice displayed high expression of the transcript for TREM2, a myeloid cell receptor^109^ that recently emerged as a regulator of macrophage function in tumors.^76–78^ TREM2 is widely expressed on macrophages in human tumors, and expression of TREM2 is generally associated with worse prognosis.^76,110^ Intratumoral injection of CX3CR1^+^CD206^+^ macrophages rendered neoAg SLP vax initiated on day 7 ineffective, providing evidence that the CX3CR1^+^CD206^+^ macrophages are indeed immunosuppressive and that TREM2 is a relevant target, since effects of the injected CX3CR1^+^CD206^+^ macrophages could be reversed by a non-depleting TREM2-blocking mAb. However, it is possible that TREM2 blockade is also affecting endogenous macrophages in this context. We previously demonstrated that targeting TREM2 with a non-depleting anti-TREM2 mAb reduced CX3CR1^+^CD206^+^ macrophages while concomitantly expanding macrophages expressing immunostimulatory molecules.^77^ Further, anti-PD-1 treatment along with TREM2 deficiency in mice alters the gut microbiome and induces proinflammatory programs in intestinal macrophages to enhance anti-PD-1 efficacy.^111^ We hypothesized that combining neoAg SLP vaccines that maintain or promote CX3CR1^+^CD206^+^ macrophages expressing TREM2 with treatments targeting this macrophage population^77,78^ might enhance the efficacy of neoAg SLP vaccines. Indeed, we found that anti-TREM2 mAb used in combination with neoAg SLP vax enhanced efficacy. This enhanced efficacy was associated with a reduction of intratumoral CX3CR1^+^CD206^+^ macrophages, promotion of iNOS^+^ macrophages, and increased IFN-g^+^ mLama4 neoAg-specific CD8 T cells.

This study provides key insights into the changes that occur within major immune cell populations within the TME following different forms of cancer immunotherapy. Although we did not fully elaborate on every immune cell population, the myeloid and lymphoid subsets and potential biomarkers we have described herein should inform the development of improved personalized neoAg vaccines and combinatorial therapies in patients.

### Limitations of the study

This study offers insights into processes underlying different forms of ICT and neoAg SLP cancer vaccines, uncovering rational combination therapies, including those targeting the immunosuppressive myeloid compartment. While we focused on mLama4/mAlg8/mItgb1 neoAg-specific T cells, we did not elaborate on how much of the T cell response is tumor reactive or determine whether T cell responses to other yet undefined tumor antigens are relevant. Although we compared our T cell subpopulations to those in humans, the applicability of our findings to patients needs validation. Further, many vaccine platforms exist, including RNA-based vaccines, and our results may be more specific to SLP plus pI:C vaccines. Although vaccinating against a single neoAg with Y1.7LI/Y1.7AI and three with MC38 was efficacious, targeting multiple neoAgs and possibly non-mutant antigens will likely be required in patients due to tumor heterogeneity and therapy-induced immunoediting.^112,113^

### RESOURCE AVAILABILITY

#### Lead contact

Requests for further information and resources and reagents should be directed to and will be fulfilled by the lead contact, Matthew Gubin (mgubin@mdanderson.org).

#### Materials availability

Modified mouse melanoma lines and plasmids generated in this study are available from the lead contact upon request.

#### Data and code availability

- scRNA-seq data have been deposited with NCBI GEO under accession nos. GEO: GSE276902 and GEO: GSE276904 and are publicly available as of the date of publication.
- This paper does not report original code.
- Any additional information required to reanalyze the data reported in this paper is available from the lead contact upon request.

## STAR★METHODS

Detailed methods are provided in the online version of this paper and include the following:

### EXPERIMENTAL MODEL AND STUDY PARTICIPANT DETAILS

#### Mice

All mice used were on a C57BL/6J background. Wildtype (WT) C57BL/6J mice were purchased from Jackson Labs. All *in vivo* experiments used 8- to 12-week-old male or female mice (to match the sex and strain of the tumors). Mice were housed in a specific pathogen-free animal facility. All animal studies were performed in accordance with, and with the approval of the Institutional Animal Care and Use Committee (IACUC) of The University of Texas MD Anderson Cancer Center (Houston, TX).

#### Plasmids

Gene blocks for mAlg8 + mItgb1 or mLama4 + mItgb1 were purchased from Integrated DNA Technologies. Minigene constructs were cloned into the BglII site of pMSCV-IRES GFP using the Gibson Assembly method (New England Biolabs). To generate neoantigen-expressing Y1.7 melanoma cell lines, constructs were transiently transfected into Phoenix Eco cells using Fugene (Promega). After 48 hours, viral supernatants were filtered and subsequently used for infection of *Braf*^*V600E*^ *Cdkn2a*^−/−^ *Pten*^−/−^ YUMM1.7 parental line (Y1.7). Y1.7 mLama4 ^MHC-I^.mItgb1 ^MHC-II^ (Y1.7LI) and Y1.7 mAlg8 ^MHC-I^.mItgb1 ^MHC-II^ (Y1.7AI) were sorted based on GFP positivity and clones were verified for neoantigen expression.

#### Tumor cell lines

The *Braf*^*V600E*^ *Cdkn2a*^−/−^ *Pten*^−/−^ YUMM1.7 parental line was originally generated in a male GEMM as described^45^. Parental YUMM1.7 was purchased from ATCC (CRL-3362) and was modified to generate neoAg-expressing Y1.7 lines. The MC38 line was obtained from B. Schreiber (Washington University in St. Louis School of Medicine). All tumor cell lines were found to be free of common mouse pathogens and Mycoplasma as assessed by IDEXX IMPACT I mouse pathogen testing [PCR evaluation for: Corynebacterium bovis, Corynebacterium sp. (HAC2), Ectromelia, EDIM, Hantaan, K virus, LCMV, LDEV, MAV1, MAV2, mCMV, MHV, MNV, MPV, MTV, MVM, Mycoplasma pulmonis, Mycoplasma sp., Polyoma, PVM, REO3, Sendai, TMEV] in December 2023. Tumor cell lines from the same cryopreserved stocks that were used in this study tested negative for Mycoplasma and were authenticated and found to be free of non-mouse cells as assessed by mouse cell STR profiling (IDEXX CellCheck mouse 19 plus Mycoplasma spp. testing) in December 2023.

### Tumor transplantation

The Phoenix Eco cells, *Braf*^*V600E*^ *Cdkn2a*^−/−^ *Pten*^−/−^ YUMM1.7 parental melanoma, Y1.7LI melanoma, Y1.7AI melanoma, and the MC38 colorectal cancer cell lines were propagated in R-10 plus BME media [RPMI media (HyClone) supplemented with 1% l-glutamine, 1% penicillin–streptomycin, 1% sodium pyruvate, 0.5% sodium bicarbonate, 0.1% 2-mercaptoethanol, and 10% heat-inactivated fetal bovine serum (FBS) (HyClone) upon thawing. Tumor cell lines were passaged 3 to 6 times before experimental use. Prior to injection, cells were washed extensively, resuspended at a concentration of 2.33 × 10^6^ cells/ml; for YUMM1.7 and Y1.7AI; 2.33 × 10^6^, 3.33 × 10^6^, or 5.0 × 10^6^ cells/ml for Y1.7LI; or 10 × 10^6^ cells/ml for MC38 in 150 μL of endotoxin-free PBS and 150 mL was injected subcutaneously into flank of recipient mice. The viability of tumor cells at the time of injection was >90% as assessed by Trypan blue exclusion assay. Tumor growth was quantified by caliper measurements and expressed as the average of two perpendicular diameters. Lack of survival was defined as mouse death or mean tumor diameter size of 20 mm.

### METHOD DETAILS

#### Tumor rechallenge

For tumor rechallenge, mice that rejected primary tumors after treatment with anti-CTLA-4, anti-PD-1, anti-CTLA-4 + anti-PD-1, or neoAg SLP vaccines were then rechallenged with same number of cells used in primary challenge with either the same tumor line used in the primary tumor challenge, or a different tumor line as indicated at least 60 days after complete rejection of the primary tumor.

#### *In vivo* antibody treatments

For ICT treatment, YUMM1.7 parental, Y1.7LI, or Y1.7AI tumor-bearing mice were treated intraperitoneally with 200 μg of anti-CTLA-4 and/or anti-PD-1 on day 3, 6, 9, 12, 18, and 22 or day 7, 10, 13, 16, 22, and 28; or day 12, 15, 18, 21, 27 and 33 post-tumor transplant. For control groups, mice were injected with 200 μg of IgG2a isotype control antibodies. MC38 tumor-bearing mice were treated intraperitoneally with 200 μg of anti-CTLA-4 and/or anti-PD-1 on day 12, 15, 18, and 22 post-transplant. For anti-TREM2, mice were treated via intraperitoneal injection on day 7, 12, and 17, 22 and 27 post-tumor transplant with 200 μg of anti-TREM2 or relevant isotype control mAb. Anti-mouse TREM2 [Fc mutated (Clone 178 (LALAPG)) was used for non-depleting, TREM2 blocking. For antibody depletion studies, 250 μg of control mAb, anti-CD4, or anti-CD8α was injected intraperitoneally into mice at day —1 and every 7 days thereafter until day 20. CD4 and CD8 depletion was verified by flow cytometry analysis of surface-stained peripheral blood monocytes (PBMC) and intratumoral immune cells. For *in vivo* experiments, “*In vivo* Platinum”-grade antibodies that were verified to be free of mouse pathogens (IDEXX IMPACT I mouse pathogen testing) were purchased from Leinco Technologies: anti-PD-1 (rat IgG2a clone RMP1-14), anti-CTLA-4 (murine IgG2b clone 9D9), anti-CD4 (rat IgG2b clone GK1.5), anti-CD8α (rat IgG2b clone YTS-169), anti-TREM2 (Fc Muted Clone 178), and isotype controls [rat IgG2a clone 1-1, mouse IgG2a clone C1.18.4, or anti-Human ILT1 (Fc Muted Clone 135.5; Isotype Control for anti-TREM2)].

#### Peptides

Mutant Lama4 8-mer (VGFNFRTL), mutant Lama4 SLP (QKISFFDGFEVGFNFRTLQPNGLLFYYT), mutant Adpgk SLP (HLELASMTN MELMSSIVHQ), mutant Rpl18 SLP (KAGGKILTFDRLALESPK), mutant Dpagt1 SLP (EAGQSLVISASIIVFNLLELEGDYR), mutant Alg8 8-mer (ITYTWTRL), mutant Alg8 SLP (AVGITYTWTRLYASVLTGSLV), and mutant Itgb1 SLP (DDCWFYFTYSVNGYNEAIVHV VETPDCP) peptides were custom ordered from Peptide 2.0. All peptides were HPLC purified to >95% purity.

#### Vaccination

Y1.7LI or Y1.7AI tumor bearing male mice were vaccinated subcutaneously with 10 μg of mLama4 or mAlg8 synthetic long peptide (SLP) in combination with 50 μg of VacciGrade^™^ high molecular weight Polyinosinic-polycytidylic acid (pI:C) (InvivoGen) diluted in endotoxin-free sterile PBS to a total volume of 150 μL on day 3, 9, and 15 post-tumor transplant. In separate experiments, Y1.7LI tumor bearing male mice were vaccinated subcutaneously with 10 μg of mLama4 or mAlg8 SLP in combination with 50 μg of pI:C (diluted with endotoxin-free sterile PBS to a total volume of 150 μL) on day 7, 13, and 19 or day 12, 18, and 24 post-tumor transplant. MC38 tumor bearing female mice were vaccinated subcutaneously with 20 μg of mAdpgk SLP plus 20 μg of mRpl18 SLP plus 20 μg of mDpagt1 plus 50 μg pI:C adjuvant or control vaccine composed of 40 μg of irrelevant human papillomavirus (HPV) SLP + 50 μg of pI:C on day 12 and 19 post-tumor transplant. For SLP, peptide sequence used for mLama4; QKISFFDGFEVGFNFRTLQPNGLLFYYT, for mAlg8; AVGITYTWTRLYASVLTGSLV, for mAdpgk; HLELASMTNMELMSSIVHQ, for mRpl18; KAGGKILTFDRLALESPK and for mDpagt1; EAGQSLVISASIIVFNLLELEGDYR. mLama4 SLP served as a relevant SLP for the Y1.7LI line and an irrelevant SLP for the Y1.7AI line. mAlg8 served as a relevant SLP for the Y1.7AI line and an irrelevant SLP for the Y1.7LI tumor.

#### Tumor and spleen harvest

Established tumors were excised from mice, minced, and treated with 1 mg/mL type IA collagenase (Sigma-Aldrich) in HBSS (Hyclone) for 45 minutes at 3°C. Cells were washed thrice. Red blood cells were lysed using ACK lysis buffer (Gibco). To remove aggregates and clumps, cells were passed through a 40-μm strainer. Spleens were harvested, crushed, and vigorously resuspended to make single-cell suspensions. To remove aggregates and clumps, cells were passed through a 70-μm strainer and subsequently through a 40-μm strainer.

#### Tetramer staining

For tetramer staining, cells were incubated for 5 min at room temperature with 500 ng of rat anti-mouse anti-CD16/32 (mouse BD Fc Block; clone 2.4G2, BD Biosciences) at 1 μg/million cells. Peptide-H-2K^b^ tetramers conjugated to PE (1:50) or APC (1:25) for mutated Alg8, mutated Lama4, or OVA-I (SIINFEKL; irrelevant control tetramer) were added to cells and incubated for 20 min at 37°C. Tetramer-stained cells were further stained with an antibody master mix consisting of anti-CD45, anti-CD90.2/Thy1.2, anti-CD8α, anti-CD4, anti-PD-1, anti-TIM-3, and anti-LAG-3 antibodies and live/dead dye (NIR) in 100 μl FACS buffer (PBS with 2% FBS, 2 mmol/L EDTA, and 0.05% NaN3; Sigma) for 20 min at 4 °C. OVA-I (SIINFEKL)-H-2K^b^, mutant Alg8-H-2K^b^, and mutant Lama4-H-2K^b^ tetramers conjugated to PE or APC fluorophores, were obtained from the Baylor College of Medicine MHC Tetramer Production Facility.

#### Dextramer staining

For dextramer staining, cells were incubated with Fc block as described above (see tetramer staining section). Peptide-H-2K^b^ dextramer conjugated to PE for mutated Lama4 was added to cells at a 1:10 dilution and incubated for 20 min at 37°C. Dextramer-stained cells were further stained with an antibody master mix consisting of anti-CD45, anti-CD90.2/Thy1.2 and anti-CD8α antibodies and live/dead dye (NIR) in 100 μl FACS buffer for 20 min at 4 °C. Mutant Lama4-H-2K^b^ dextramer conjugated to PE fluorophore was obtained from Immudex LLC, Fairfax, VA, USA.

#### Apoptosis assay

For apoptosis assay, cells were incubated with Fc Block as described above (see tetramer staining section) and then surface stained with an antibody master mix consisting of anti-CD45, anti-CD90.2/Thy1.2 and anti-CD8a antibodies and live/dead dye (NIR) in 100 μl FACS buffer for 20 min at 4 °C. Surface stained cells were washed twice with cold BioLegend’s Cell Staining Buffer, and then resuspended in Annexin V Binding Buffer (BioLegend) at a concentration of 0.25–1.0 × 10^7^ cells/ml. Cell suspensions were stained with 5 ml of Annexin V-FITC (BioLegend) Staining Solution, vortexed, and incubated for 15 min at 25°C in the dark. Next, 400 mL of Annexin V Binding Buffer was added to each tube and analyzed by flow cytometry.

#### Flow cytometry

For flow cytometry, cells were incubated with Fc Block as described above (see tetramer staining section) and then stained with surface flow antibodies for 20 minutes at 4°C. Surface antibodies were diluted in FACS staining buffer. Anti-mouse CD45-BV605, CD90.2/Thy1.2-PE-Cy7, CD8α-BV786, CD4-BV711, CD19-BV650, CD20-BV421, CD45R/B220-BUV395, Nkp46/CD335-FITC, γδ TCR-PE-Cy7, PD-1-BV421, TIM-3-BV711, LAG-3-PerCP-Cy5.5, CD3ε-APC, CD64-BV421, Ly6G-Alexa Fluor 700, CX3CR1-FITC, I-A/I-E-BV650, CD103-BV421, CD24-BV711, CD11c-BV786, CD11b-APC, F4/80-BUV395, CD64-APC, CD117-FITC, CD11b-PerCP-Cy5.5, PDCA-1/BST-2-BV650, CD172a-APC, PD-L1-PE, and FcεRI-PE-Cy7 were used for surface staining at the indicated dilutions. Zombie NIR Viability dye was added at 1:500 during surface staining.

For intracellular staining to detect CD206 and iNOS, surface-stained cells were fixed and permeabilized with Fixation/Permeabilization Solution Kit (BD Bioscience). Fixed and permeabilized cells were then stained with anti-mouse CD206-PE-Cy7 and anti-mouse iNOS-PE for 30 minutes at 4°C. The exception was for sorting of live CX3CR1^+^ CD206^+^ macrophages used for intratumoral injection (Figure 7C), where anti-CD206 was instead included in surface staining and intracellular staining was not performed.

For intracellular cytokine staining of lymphocytes, cells from tumors, isolated as described above (see tumor and spleen harvest section), were stained, and CD4 and CD8 T cells were sorted. For sorting CD4 and CD8 T cells, tumor cells were stained with Fc block as described above (see tetramer staining section) for 5 min at room temperature and then stained with antibodies to CD45, CD3ε, CD4 or CD8α and Zombie NIR Viability dye in 100 μl of staining buffer. Cells were incubated for 30 minutes at 4°C. Live CD45^+^Cd3ε^+^CD4^+^ and live CD45^+^Cd3ε^+^CD8α^+^ were then sorted on a BD FACSAria II (BD Biosciences). 100,000 splenocytes harvested from naïve mice were then pulsed with 1 mM of mLama4 8-mer peptide or mItgb1 28-mer peptide and 100,000 CD8 or CD4 T cells were subsequently added and incubated at 37 °C. After 1 h, BD GolgiPlug (BD Bioscience) was added in, and cells were incubated for an additional 5 h at 37 °C. Cells were then washed and stained for 5 minutes at room temperature with Fc block at 1 mg/million cells and then surface stained for 30 minutes at 4°C, and then fixed and permeabilized with BD Fixation and Permeabilization Kit. Fixed and permeabilized cells were then stained with anti-mouse IFN-γ-APC and anti-mouse TNF-PE-Cy7 for 30 minutes at 4°C.

For FOXP3 staining, surface-stained cells were fixed and permeabilized using the eBioscience FOXP3/Transcription Factor Staining Buffer Set. Fixed and permeabilized cells were then stained with anti-mouse FOXP3-FITC for 30 minutes at 4°C. All flow cytometry was performed on an BD Fortessa X-20, or BD LSR II, and analyzed using FlowJo software. Gating strategy used is depicted in Figure S12.

#### Quantitative RT-PCR

RNA was extracted from sorted macrophages using RNAeasy Plus Mini Kit (Qiagen). 100 μg of RNA was reverse-transcribed and subjected to qRT-PCR using the SuperScript III Platinum Two-Step qRT-PCR Kit with SYBR Green (Invitrogen). qPCR was performed on the StepOne Real-Time PCR System (Applied Biosystems). Each sample was run in triplicate for each gene and the cDNA from each sample was divided equally per reaction in a 20 μl volume. The qPCR conditions were as follows: 50°C for 2 minutes and 95°C for 2 minutes, followed by 40 cycles of 95°C for 15 seconds and 59°C for 30 seconds. Melting curve analysis was performed to confirm a single amplicon. Differences in gene expression were determined using the equation 2-ΔΔCt, where the Ct value of *Trem2* was subtracted from the Ct value of the *Gapdh* control to yield the DCt value. For each sample, the ΔCt value *Trem2* done in triplicate was averaged and compared to give one ΔΔCt value per sample. Mouse qPCR *Trem2* primers were as follows: forward primer-5’ CTGGAACCGTCACCATCACTC3’ and reverse primer-5’CGAAACTCGATGACTCCTCGG3’. Mouse qPCR *Gapdh* primers were as follows: forward primer-5’AGGTCGGTGTGAACGGATTTG3’ and reverse primer-5’TGTAGACCATGTAGTTGAGGTCA3’.

#### scRNAseq

For scRNAseq profiling of intratumoral live CD45^+^ cells, tumors from 5 individual mice per treatment group were pooled and processed and for neoAg-specific CD8 T cells, tumors from 5 individual mice per treatment group were pooled and processed.

#### Antibody hashing for multiplexing

Antibody hashing and multiplexing was utilized for scRNAseq of neoAg-specific CD8 T cells. For the CD45^+^ scRNAseq experiment, antibody hashing and multiplexing was not performed. For the neoAg-specific CD8 T cells scRNAseq experiment, cell labeling was performed according to an adapted BioLegend cell hashing protocol (TotalSeq^™^-C Antibodies and Cell Hashing with 10x Single Cell 5’ Reagent Kit v1.1 Protocol, BioLegend). Single cell suspensions of harvested tumors from treated mice were resuspended in BioLegend Cell Staining Buffer containing Fc block and stained with mLama4 PE and APC labelled tetramers as described above (see tetramer staining section). Tetramer-stained cells from control mAb, Control Vax, and neoAg SLP Vax treatment conditions were immediately surface stained with an antibody master mix consisting of anti-CD90.2/Thy1.2-PE-Cy7, anti-CD8α-BV786 antibodies and live/dead dye (NIR) and incubated for 20 min at 4°C. Tetramer-stained samples from anti-CTLA-4-, anti-PD-1-, and anti-CTLA-4 plus anti-PD-1-treated groups were incubated with mixture of surface stained (antibody master mix consisting of anti-CD90.2/Thy1.2-PE-Cy7, anti-CD8α-BV786 antibodies and live/dead dye (NIR)) and barcoded antibodies with unique hashtags for each treatment condition [anti-CTLA-4: Hashtag 1 Total Seq^™^-C0301 anti-mouse Hashtag 1 Antibody; anti-PD-1: Hashtag 2 (Total Seq^™^-C0302 anti-mouse Hashtag 2 Antibody); anti-CTLA-4 + anti-PD-1 combination: Hashtag 3 (Total Seq^™^-C0303 anti-mouse Hashtag 3 Antibody)] for 30 min at 4°C. Hashtag antibodies were used at a concentration of 1 μg per 2 million cells. Cells were then washed 3X with BioLegend Cell Staining Buffer. mLama4 tetramer-specific CD8 T cells from each treatment condition were sorted on a BD FACSAria II. Sorted mLama4 tetramer-specific CD8 T cells in anti-CTLA-4, anti-PD-1, and anti-CTLA-4 + anti-PD-1 treated samples with unique hashtags were pooled for single-cell library generation and CITE-seq (cellular indexing of transcriptomes and epitopes by sequencing) through multiplexing. Separate libraries were generated for control mAb, Control Vax, and neoAg SLP Vax samples and, thus, these were not multiplexed. Cells were counted on a Countess 3 FL automated cell counter (Life Technologies) and viabilities were determined using trypan blue exclusion assay. Cell capture processing and gene expression and feature barcode library preparations were performed following 10X Genomics’ guidelines for 5’ scRNAseq [CG000330_Chromium Next GEM Single Cell 5’ v2 (Dual Index) with Feature Barcode technology-Rev F]. QC steps after cDNA amplification and library preparation steps were carried out by running ThermoFisher Qubit HS dsDNA Assay along with Agilent (Santa Clara, CA) HS DNA Bioanalyzer for concentration and quality assessments, respectively. Library sample concentrations were verified using qPCR using a KAPA Biosystems KAPA Library Quantification Kit prior to pooling. Libraries were normalized to 5 nM for pooling. The pool was sequenced using a NovaSeq6000 S4-XP, 200-cycle flow cell lane. The run parameters used were 26 cycles for read 1, 90 cycles for read2, 10 cycles for index1, and 10 cycles for index2 as stipulated in the protocol mentioned above. Raw sequencing data (fastq file) was demultiplexed and analyzed using 10X Genomics Cell Ranger v.7.1.0 software utilizing standard default settings and the cellranger count command to generate html QC metrics and coupé/vloupe files for each sample. We profiled between 937 to 1762 mLama4 tetramer-specific CD8 T cells for each of the different ICT treatment conditions and 4459, 6723, and 7646 mLama4-specific CD8 T cells for control mAb, Control Vax, and neoAg SLP Vax, respectively.

#### CD45^+^ scRNAseq library generation

Droplet-based 5′ end massively parallel scRNAseq was performed by encapsulating sorted live CD45^+^ tumor-infiltrating cells into droplets and libraries were prepared using Chromium Next GEM Single-cell 5′ Reagent Kit v2 (10x Genomics) according to manufacturer’s protocol. The generated scRNAseq libraries were sequenced using an Illumina NovaSeq6000 S2 flow cell.

#### scRNAseq alignment, barcode assignment, and unique molecular identifier counting

The Cell Ranger Single-Cell Software Suite available at https://support.10xgenomics.com/single-cell-gene-expression/software/overview/welcome was used to perform sample demultiplexing, barcode processing, and single-cell 5′ counting. Cellranger mkfastq was used to demultiplex raw base call files from the NovaSeq6000 sequencer, into sample-specific fastq files. Files were demultiplexed with 81.9% to 97.1% perfect barcode match, and 90%+ q30 reads. Afterward, fastq files for each sample were processed with Cellranger count, which was used to align samples to mm10 genome, filtered, and quantified. For each sample, the recovered cells’ parameter was specified as 10,000 cells that we expected to recover for each individual library.

#### Preprocessing analysis with Seurat package

The Seurat pipeline was applied to each dataset following tutorial specifications from https://satijalab.org/seurat/articles/archive; version 4.3 and https://hbctraining.github.io/scRNA-seq_online/. Data from all groups were merged into a single Seurat object, and integration was performed using the reciprocal principal component analysis (PCA) workflow to identify integration anchors. After integration, genes that were expressed in fewer than 3 cells and cells that contained fewer than 500 transcripts (unique molecular identifiers; UMI) were excluded. Cells with more than 10% of mitochondrial transcripts were also excluded from analysis. The cutoffs used were set based on the characteristics of the cell population in each dataset. Data were normalized using LogNormalize method (counts for each cell divided by the total counts for that cell, multiplied by the scale factor of 10^4^ and natural-log transformed using log1p). PCA was performed on about 2,000 genes with PCA function. A uniform manifold approximation and projection (UMAP) dimensional reduction was performed on the scaled matrix (with most variable genes only) using the first 40 or 50 principal components (PCA) for mLama4 tetramer-specific CD8 T cells and CD45^+^ cells, respectively, to obtain a two-dimensional representation of the cell states. For clustering, we used the function FindClusters that implements SNN (shared nearest neighbor) modularity optimization–based clustering algorithm on 30 PCA components, leading to 33 clusters.

#### Identification of cluster-specific genes and marker-based classification

To identify marker genes, the FindAllMarkers function was used with likelihood-ratio test for single-cell gene expression. To characterize clusters, we used ImmGen database. For heat map representation, mean expression of markers inside each cluster was used. To compare gene expression for the clusters inside cohorts (e.g., T cells, macrophages) we used FindMarkers function to calculate average log2 fold change and identify differentially expressed genes between each pair of experimental conditions using a Wilcoxon rank-sum test for calculating P values and Bonferroni correction for Padj values.

#### T cell population analysis

To gain more insights into different immunotherapies-induced T cells remodeling in the TME, we subclustered activated T cells (excluding quiescent T cell clusters 10 and 12). Identification of most variable genes, PCA, UMAP, clustering, and marker selection analysis were performed as described above.

#### Gene set enrichment analysis (GSEA)

To identify if MSigDB hallmark gene sets are up-regulated or down-regulated between clusters and treatments, we performed gene set enrichment analysis. Fold-changes of gene expression between comparisons were calculated using Seurat R package v.4.3.0.1, and normalized enrichment scores as well as p-values of given gene sets were then estimated using the gage R package v.2.46.1.

#### Pseudotime trajectory analysis

To determine the potential lineage differentiation within CD4 T cell subpopulations, we used the Monocle3 R package to construct CD4 differentiation trajectories after specifying the corresponding cells as root nodes. Subsequently, graph test was used to find the pseudotime trajectory difference genes, and the obtained genes were used to plot the heat map. The origin of the inferred pseudotime was assigned with pseudotime score 0, and geodesic distances and pseudotime score among other CD4 T cells are calculated from there based on transcripts associated cell states.

#### Comparison to published datasets

The gene lists defining phenotype of intratumoral T cells were retrieved from published human or mouse scRNAseq datasets^31,41,59–61,67^, (Table S2) to compare the cluster annotation between published study and our current study. The module scores for individual cells were calculated using the “AddModuleScore” function in the Seurat package. The results were visualized using the normalized average module scores for each cluster (Figures 4A, 4B, and 5C).

### QUANTIFICATION AND STATISTICAL ANALYSIS

Samples were compared using an unpaired, two-tailed Student t test, two-way ANOVA, or log-rank (Mantel–Cox) test unless specified otherwise.

## Supplementary Material

1

2

3

## Figures and Tables

**KEY RESOURCES TABLE:** 

REAGENT or RESOURCE	SOURCE	IDENTIFIER

Antibodies		

Anti-mouse CTLA-4 (clone 9D9)	Leinco Technologies	Cat# C2856; RRID: AB_2829611
Anti-mouse CD279 (PD-1) (clone RMP1-14)	Leinco Technologies	Cat# P372; RRID: AB_2749820
Anti-mouse CD8 (clone YTS-169)	Leinco Technologies	Cat# C2442; RRID: AB_2829540
Anti-mouse CD4 (clone GK1.5)	Leinco Technologies	Cat# C2838; RRID: AB_2829596
Anti-Mouse TREM2 - Fc Muted [clone 178 (LALAPG)]	Leinco Technologies	Cat# T721
Anti-mouse IgG2a Isotype Control (clone C1.18.4)	Leinco Technologies	Cat# P381; RRID: AB_2831654
Rat IgG2a Isotype Control (clone 1-1)	Leinco Technologies	Cat# R1367; RRID:AB_2831721
Anti-Human ILT1, Isotype Control, Fc Muted (clone 135.5 (clone LALAPG))	Leinco Technologies	Cat# I-1241
Anti-CD16/32 (clone 2.4G2)	BD Biosciences	Cat# 553141; RRID: AB_394656
Anti-mouse CD45 BV605 (clone 30-F11) (1:800 dilution)	BioLegend	Cat# 103140; RRID: AB_2562342
Anti-mouse CD90.2/Thy1.2-PE-Cy7 (clone 30-H12) (1:500 dilution)	BioLegend	Cat# 105326; RRID: AB_2201290
Anti-mouse CD8a-BV786 (clone 53-6.7) (1:200 dilution)	BD Bioscience	Cat# 563332; RRID: AB_2721167
Anti-mouse CD4-BV711(clone RM4-5) (1:200 dilution)	BioLegend	Cat#100550; RRID: AB_2562099
Anti-mouse CD19-BV650 (clone 1D3) (1:200 dilution)	BD Bioscience	Cat# 563235; RRID: AB_2738085
Anti-mouse CD20-BV421 (clone SA275A11) (1:200 dilution)	BioLegend	Cat# 150405; RRID: AB_2566540
Anti-mouse CD45R/B220-BUV395 (clone RA3-6B2) (1:200 dilution)	BD Bioscience	Cat# 563793; RRID: AB_2738427
Anti-mouse Nkp46/CD335-FITC (clone 29A1.4) (1:300 dilution)	BioLegend	Cat# 560756; RRID: AB_1727465
Anti-mouse γδ TCR-PE-Cy7 (clone GL3) (1:300 dilution)	BioLegend	Cat# 118124; RRID: AB_11204423
Anti-mouse PD-1-BV421 (clone 29F.1A12) (1:200 dilution)	BioLegend	Cat# 135218; RRID: AB_2561447
Anti-mouse TIM-3-BV711 (clone RMT3-23) (1:200 dilution)	BioLegend	Cat# 119727; RRID: AB_2716208
Anti-mouse LAG-3-PerCP-Cy5.5 (clone C9B7W) (1:200 dilution)	BioLegend	Cat# 125212; RRID: AB_2561517
Anti-mouse CD3e-APC (clone 145-2C11) (1:200 dilution)	BioLegend	Cat# 100312; RRID: AB_312677
Anti-mouse CD64-BV421 (clone X54-5/7.1) (1:200 dilution)	BioLegend	Cat# 139309; RRID: AB_2562694
Anti-mouse Ly6G-Alexa Fluor 700 (clone 1A8) (1:400 dilution)	BD Biosciences	Cat# 127622; RRID: AB_10643269
Anti-mouse CX3CR1-FITC (clone SA011F11) (1:1,000 dilution)	BioLegend	Cat# 149020; RRID: AB_2565703
Anti-mouse I-A/I-E-BV650 (clone M5/114.15.2) (1:3,000 dilution)	BD Bioscience	Cat# 563415; RRID: AB_2738192
Anti-mouse CD103-BV421 (clone 2E7) (1:200 dilution)	BioLegend	Cat#121422; RRID: AB_2562901
Anti-mouse CD24-BV711 (clone M1/69) (1:1000 dilution)	BD Bioscience	Cat# 563450; RRID: AB_2738213
Anti-mouse CD11c-BV786 (clone HL3) (1:400 dilution)	BD Biosciences	Cat# 563735; RRID: AB_2738394
Anti-mouse CD11b-APC (clone M1/70) (1:400 dilution)	BioLegend	Cat# 101212; RRID: AB_312795
Anti-mouse F4/80-BUV395 (clone T45-2342) (1:400 dilution)	BD Biosciences	Cat# 565614; RRID: AB_2739304
Anti-mouse CD64-APC (clone X54-5/7.1) (1:400 dilution)	BioLegend	Cat# 139306; RRID: AB_11219391
Anti-mouse CD117-FITC (clone ACK2) (1:100 dilution)	BioLegend	Cat# 135115; RRID: AB_2561633
Anti-mouse CD11b-PerCP-Cy5.5 (clone M1/70) (1:200 dilution)	BioLegend	Cat# 561114; RRID: AB_394002
Anti-mouse PDCA-1/BST-2 BV650 (clone 927) (1:200 dilution)	BD Biosciences	Cat# 747605; RRID: AB_2744173
Anti-mouse CD172a APC (clone P84) (1:200 dilution)	BioLegend	Cat# 144014; RRID: AB_2564061
Anti-mouse CD274/PDL1-PE (clone MIH5) (1:200 dilution)	BD Biosciences	Cat# 558091; RRID: AB_397018
Anti-mouse FcεRI-PE-Cy7 (clone MAR-1) (1:200 dilution)	BioLegend	Cat# 134326; RRID: AB_2572064
Anti-mouse Mrc1 (CD206)-PE-Cy7 (clone C068C2) (1:400 dilution)	BioLegend	Cat# 141720; RRID: AB_2562248
Anti-mouse FOXP3-FITC (clone FJK-16s)	eBioscience^™^	Cat# 11-5773-82; RRID: AB_465243
Anti-mouse IFNγ-APC (clone XMG1.2) (1:200 dilution)	BD Biosciences	Cat#554413; RRID: AB_398551
Anti-mouse TNF-PE-Cy7 (clone MP6-XT22) (1:200 dilution)	BD Biosciences	Cat# 561062; RRID: AB_398553
Anti-mouse Granzyme B-PE (clone NGZB) (1:200 dilution)	eBioscience^™^	Cat# 12-8898-82; RRID: AB_10870787
Anti-mouse iNOS/Nos2 PE (clone CXNFT) (1:200 dilution)	eBioscience^™^	Cat# 12-5920-82; RRID: AB_2572642
CD45 and H-2 MHC class Totalseq^™^-C0301 anti-mouse Hashtag 1, Antibody	BioLegend	Cat# 155861; RRID: AB_2800693
CD45 and H-2 MHC class Totalseq^™^-C0302 anti-mouse Hashtag 2 Antibody	BioLegend	Cat# 155863; RRID: AB_2800694
CD45 and H-2 MHC class Totalseq^™^-C0303 anti-mouse Hashtag 3 Antibody	BioLegend	Cat# 155865; RRID: AB_2800695

Bacterial and virus strains		

pMSCV-IRES GFP	Addgene	Cat# 20672

Chemicals, peptides, and recombinant proteins		

RPMI-1640	HyClone	Cat# SH30096.02
Trypsin	Genclone	Cat# 25200056
Defined fetal bovine serum	HyClone	Cat# SH30070.03HI
HBSS	Hyclone	Cat# SH30588.02
PBS	Gibco	Cat# 20012027
Sodium Bicarbonate	Gibco	Cat# 25080094
Sodium Pyruvate	Gibco	Cat# 11360070
L-Glutamine	Gibco	Cat# A2916801
ACK lysis buffer	Gibco	Cat# A1049201
Phorbol-12-myristate-13-acetate (PMA)	MilliporeSigma	Cat# 500582
Ionomycin	Thermo Fisher Scientific	Cat# BP2527-1
Fugene	Promega	Cat# E2311
Collagenase Type IA	Sigma-Aldrich	Cat# C9891
Zombie Fixable Viability^™^ Sampler Kit	BioLegend	Cat# 423105
Trypan Blue	Gibco	Cat# 15250061
Poly(I:C) HMW VacciGrade^™^	InvivoGen	Cat# vac-pic
Gibson Assembly^®^ Cloning Kit	New England Biolabs	Cat# E5510S
BglII	New England Biolabs	Cat# R0144S
Mutant Lama4 peptide, sequence ***V***GFNFRTL	Gubin et al.,^16^ Peptide 2.0	N/A
Mutant Alg8 peptide, sequence ITY***T***WTRL	Gubin et al.,^16^ Peptide 2.0	N/A
Mutant Alg8 peptide, sequence AVGITY***T***WTRLYASVLTGSLV	Gubin et al.,^16^ Peptide 2.0	N/A
Mutant Lama4 SLP, sequence QKISFFDGFE***V***GFNFRTLQPNGLLFYYT	Gubin et al.,^16^ Peptide 2.0	N/A
Mutant Adpgk SLP, sequence HLELASMTN***M***ELMSSIVHQ	Yadav et al.,^20^ Peptide 2.0	N/A
Mutant Rpl18 SLP, sequence KAGGKILTFD***R***LALESPK	Hos et al.,^80^ Peptide 2.0	N/A
Mutant Dpagt1 SLP, sequence EAGQSLVISASIIVFNL***L***ELEGDYR	Yadav et al.,^20^ Peptide 2.0	N/A
Mutant Itgb1 SLP, sequence DDCWFYFTYSVNGY***N***EAIVHVVETPDCP	Alspach et al.,^23^ Peptide 2.0	N/A

Critical commercial assays		

RNAeasy Plus Mini Kit	Qiagen	Cat# 74134
SuperScript III Platinum Two-Step qRT-PCR Kit with SYBR Green	Invitrogen	Cat# 11735-032
Chromium Next GEM Single-cell 5'Reagent Kit v2	10x Genomics	Cat# PN-1000263
Fixation/Permeabilization Solution Kit	BD Biosciences	Cat# 555028; RRID: AB_2869013
Foxp3 / Transcription Factor Staining Buffer Set	eBioscience	Cat# 00-5523-00
Cell Staining Buffer	BioLegend	Cat# 420201
FITC Annexin V Apoptosis Detection Kit with 7-AAD	BioLegend	Cat# 640922
Chromium Next GEM Single Cell 5' Reagent Kit v2	10x Genomics	Cat# 100263
Chromium Next GEM Single Cell 5' v2 (Dual Index)	10x Genomics	Cat# CG000330
Qubit HS dsDNA Assay	ThermoFisher	Cat# Q32851
KAPA Biosystems KAPA Library Quantification Kit	Roche	Cat# 07960140001

Deposited data		

Raw and analyzed data	This paper	GEO: GSE276902 GEO: GSE276904

Experimental models: cell lines		

Phoenix Eco cells	Alspach et al.^23^	N/A
Braf^V600E^ Cdkn2a^−/−^ Pten^−/−^ YUMM1.7 parental line	ATCC	CRL-3362
Y1.7LI line	This paper	N/A
Y1.7AI line	This paper	N/A
MC38	Noguchi et al.^114^	N/A

Experimental models: organisms/strains		

Mouse: C57BL/6J	The Jackson Laboratory	Strain #:000664; RRID: IMSR_JAX:000664

Oligonucleotides		

mAlg8-P2A-mItgb1 CTTCTCTAGGCGCCGGAATTCAG CCACCATGGCAGTGGGCATCACA TACACCTGGACCAGGCTGTATGC TTCAGTGTTGACTGGCTCCCTTG TCGGCAGCGGCGCCACAAACTTC TCTCTGCTAAAGCAAGCAGGTGA TGTTGAAGAAAACCCCGGGCCTG ATGACTGCTGGTTCTATTTCACCT ATTCAGTGAATGGCTACAATGAAG CTATCGTGCATGTTGTGGAGACTC CAGACTGTCCTTAATACGTAGCTA GCGGATCCCA	This paper; IDT	N/A
mLama4-P2A-mItgb1 CTTCTCTAGGCGCCGGAATTCAGCCA CCATGCAGAAAATATCTTTCTTTGATGGCT TTGAAGTAGGCTTCAATTTCCGAACATTAC AGCCAAATGGGTTACTATTCTACTACACA GGCAGCGGCGCCACAAACTTCTCTCTGCT AAAGCAAGCAGGTGATGTTGAAGAAAACC CCGGGCCTGATGACTGCTGGTTCTATTTC ACCTATTCAGTGAATGGCTACAATGAAGC TATCGTGCATGTTGTGGAGACTCCAGACT GTCCTTAATACGTAGCTAGCGGATCCCA	This paper; IDT	N/A
Mouse qPCR Trem2 Forward primer-5' CTGGAACCGTCACCATCACTC3'	Ishida-Kitagawa et al.^115^; IDT	N/A
Mouse qPCR Trem2 Reverse primer-5'CGAAACTCGATGACTCCTCGG3'	Ishida-Kitagawa et al.^115^; IDT	N/A
Mouse qPCR Gapdh Forward primer-5'AGGTCGGTGTGAACGGATTTG3'	Dasetal.^116^; IDT	N/A
Mouse qPCR Gapdh Reverse primer-5'TGTAGACCATGTAGTTGAGGTCA3'	Dasetal.^116^; IDT	N/A

Software and algorithms		

Flow jo_v10.8.1	BD	https://www.flowjo.com/bd-flowjo-faq
Prism version 10	GraphPad	https://www.graphpad.com/
Cell Ranger v.7.1.0	https://support.10xgenomics.com/single-cell-gene-expression/software	N/A
Seurat R package v.4.3.0.1	https://satijalab.org/seurat/	N/A
gage R package v.2.46.1	https://github.com/datapplab/gage/blob/master/NEWS	N/A
ggplot2	https://ggplot2.tidyverse.org/index.html	N/A
Monocle3 R package	https://cole-trapnell-lab.github.io/monocle3/	N/A

Other		

Fortessa X-20	BD Biosciences	N/A
LSR II	BD Biosciences	N/A
BD FACSAria II	BD Biosciences	N/A
StepOne Real-Time PCR System	Applied Biosystems	N/A
HS DNA Bioanalyzer	Agilent	N/A
Countess^™^ 3 FL Automated Cell Counter	Invitrogen	Cat# A49866
NovaSeq6000 S2	Illumina	N/A
NovaSeq6000 S4-XP	Illumina	N/A

## Highlights

- NeoAg vaccines utilize mechanisms partially distinct from those of αCTLA-4 or αPD-1
- NeoAg vaccines potently induce TCF-1^+^ stem-like and proliferating neoAg-specific CD8 T cells
- NeoAg vaccines induce macrophage remodeling partially distinct from that of αCTLA-4 or αPD-1
- TREM2 blockade remodels intratumoral macrophages and enhances neoAg vaccine efficacy
