# Supplementary material for: Comparing neoantigen cancer vaccines and immune checkpoint therapy unveils an effective vaccine and anti-TREM2 macrophage-targeting dual therapy
Source: Cell Rep. Author manuscript; Available in PMC 2025 Jan 31. (PMC11785356; doi:10.1016/j.celrep.2024.114875)
Supplement: 1 [file NIHMS2038994-supplement-1.pdf]

Cell Reports, Volume *43*

Supplemental information

Comparing neoantigen cancer vaccines and immune checkpoint therapy unveils an effective vaccine and anti-TREM2 macrophage-targeting dual therapy

Sunita Keshari, Alexander S. Shavkunov, Qi Miao, Akata Saha, Tomoyuki Minowa, Martina Molgora, Charmelle D. Williams, Mehdi Chaib, Anna M. Highsmith, Josué E. Pineda, Sayan Alekseev, Elise Alspach, Kenneth H. Hu, Marco Colonna, Kristen E. Pauken, Ken Chen, and Matthew M. Gubin

Figure S1

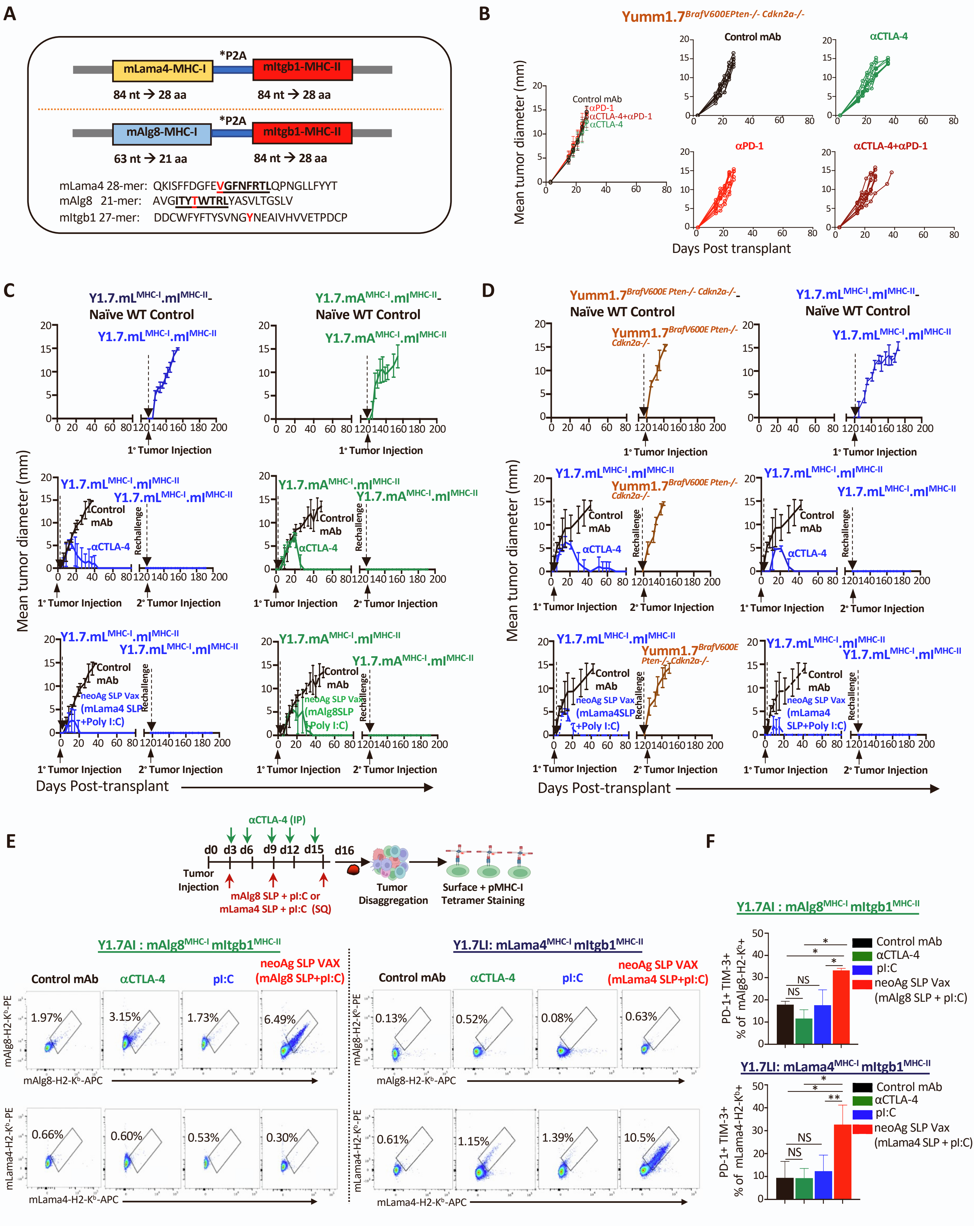
A
mLama4-MHC-I
*P2A
mItgb1-MHC-II
84 nt → 28 aa
84 nt → 28 aa
mAlg8-MHC-I
*P2A
mItgb1-MHC-II
63 nt → 21 aa
84 nt → 28 aa
mLama4 28-mer: QKISFFDGFEVGFNFRTLQPNGLLFYYT
mAlg8 21-mer: AVGITYTWTRLYASVLTGSLV
mItgb1 27-mer: DDCWFYFTYSVNGYNEAIVHVVETPDCP
B
Yumm1.7BrafV600EPten-/- Cdkn2a-/-
Control mAb
αCTLA-4
αPD-1
αCTLA-4+αPD-1
Mean tumor diameter (mm)
Days Post transplant
C
Y1.7.mLMHC-I.mIMHC-II-Naïve WT Control
Y1.7.mAMHC-I.mIMHC-II-Naïve WT Control
1° Tumor Injection
2° Tumor Injection
Rechallenge
Control mAb
αCTLA-4
neoAg SLP Vax (mLama4 SLP+Poly I:C)
neoAg SLP Vax (mAlg8SLP+Poly I:C)
Mean tumor diameter (mm)
Days Post-transplant
D
Yumm1.7BrafV600E Pten-/- Cdkn2a-/--Naïve WT Control
Y1.7.mLMHC-I.mIMHC-II-Naïve WT Control
neoAg SLP Vax (mLama4SLP+Poly I:C)
neoAg SLP Vax (mLama4 SLP+Poly I:C)
Mean tumor diameter (mm)
Days Post-transplant
E
αCTLA-4 (IP)
d0 d3 d6 d9 d12 d15 d16
Tumor Injection
mAlg8 SLP + pI:C or mLama4 SLP + pI:C (SQ)
Tumor Disaggregation
Surface + pMHC-I Tetramer Staining
Y1.7AI : mAlg8MHC-I mItgb1MHC-II
Y1.7LI: mLama4MHC-I mItgb1MHC-II
Control mAb
αCTLA-4
pI:C
neoAg SLP VAX (mAlg8 SLP+pI:C)
neoAg SLP VAX (mLama4 SLP+pI:C)
1.97%
3.15%
1.73%
6.49%
0.66%
0.60%
0.53%
0.30%
0.13%
0.52%
0.08%
0.63%
0.61%
1.15%
1.39%
10.5%
mAlg8-H2-Kb-PE
mAlg8-H2-Kb-APC
mLama4-H2-Kb-PE
mLama4-H2-Kb-APC
F
Y1.7AI : mAlg8MHC-I mItgb1MHC-II
PD-1+ TIM-3+ % of mAlg8-H2-Kb+
Y1.7LI: mLama4MHC-I mItgb1MHC-II
PD-1+ TIM-3+ % of mLama4-H2-Kb+
Control mAb
αCTLA-4
pI:C
neoAg SLP Vax (mAlg8 SLP + pI:C)
neoAg SLP Vax (mLama4 SLP + pI:C)
NS
*
**

Figure S1. NeoAg SLP vaccines and ICT induces long-term tumor protection in Y1.7AI and Y1.7LI melanoma models. Related to Figure 1.

(**A**) Schematic of neoAg minigenes used to express neoAgs in the parental *Braf*^{V600E} *Pten*$^{-/-}$ *Cdkn2a*$^{-/-}$ YUMM1.7 melanoma line, along with peptide sequences encoded by minigenes. mLama4 or mAlg8 and mItgb1 neoAg minigene coding sequences were separated by 2A peptide sequences that induce ribosomal skipping during translation. (**B**) Tumor growth in WT C57BL/6J mice transplanted with parental *Braf*^{V600E} *Pten*$^{-/-}$ *Cdkn2a*$^{-/-}$ YUMM1.7 melanoma cells and treated with control mAb, anti-CTLA-4, anti-PD-1 or anti-CTLA4 + anti-PD-1 combination immune checkpoint therapy (ICT) on d. 3, 6, 9, 12, 18, 24 post tumor-transplant. (**C**) WT C57BL/6J mice were transplanted with Y1.7 mA^{MHC-I}.mI^{MHC-II} (Y1.7AI) and Y1.7 mL^{MHC-I}.mI^{MHC-II} (Y1.7LI) melanoma cells and treated with control mAb or anti-CTLA-4 on d. 3, 6, 9, 12, 18, 24 or mAlg8 neoAg (relevant for Y1.7AI) synthetic long peptide (SLP) + poly I:C (pI:C) (neoAg SLP Vax) or mLama4 neoAg (relevant for Y1.7LI) synthetic long peptide (SLP) + pI:C (neoAg SLP Vax) on d. 3, 9, 15. Mice were rechallenged with same tumor used for initial tumor challenge at least 60 days post-rejection of primary tumor. Naïve WT C57BL/6J mice transplanted with Y1.7AI or Y1.7LI tumor without any treatment were included as controls, indicating cell line preps used in rechallenge experiments were capable of tumor formation. (**D**) WT C57BL/6J mice were transplanted with Y1.7LI melanoma cells and treated with anti-CTLA-4 ICT on d. 3, 6, 9, 12, 18, 24 or mLama4 neoAg SLP + pI:C (neoAg SLP Vax) on d. 3, 9, 15. Mice were rechallenged with either with the same tumor used for initial tumor challenge (Y1.7LI) or parental *Braf*^{V600E} *Pten*$^{-/-}$ *Cdkn2a*$^{-/-}$ YUMM1.7 at least 60 days post-rejection of primary tumor. Naïve WT C57BL/6J mice transplanted with either Y1.7LI or parental YUMM1.7 without any treatment were included as controls. (**E**) Representative flow cytometry plots displaying mAlg8 or mLama4 tetramer-specific CD8 T cells in Y1.7AI and Y1.7LI tumors treated with control mAb, anti-CTLA-4, pI:C, mAlg8 SLP + pI:C neoAg SLP vaccine (for Y1.7AI) or mLama4 SLP + pI:C neoAg SLP vaccine (for Y1.7LI) and harvested on d. 16 post-tumor transplant. mAlg8-H2-K^b or mLama4-H2-K^b tetramers were labeled with PE and APC. Dot plots are gated on live CD45$^+$ Thy1.2$^+$ CD8 T cells. (**F**) Co-expression of PD-1 and TIM-3 on mAlg8- or mLama4-specific CD8 T cells in Y1.7AI and Y1.7LI tumors treated with control mAb, anti-CTLA-4, pI:C alone, mAlg8 SLP + pI:C neoAg SLP vaccine (for Y1.7AI), or mLama4 SLP + pI:C neoAg SLP vaccine (for Y1.7LI). Tumor growth data in (**B**), (**C**) and (**D**) are presented as individual mouse tumor growth as mean tumor diameter and are representative of three independent experiments. Bar graphs in (**F**) display mean ± SEM and are representative of at least three independent experiments (*$P < 0.05$, **$P < 0.01$, NS, not significant, unpaired t test).

Figure S2

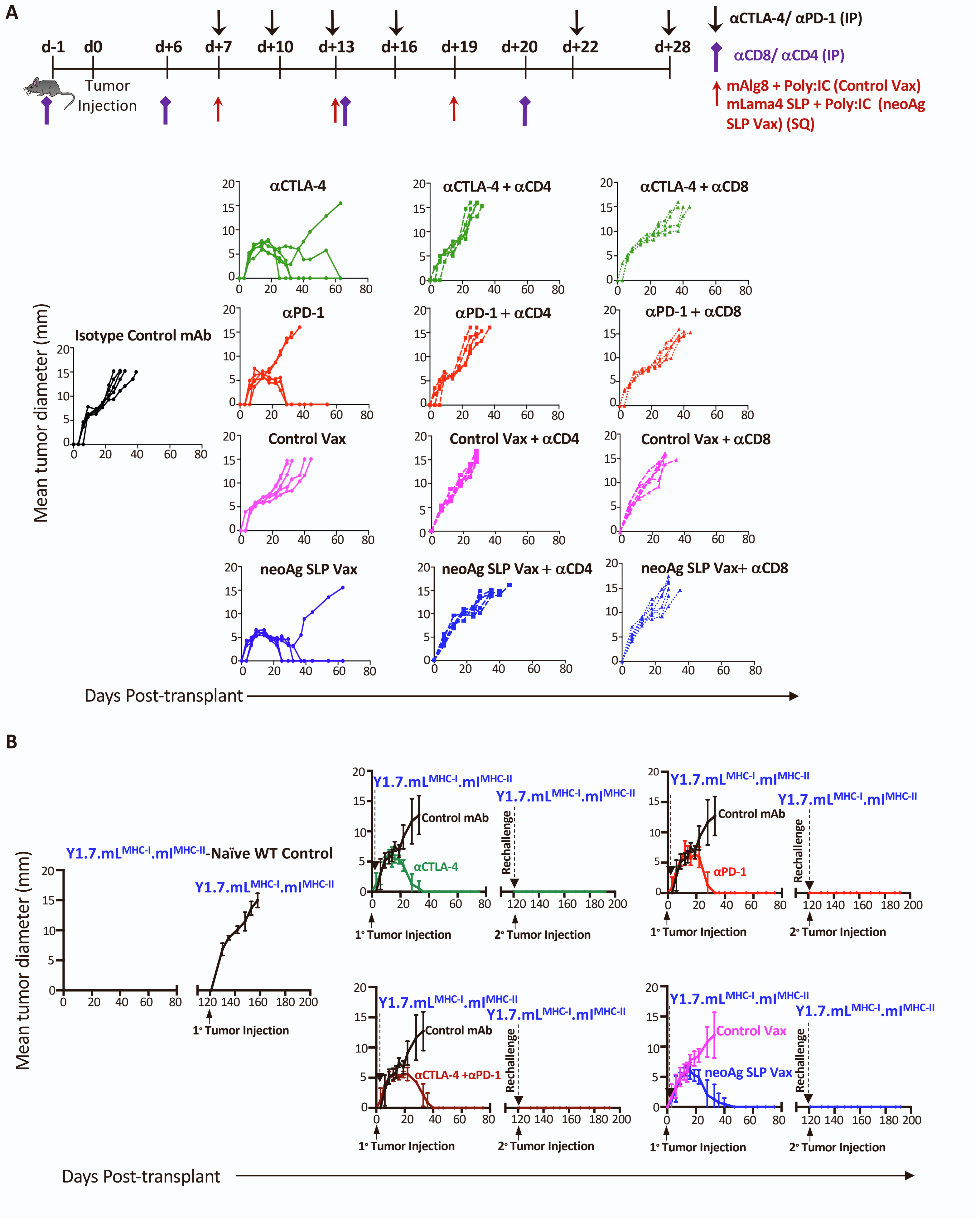
A
d-1
d0
d+6
d+7
d+10
d+13
d+16
d+19
d+20
d+22
d+28
Tumor Injection
αCTLA-4/ αPD-1 (IP)
αCD8/ αCD4 (IP)
mAlg8 + Poly:IC (Control Vax)
mLama4 SLP + Poly:IC (neoAg SLP Vax) (SQ)
Mean tumor diameter (mm)
Isotype Control mAb
αCTLA-4
αCTLA-4 + αCD4
αCTLA-4 + αCD8
αPD-1
αPD-1 + αCD4
αPD-1 + αCD8
Control Vax
Control Vax + αCD4
Control Vax + αCD8
neoAg SLP Vax
neoAg SLP Vax + αCD4
neoAg SLP Vax+ αCD8
Days Post-transplant
B
Y1.7.mL^{MHC-I}.mI^{MHC-II}-Naïve WT Control
Y1.7.mL^{MHC-I}.mI^{MHC-II}
Mean tumor diameter (mm)
1° Tumor Injection
2° Tumor Injection
Rechallenge
Control mAb
αCTLA-4
αPD-1
αCTLA-4 +αPD-1
Control Vax
neoAg SLP Vax
Days Post-transplant

Figure S2. NeoAg SLP vaccines and ICT induce long-term tumor protection in Y1.7LI melanoma models in a T cell-dependent manner. Related to Figure 1.
(**A**) Y1.7LI tumor growth in WT C57BL/6J mice treated with isotype control mAb, anti-CD4, or anti-CD8α mAbs on d. -1, 6, 13, 20 and anti-CTLA-4 or anti-PD-1 on d. 7, 10, 13, 16, 22, 28 or irrelevant mAlg8 SLP + pI:C (Control Vax) or relevant mLama4 SLP + pI:C (neoAg SLP Vax) on d. 7, 13, 19. (**B**) WT C57BL/6J mice transplanted with Y1.7LI melanoma cells were treated with control mAb, anti-CTLA-4, anti-PD-1, anti-CTLA-4 + anti-PD-1, irrelevant (for Y1.7LI) mAlg8 SLP + pI:C (Control Vax), or relevant mLama4 SLP + pI:C (neoAg SLP Vax) starting on d. 7 post tumor-transplant, and subsequently on d. 10, 13, 16, 22, 28 for ICT and d. 13, 19 for Control Vax or neoAg SLP Vax. Mice were rechallenged with the same tumor line used for initial tumor challenge (Y1.7LI) at least 60 days post-rejection of primary tumor. Naïve WT C57BL/6J mice transplanted with Y1.7LI tumor without any treatment was included as a control. Tumor growth data in (**A**) and (**B**) are presented as individual mouse tumor growth as mean tumor diameter and are representative of three independent experiments.

Figure S3

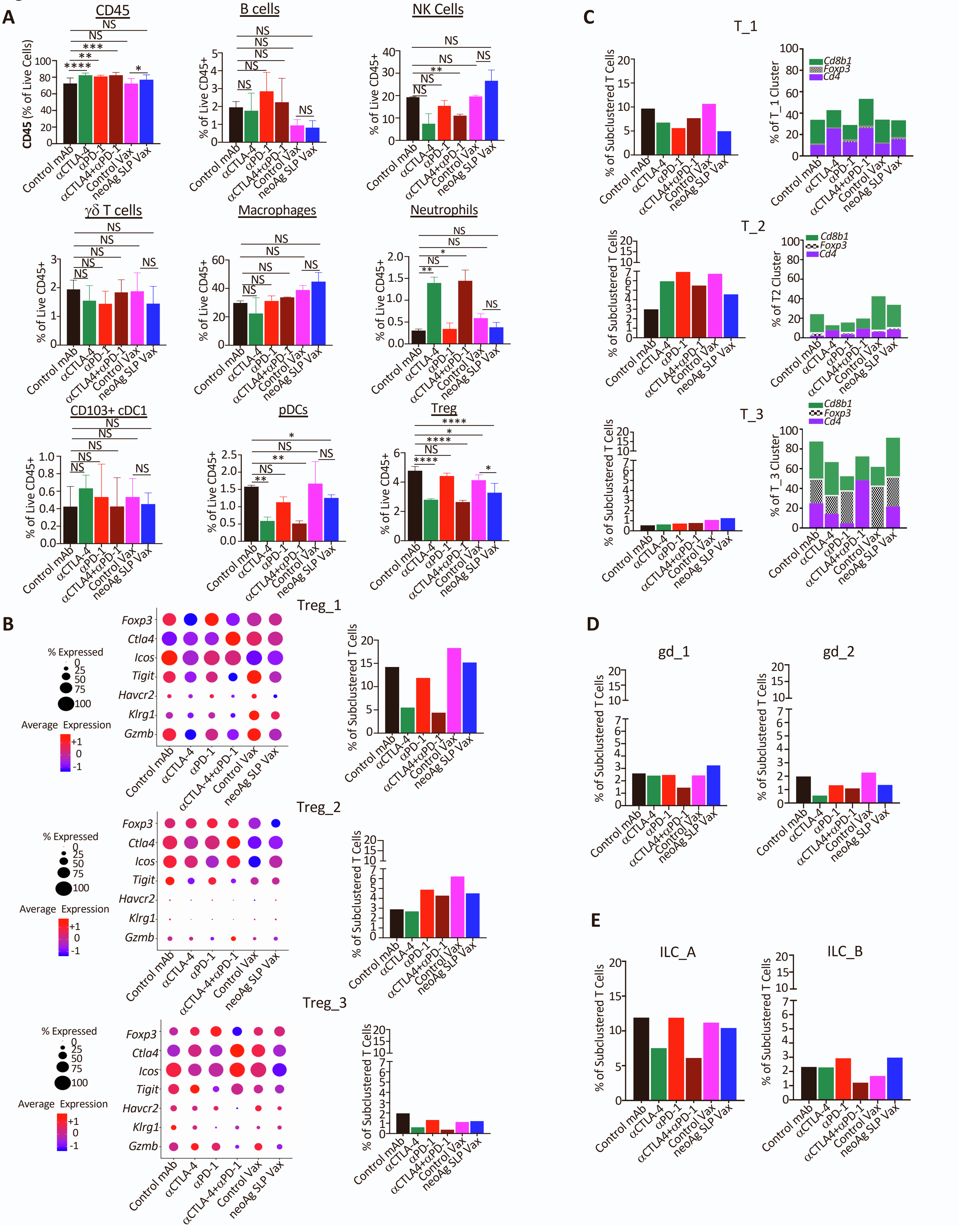

A
Flow Cytometry
CD45
B cells
NK Cells
CD45 (% of Live Cells)
% of Live CD45+
γδ T cells
Macrophages
Neutrophils
CD103+ cDC1
pDCs
Treg
NS
*
**
***
****
Control mAb
αCTLA-4
αPD-1
αCTLA4+αPD-1
Control Vax
neoAg SLP Vax
B
Treg_1
Treg_2
Treg_3
% Expressed
0
25
50
75
100
Average Expression
+1
0
-1
Foxp3
Ctla4
Icos
Tigit
Havcr2
Klrg1
Gzmb
αCTLA-4+αPD-1
% of Subclustered T Cells
C
T_1
T_2
T_3
% of T_1 Cluster
% of T2 Cluster
% of T_3 Cluster
Cd8b1
Foxp3
Cd4
D
gd_1
gd_2
E
ILC_A
ILC_B

Figure S3. Flow cytometry and scRNAseq analysis of Y1.7LI intratumoral lymphoid and myeloid populations. Related to Figure 2.

(**A**) Graph of flow cytometry data displaying intratumoral lymphoid and myeloid cells as a percentage of intratumoral live or live $CD45^+$ cells in Y1.7LI tumors treated with control mAb, anti-CTLA-4, anti-PD-1, anti-CTLA-4 + anti-PD-1, irrelevant (for Y1.7LI) mAlg8 SLP + pI:C (Control Vax), or relevant mLama4 SLP + pI:C (neoAg SLP Vax) beginning on d. 7 post-tumor transplant and harvested on d. 15. (**B**) Dot plot depicting expression level and percent of cells expressing *Foxp3*, *Ctla4*, *Icos, Tigit, Havcr2* (TIM-3)*, Klrg1, Gzmb* and graph displaying frequency of regulatory T cell (Treg) scRNAseq clusters by treatment condition. (**C**) Graph displaying mixed T cell clusters represented as percentage of total subclustered T cells and percentage of $Foxp3^+$ CD4 Tregs, conventional CD4 T cells, or CD8 T cells in clusters T_1, T_2, and T_3 by treatment condition. (**D**) Graph displaying $\gamma\delta$ T cell clusters represented as percentage of total subclustered T cells by treatment condition. (**E**) Graph displaying ILC clusters represented as percentage of total subclustered T cells by treatment condition. Bar graphs in (**A**) display mean ± SEM and are representative of at least three independent experiments (**P* < 0.05, ***P* < 0.01, ****P* < 0.005, *****P* < 0.0001, NS, not significant, unpaired t test).

Figure S4

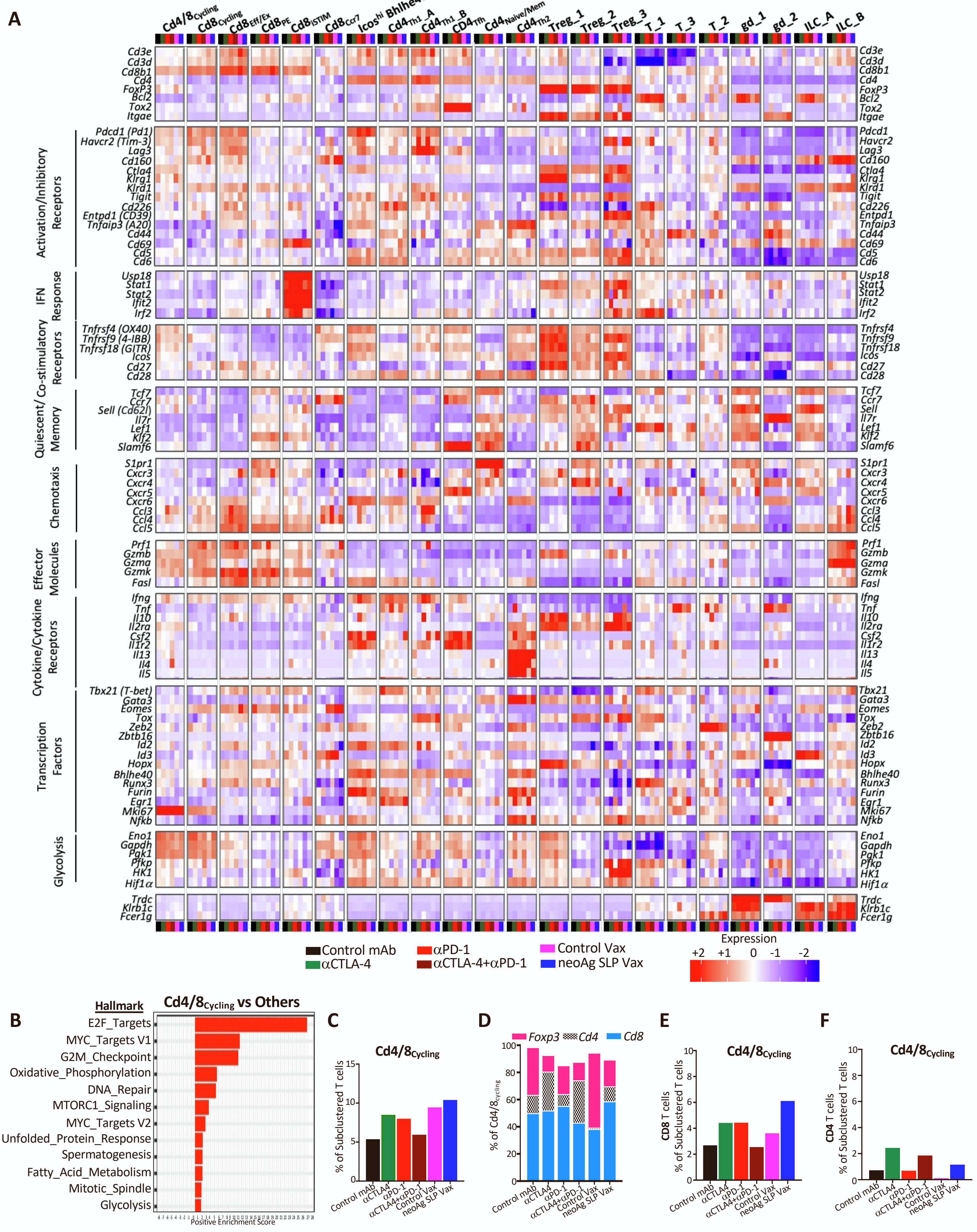
A
Cd4/8Cycling
Cd8Cycling
Cd8Eff/Ex
Cd8PE
Cd8ISTIM
Cd8Ccr7
Icoshi Bhlhe40hi Cd4Th1
Cd4Th1_A
Cd4Th1_B
CD4Tfh
Cd4Naive/Mem
Cd4Th2
Treg_1
Treg_2
Treg_3
T_1
T_3
T_2
gd_1
gd_2
ILC_A
ILC_B
Cd3e
Cd3d
Cd8b1
Cd4
FoxP3
Bcl2
Tox2
Itgae
Activation/Inhibitory Receptors
Pdcd1 (Pd1)
Havcr2 (Tim-3)
Lag3
Cd160
Ctla4
Klrg1
Klrd1
Tigit
Cd226
Entpd1 (CD39)
Tnfaip3 (A20)
Cd44
Cd69
Cd5
Cd6
IFN Response
Usp18
Stat1
Stat2
Ifit2
Irf2
Co-stimulatory Receptors
Tnfrsf4 (OX40)
Tnfrsf9 (4-IBB)
Tnfrsf18 (GITR)
Icos
Cd27
Cd28
Quiescent/ Memory
Tcf7
Ccr7
Sell (Cd62l)
Il7r
Lef1
Klf2
Slamf6
Chemotaxis
S1pr1
Cxcr3
Cxcr4
Cxcr5
Cxcr6
Ccl3
Ccl4
Ccl5
Effector Molecules
Prf1
Gzmb
Gzma
Gzmk
Fasl
Cytokine/Cytokine Receptors
Ifng
Tnf
Il10
Il2ra
Csf2
Il1r2
Il13
Il4
Il5
Transcription Factors
Tbx21 (T-bet)
Gata3
Eomes
Tox
Zeb2
Zbtb16
Id2
Id3
Hopx
Bhlhe40
Runx3
Furin
Egr1
Mki67
Nfkb
Glycolysis
Eno1
Gapdh
Pgk1
Pfkp
HK1
Hif1α
Trdc
Klrb1c
Fcer1g
Control mAb
αCTLA-4
αPD-1
αCTLA-4+αPD-1
Control Vax
neoAg SLP Vax
Expression
+2 +1 0 -1 -2
B
Hallmark
Cd4/8Cycling vs Others
E2F_Targets
MYC_Targets V1
G2M_Checkpoint
Oxidative_Phosphorylation
DNA_Repair
MTORC1_Signaling
MYC_Targets V2
Unfolded_Protein_Response
Spermatogenesis
Fatty_Acid_Metabolism
Mitotic_Spindle
Glycolysis
Positive Enrichment Score
C
Cd4/8Cycling
% of Subclustered T cells
0
5
10
15
Control mAb
αCTLA4
αPD-1
αCTLA4+αPD-1
Control Vax
neoAg SLP Vax
D
Foxp3
Cd4
Cd8
% of Cd4/8cycling
0
20
40
60
80
100
Control mAb
αCTLA4
αPD-1
αCTLA4+αPD-1
Control Vax
neoAg SLP Vax
E
Cd4/8Cycling
CD8 T cells % of Subclustered T cells
0
2
4
6
8
10
Control mAb
αCTLA4
αPD-1
αCTLA4+αPD-1
Control Vax
neoAg SLP Vax
F
Cd4/8Cycling
CD4 T cells % of Subclustered T cells
0
2
4
6
8
10
Control mAb
αCTLA4
αPD-1
αCTLA4+αPD-1
Control Vax
neoAg SLP Vax

Figure S4. scRNAseq of subclustered T cells from Y1.7LI tumor bearing mice treated with neoAg SLP vaccines or ICT. Related to Figure 2.

(**A**) Heat map displaying normalized expression of select genes in each T cell/ILC cluster by treatment condition. (**B**) Gene set enrichment analysis (GSEA) displaying significantly enriched gene sets in cluster $Cd4/8_{Cycling}$. (**C**) Proliferating T cells in cluster $Cd4/8_{Cycling}$ by treatment condition represented as percentage of subclustered T cells. (**D**) Percentage of $Foxp3^{+}$ Tregs, conventional CD4 T cells, or CD8 T cells in $Cd4/8_{Cycling}$ by treatment condition. (**E**) Graph displaying CD8 T cells from cluster $Cd4/8_{Cycling}$ represented as percentage of total subclustered T cells. (**F**) Graph displaying conventional CD4 T cells from cluster $Cd4/8_{Cycling}$ represented as percentage of total subclustered T cells.

Figure S5

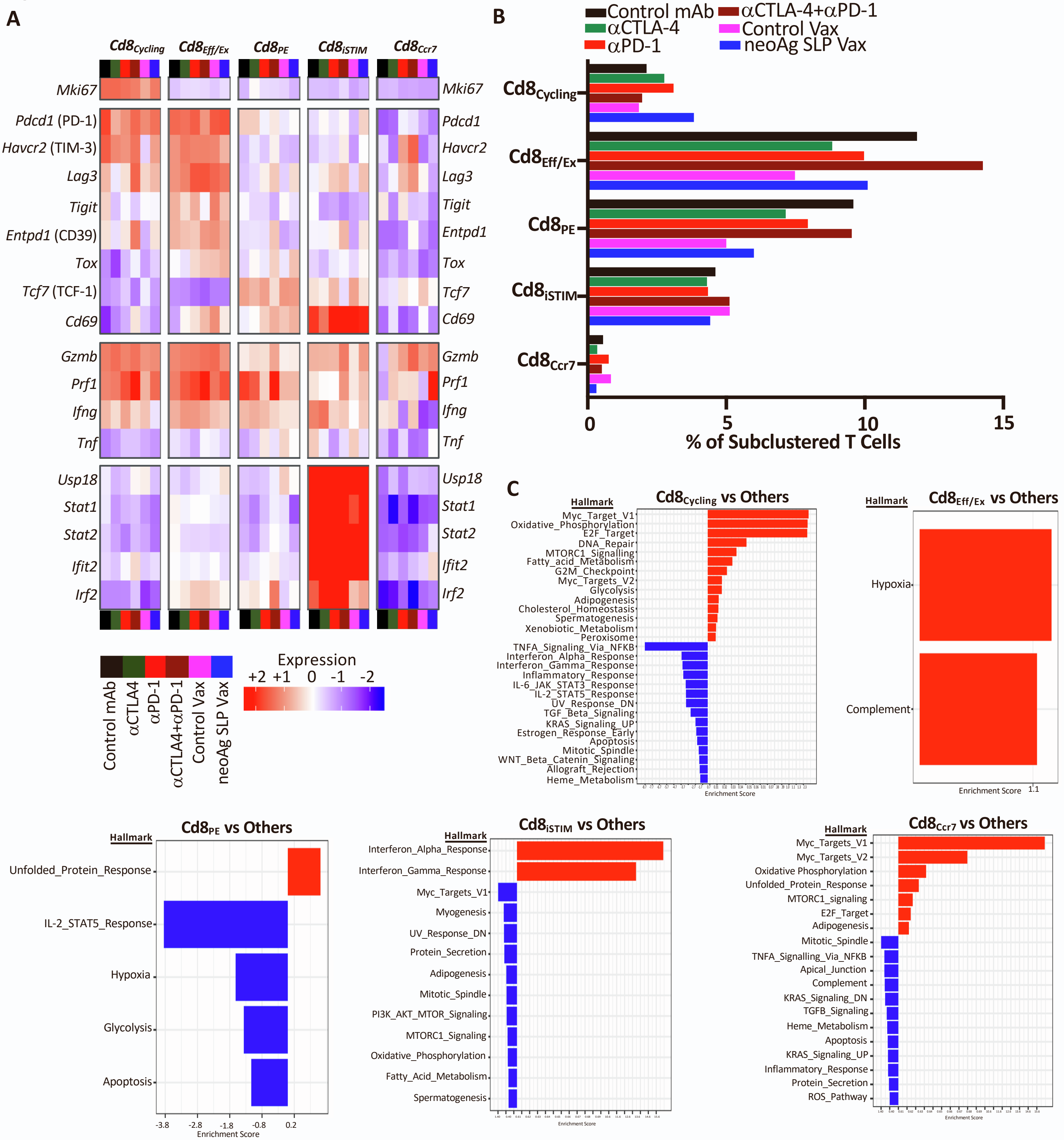
A
Cd8Cycling
Cd8Eff/Ex
Cd8PE
Cd8iSTIM
Cd8Ccr7
Mki67
Pdcd1 (PD-1)
Havcr2 (TIM-3)
Lag3
Tigit
Entpd1 (CD39)
Tox
Tcf7 (TCF-1)
Cd69
Gzmb
Prf1
Ifng
Tnf
Usp18
Stat1
Stat2
Ifit2
Irf2
Pdcd1
Havcr2
Entpd1
Tcf7
Control mAb
αCTLA4
αPD-1
αCTLA4+αPD-1
Control Vax
neoAg SLP Vax
Expression
+2 +1 0 -1 -2
B
Control mAb
αCTLA-4
αPD-1
αCTLA-4+αPD-1
Control Vax
neoAg SLP Vax
Cd8Cycling
Cd8Eff/Ex
Cd8PE
Cd8iSTIM
Cd8Ccr7
0
5
10
15
% of Subclustered T Cells
C
Cd8Cycling vs Others
Hallmark
Myc_Target_V1
Oxidative_Phosphorylation
E2F_Target
DNA_Repair
MTORC1_Signalling
Fatty_acid_Metabolism
G2M_Checkpoint
Myc_Targets_V2
Glycolysis
Adipogenesis
Cholesterol_Homeostasis
Spermatogenesis
Xenobiotic_Metabolism
Peroxisome
TNFA_Signaling_Via_NFKB
Interferon_Alpha_Response
Interferon_Gamma_Response
Inflammatory_Response
IL-6_JAK_STAT3_Response
IL-2_STAT5_Response
UV_Response_DN
TGF_Beta_Signaling
KRAS_Signaling_UP
Estrogen_Response_Early
Apoptosis
Mitotic_Spindle
WNT_Beta_Catenin_Signaling
Allograft_Rejection
Heme_Metabolism
Enrichment Score
Cd8Eff/Ex vs Others
Hallmark
Hypoxia
Complement
Enrichment Score
1.1
Cd8PE vs Others
Hallmark
Unfolded_Protein_Response
IL-2_STAT5_Response
Hypoxia
Glycolysis
Apoptosis
-3.8
-2.8
-1.8
-0.8
0.2
Enrichment Score
Cd8iSTIM vs Others
Hallmark
Interferon_Alpha_Response
Interferon_Gamma_Response
Myc_Targets_V1
Myogenesis
UV_Response_DN
Protein_Secretion
Adipogenesis
Mitotic_Spindle
PI3K_AKT_MTOR_Signaling
MTORC1_Signaling
Oxidative_Phosphorylation
Fatty_Acid_Metabolism
Spermatogenesis
Enrichment Score
Cd8Ccr7 vs Others
Hallmark
Myc_Targets_V1
Myc_Targets_V2
Oxidative Phosphorylation
Unfolded_Protein_Response
MTORC1_signaling
E2F_Target
Adipogenesis
Mitotic_Spindle
TNFA_Signalling_Via_NFKB
Apical_Junction
Complement
KRAS_Signaling_DN
TGFB_Signaling
Heme_Metabolism
Apoptosis
KRAS_Signaling_UP
Inflammatory_Response
Protein_Secretion
ROS_Pathway
Enrichment Score

Figure S5. scRNAseq analysis of bulk CD8 T cells from Y1.7LI tumor bearing mice treated with neoAg SLP vaccines or ICT. Related to Figure 2.
(**A**) Heat map displaying normalized expression of select genes in each bulk CD8 T cell clusters (see also Figures 2A and 2D). (**B**) Bar graphs depicting frequency of each CD8 T cell cluster by treatment condition. (**C**) GSEA displaying significantly enriched gene sets in each CD8 T cell cluster.

Figure S6

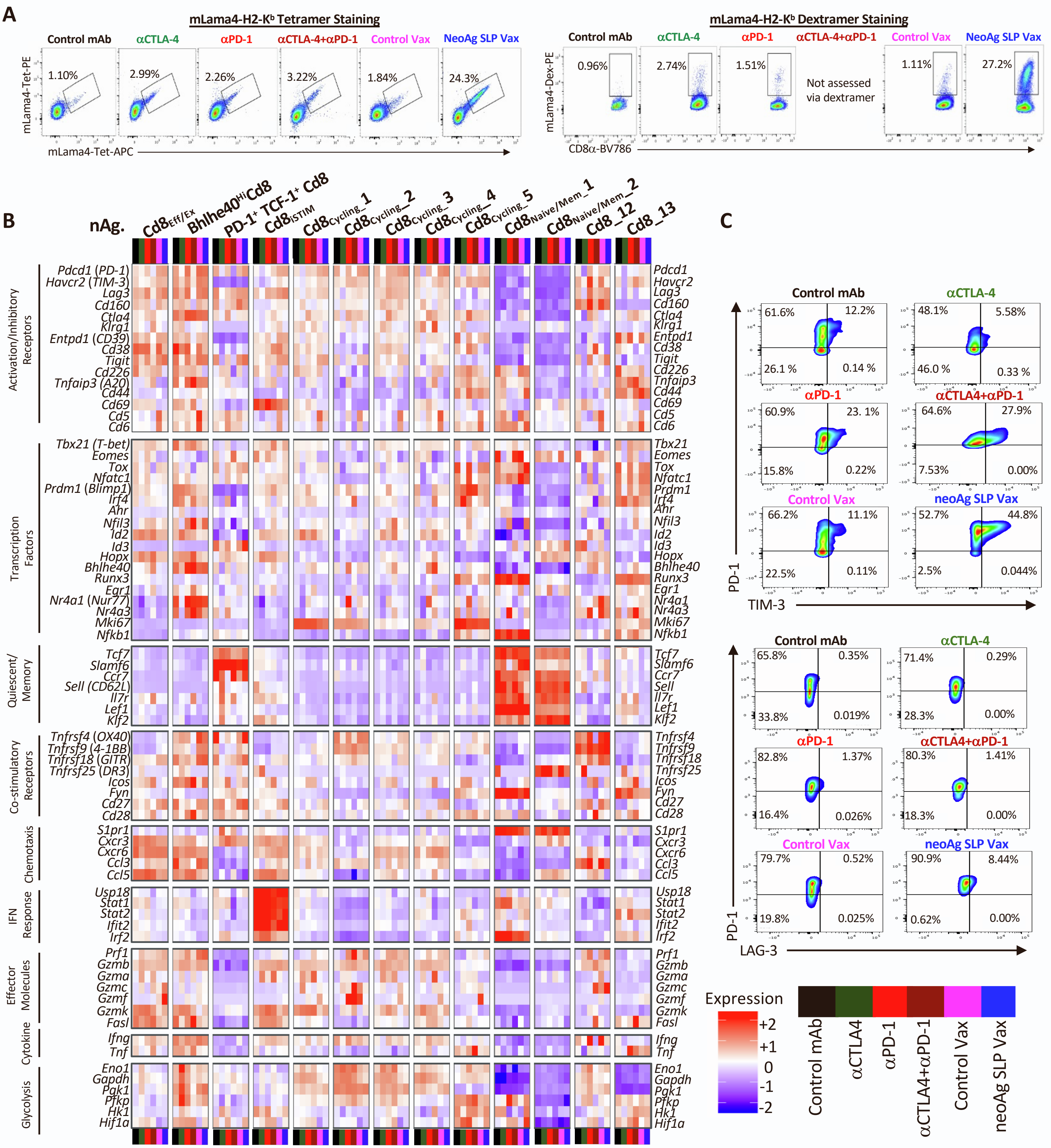
A
mLama4-H2-K^b Tetramer Staining
Control mAb
αCTLA-4
αPD-1
αCTLA-4+αPD-1
Control Vax
NeoAg SLP Vax
1.10%
2.99%
2.26%
3.22%
1.84%
24.3%
mLama4-Tet-PE
mLama4-Tet-APC
mLama4-H2-K^b Dextramer Staining
0.96%
2.74%
1.51%
Not assessed via dextramer
1.11%
27.2%
mLama4-Dex-PE
CD8α-BV786
B
nAg.
Cd8Eff/Ex
Bhlhe40HiCd8
PD-1+ TCF-1+ Cd8
Cd8ISTIM
Cd8Cycling_1
Cd8Cycling_2
Cd8Cycling_3
Cd8Cycling_4
Cd8Cycling_5
Cd8Naive/Mem_1
Cd8Naive/Mem_2
Cd8_12
Cd8_13
Activation/Inhibitory Receptors
Pdcd1 (PD-1)
Havcr2 (TIM-3)
Lag3
Cd160
Ctla4
Klrg1
Entpd1 (CD39)
Cd38
Tigit
Cd226
Tnfaip3 (A20)
Cd44
Cd69
Cd5
Cd6
Transcription Factors
Tbx21 (T-bet)
Eomes
Tox
Nfatc1
Prdm1 (Blimp1)
Irf4
Ahr
Nfil3
Id2
Id3
Hopx
Bhlhe40
Runx3
Egr1
Nr4a1 (Nur77)
Nr4a3
Mki67
Nfkb1
Quiescent/Memory
Tcf7
Slamf6
Ccr7
Sell (CD62L)
Il7r
Lef1
Klf2
Co-stimulatory Receptors
Tnfrsf4 (OX40)
Tnfrsf9 (4-1BB)
Tnfrsf18 (GITR)
Tnfrsf25 (DR3)
Icos
Fyn
Cd27
Cd28
Chemotaxis
S1pr1
Cxcr3
Cxcr6
Ccl3
Ccl5
IFN Response
Usp18
Stat1
Stat2
Ifit2
Irf2
Effector Molecules
Prf1
Gzmb
Gzma
Gzmc
Gzmf
Gzmk
Fasl
Cytokine
Ifng
Tnf
Glycolysis
Eno1
Gapdh
Pgk1
Pfkp
Hk1
Hif1a
Expression
+2
+1
0
-1
-2
Control mAb
αCTLA4
αPD-1
αCTLA4+αPD-1
Control Vax
neoAg SLP Vax
C
Control mAb
61.6%
12.2%
26.1 %
0.14 %
αCTLA-4
48.1%
5.58%
46.0 %
0.33 %
αPD-1
60.9%
23. 1%
15.8%
0.22%
αCTLA4+αPD-1
64.6%
27.9%
7.53%
0.00%
Control Vax
66.2%
11.1%
22.5%
0.11%
neoAg SLP Vax
52.7%
44.8%
2.5%
0.044%
PD-1
TIM-3
Control mAb
65.8%
0.35%
33.8%
0.019%
αCTLA-4
71.4%
0.29%
28.3%
0.00%
αPD-1
82.8%
1.37%
16.4%
0.026%
αCTLA4+αPD-1
80.3%
1.41%
18.3%
0.00%
Control Vax
79.7%
0.52%
19.8%
0.025%
neoAg SLP Vax
90.9%
8.44%
0.62%
0.00%
PD-1
LAG-3

Figure S6. scRNAseq and flow cytometry profiling of mLama4 neoAg-specific CD8 T cells from Y1.7LI tumor bearing mice treated with neoAg SLP vaccines or ICT. Related to Figure 3.

(**A**) Representative flow cytometry plots displaying mLama4-specific (assessed by tetramer and dextramer staining) CD8 T cells in Y1.7LI tumors treated with control mAb, anti-CTLA-4, anti-PD-1, anti-CTLA-4 + anti-PD-1, irrelevant (for Y1.7LI) mAlg8 SLP + pI:C (Control Vax), or relevant mLama4 SLP + pI:C (neoAg SLP Vax) beginning on d. 7 and harvested on d. 15 post-tumor transplant. mLama4-H2-K^b tetramers were labeled with PE and APC and mLama4-H2-K^b dextramer was labeled with PE. Dot plots are gated on live $CD45^+$ $Thy1.2^+$ CD8 T cells (See also Figure S12). (**B**) Heat map displaying normalized expression of select genes in each mLama4 neoAg-specific CD8 T cell clusters by treatment condition (see also Figure 3E). (**C**) Representative flow cytometry plots displaying $PD\text{-}1^+$ and/or $TIM\text{-}3^+$/$LAG\text{-}3^+$ after gating on mLama4 tetramer positive CD8 T cells (See also Figure S12).

Figure S7

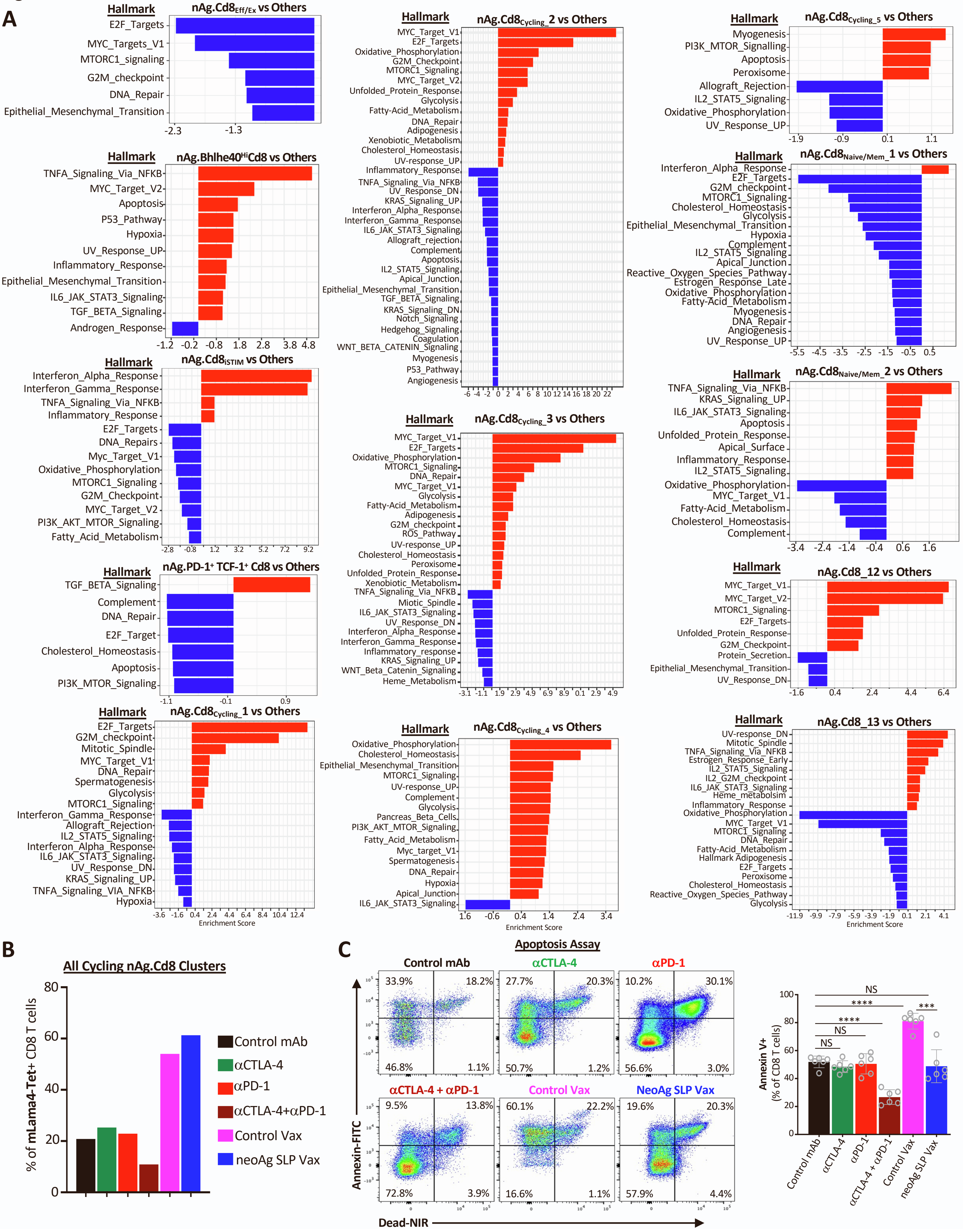
A
Hallmark
nAg.Cd8Eff/Ex vs Others
E2F_Targets
MYC_Targets_V1
MTORC1_signaling
G2M_checkpoint
DNA_Repair
Epithelial_Mesenchymal_Transition
Hallmark
nAg.Bhlhe40HiCd8 vs Others
TNFA_Signaling_Via_NFKB
MYC_Target_V2
Apoptosis
P53_Pathway
Hypoxia
UV_Response_UP
Inflammatory_Response
Epithelial_Mesenchymal_Transition
IL6_JAK_STAT3_Signaling
TGF_BETA_Signaling
Androgen_Response
Hallmark
nAg.Cd8ISTIM vs Others
Interferon_Alpha_Response
Interferon_Gamma_Response
TNFA_Signaling_Via_NFKB
Inflammatory_Response
E2F_Targets
DNA_Repairs
Myc_Target_V1
Oxidative_Phosphorylation
MTORC1_Signaling
G2M_Checkpoint
MYC_Target_V2
PI3K_AKT_MTOR_Signaling
Fatty_Acid_Metabolism
Hallmark
nAg.PD-1+ TCF-1+ Cd8 vs Others
TGF_BETA_Signaling
Complement
DNA_Repair
E2F_Target
Cholesterol_Homeostasis
Apoptosis
PI3K_MTOR_Signaling
Hallmark
nAg.Cd8Cycling_1 vs Others
E2F_Targets
G2M_checkpoint
Mitotic_Spindle
MYC_Target_V1
DNA_Repair
Spermatogenesis
Glycolysis
MTORC1_Signaling
Interferon_Gamma_Response
Allograft_Rejection
IL2_STAT5_Signaling
Interferon_Alpha_Response
IL6_JAK_STAT3_Signaling
UV_Response_DN
KRAS_Signaling_UP
TNFA_Signaling_VIA_NFKB
Hypoxia
Enrichment Score
Hallmark
nAg.Cd8Cycling_2 vs Others
MYC_Target_V1
E2F_Targets
Oxidative_Phosphorylation
G2M_Checkpoint
MTORC1_Signaling
MYC_Target_V2
Unfolded_Protein_Response
Glycolysis
Fatty-Acid_Metabolism
DNA_Repair
Adipogenesis
Xenobiotic_Metabolism
Cholesterol_Homeostasis
UV-response_UP
Inflammatory_Response
TNFA_Signaling_Via_NFKB
UV_Response_DN
KRAS_Signaling_UP
Interferon_Alpha_Response
Interferon_Gamma_Response
IL6_JAK_STAT3_Signaling
Allograft_rejection
Complement
Apoptosis
IL2_STAT5_Signaling
Apical_Junction
Epithelial_Mesenchymal_Transition
TGF_BETA_Signaling
KRAS_Signaling_DN
Notch_Signaling
Hedgehog_Signaling
Coagulation
WNT_BETA_CATENIN_Signaling
Myogenesis
P53_Pathway
Angiogenesis
Hallmark
nAg.Cd8Cycling_3 vs Others
MYC_Target_V1
E2F_Targets
Oxidative_Phosphorylation
MTORC1_Signaling
DNA_Repair
MYC_Target_V1
Glycolysis
Fatty-Acid_Metabolism
Adipogenesis
G2M_checkpoint
ROS_Pathway
UV-response_UP
Cholesterol_Homeostasis
Peroxisome
Unfolded_Protein_Response
Xenobiotic_Metabolism
TNFA_Signaling_Via_NFKB
Miotic_Spindle
IL6_JAK_STAT3_Signaling
UV_Response_DN
Interferon_Alpha_Response
Interferon_Gamma_Response
Inflammatory_response
KRAS_Signaling_UP
WNT_Beta_Catenin_Signaling
Heme_Metabolism
Hallmark
nAg.Cd8Cycling_4 vs Others
Oxidative_Phosphorylation
Cholesterol_Homeostasis
Epithelial_Mesenchymal_Transition
MTORC1_Signaling
UV-response_UP
Complement
Glycolysis
Pancreas_Beta_Cells
PI3K_AKT_MTOR_Signaling
Fatty_Acid_Metabolism
Myc_target_V1
Spermatogenesis
DNA_Repair
Hypoxia
Apical_Junction
IL6_JAK_STAT3_Signaling
Enrichment Score
Hallmark
nAg.Cd8Cycling_5 vs Others
Myogenesis
PI3K_MTOR_Signalling
Apoptosis
Peroxisome
Allograft_Rejection
IL2_STAT5_Signaling
Oxidative_Phosphorylation
UV_Response_UP
Hallmark
nAg.Cd8Naive/Mem_1 vs Others
Interferon_Alpha_Response
E2F_Targets
G2M_checkpoint
MTORC1_Signaling
Cholesterol_Homeostasis
Glycolysis
Epithelial_Mesenchymal_Transition
Hypoxia
Complement
IL2_STAT5_Signaling
Apical_Junction
Reactive_Oxygen_Species_Pathway
Estrogen_Response_Late
Oxidative_Phosphorylation
Fatty-Acid_Metabolism
Myogenesis
DNA_Repair
Angiogenesis
UV_Response_UP
Hallmark
nAg.Cd8Naive/Mem_2 vs Others
TNFA_Signaling_Via_NFKB
KRAS_Signaling_UP
IL6_JAK_STAT3_Signaling
Apoptosis
Unfolded_Protein_Response
Apical_Surface
Inflammatory_Response
IL2_STAT5_Signaling
Oxidative_Phosphorylation
MYC_Target_V1
Fatty-Acid_Metabolism
Cholesterol_Homeostasis
Complement
Hallmark
nAg.Cd8_12 vs Others
MYC_Target_V1
MYC_Target_V2
MTORC1_Signaling
E2F_Targets
Unfolded_Protein_Response
G2M_Checkpoint
Protein_Secretion
Epithelial_Mesenchymal_Transition
UV_Response_DN
Hallmark
nAg.Cd8_13 vs Others
UV-response_DN
Mitotic_Spindle
TNFA_Signaling_Via_NFKB
Estrogen_Response_Early
IL2_STAT5_Signaling
IL2_G2M_checkpoint
IL6_JAK_STAT3_Signaling
Heme_metabolism
Inflammatory_Response
Oxidative_Phosphorylation
MYC_Target_V1
MTORC1_Signaling
DNA_Repair
Fatty-Acid_Metabolism
Hallmark Adipogenesis
E2F_Targets
Peroxisome
Cholesterol_Homeostasis
Reactive_Oxygen_Species_Pathway
Glycolysis
Enrichment Score
B
All Cycling nAg.Cd8 Clusters
% of mLama4-Tet+ CD8 T cells
Control mAb
αCTLA-4
αPD-1
αCTLA-4+αPD-1
Control Vax
neoAg SLP Vax
C
Apoptosis Assay
Control mAb
33.9%
18.2%
46.8%
1.1%
αCTLA-4
27.7%
20.3%
50.7%
1.2%
αPD-1
10.2%
30.1%
56.6%
3.0%
αCTLA-4 + αPD-1
9.5%
13.8%
72.8%
3.9%
Control Vax
60.1%
22.2%
16.6%
1.1%
NeoAg SLP Vax
19.6%
20.3%
57.9%
4.4%
Annexin-FITC
Dead-NIR
Annexin V+ (% of CD8 T cells)
NS
****
****
NS
NS
***
Control mAb
αCTLA-4
αPD-1
αCTLA-4 + αPD-1
Control Vax
neoAg SLP Vax

Figure S7. GSEA on neoAg-Specific CD8 T Cell Clusters, Total Cycling neoAg-Specific CD8 T Cells, and CD8 T Cell Apoptosis Assay. Related to Figure 3. (**A**) GSEA displaying significantly enriched gene sets for each neoAg-specific CD8 T cells cluster (**B**) Frequency of total mLama4 neoAg-specific CD8 T cells by treatment condition within the 5 cycling clusters combined. (**C**) Dot plots and bar graph displaying Annexin V and viability dye (NIR) staining gated on intratumoral CD8 T cells from Y1.7LI tumors treated with control mAb, anti-CTLA-4, anti-PD-1, anti-CTLA-4 + anti-PD-1, irrelevant (for Y1.7LI) mAlg8 SLP + pI:C (Control Vax), or relevant mLama4 SLP + pI:C (neoAg SLP Vax) beginning on d. 7 and harvested on d. 15 post-tumor transplant. Bar graphs in (**C**) display mean ± SEM and are representative of at least three independent experiments (****P* < 0.005, *****P* < 0.0001, NS, not significant, unpaired t test).

Figure S8

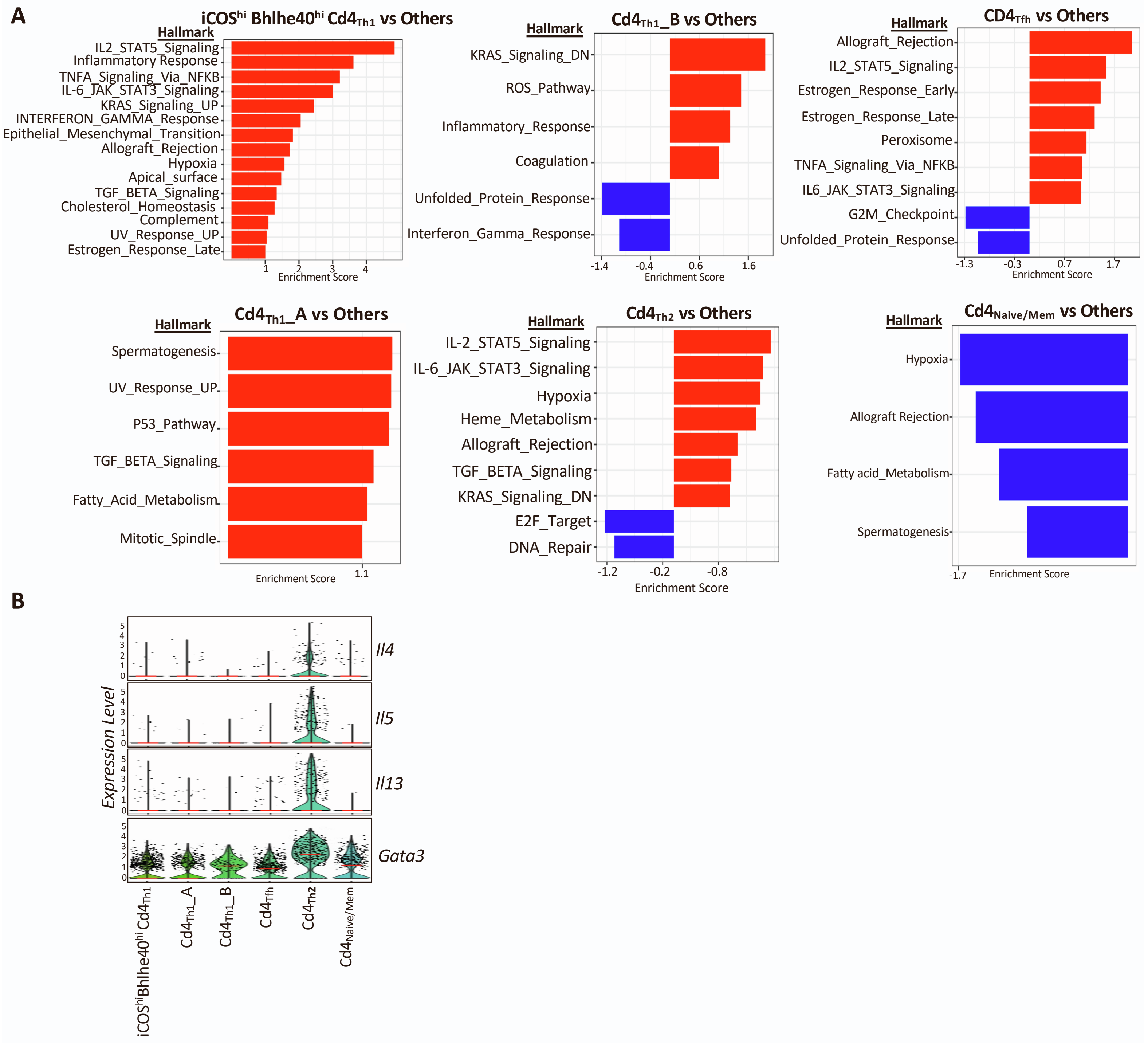
A
iCOS^hi Bhlhe40^hi Cd4Th1 vs Others
Hallmark
IL2_STAT5_Signaling
Inflammatory Response
TNFA_Signaling_Via_NFKB
IL-6_JAK_STAT3_Signaling
KRAS_Signaling_UP
INTERFERON_GAMMA_Response
Epithelial_Mesenchymal_Transition
Allograft_Rejection
Hypoxia
Apical_surface
TGF_BETA_Signaling
Cholesterol_Homeostasis
Complement
UV_Response_UP
Estrogen_Response_Late
Enrichment Score
Cd4Th1_B vs Others
KRAS_Signaling_DN
ROS_Pathway
Inflammatory_Response
Coagulation
Unfolded_Protein_Response
Interferon_Gamma_Response
CD4Tfh vs Others
Allograft_Rejection
IL2_STAT5_Signaling
Estrogen_Response_Early
Estrogen_Response_Late
Peroxisome
TNFA_Signaling_Via_NFKB
IL6_JAK_STAT3_Signaling
G2M_Checkpoint
Unfolded_Protein_Response
Cd4Th1_A vs Others
Spermatogenesis
UV_Response_UP
P53_Pathway
TGF_BETA_Signaling
Fatty_Acid_Metabolism
Mitotic_Spindle
Cd4Th2 vs Others
IL-2_STAT5_Signaling
IL-6_JAK_STAT3_Signaling
Hypoxia
Heme_Metabolism
Allograft_Rejection
TGF_BETA_Signaling
KRAS_Signaling_DN
E2F_Target
DNA_Repair
Cd4Naive/Mem vs Others
Hypoxia
Allograft Rejection
Fatty acid_Metabolism
Spermatogenesis
B
Expression Level
Il4
Il5
Il13
Gata3
iCOS^hi Bhlhe40^hi Cd4Th1
Cd4Th1_A
Cd4Th1_B
Cd4Tfh
Cd4Th2
Cd4Naive/Mem

Figure S8. Cluster-specific enriched gene sets and select transcript alterations in conventional CD4 T cells (see also Figure 2A). Related to Figure 5.
(**A**) GSEA displaying significantly enriched gene sets within each CD4 T cell cluster by treatment condition (see also Figure 2D). (**B**) Violin plots denoting expression level of select genes per CD4 T cell for each CD4 T cell cluster.

Figure S9

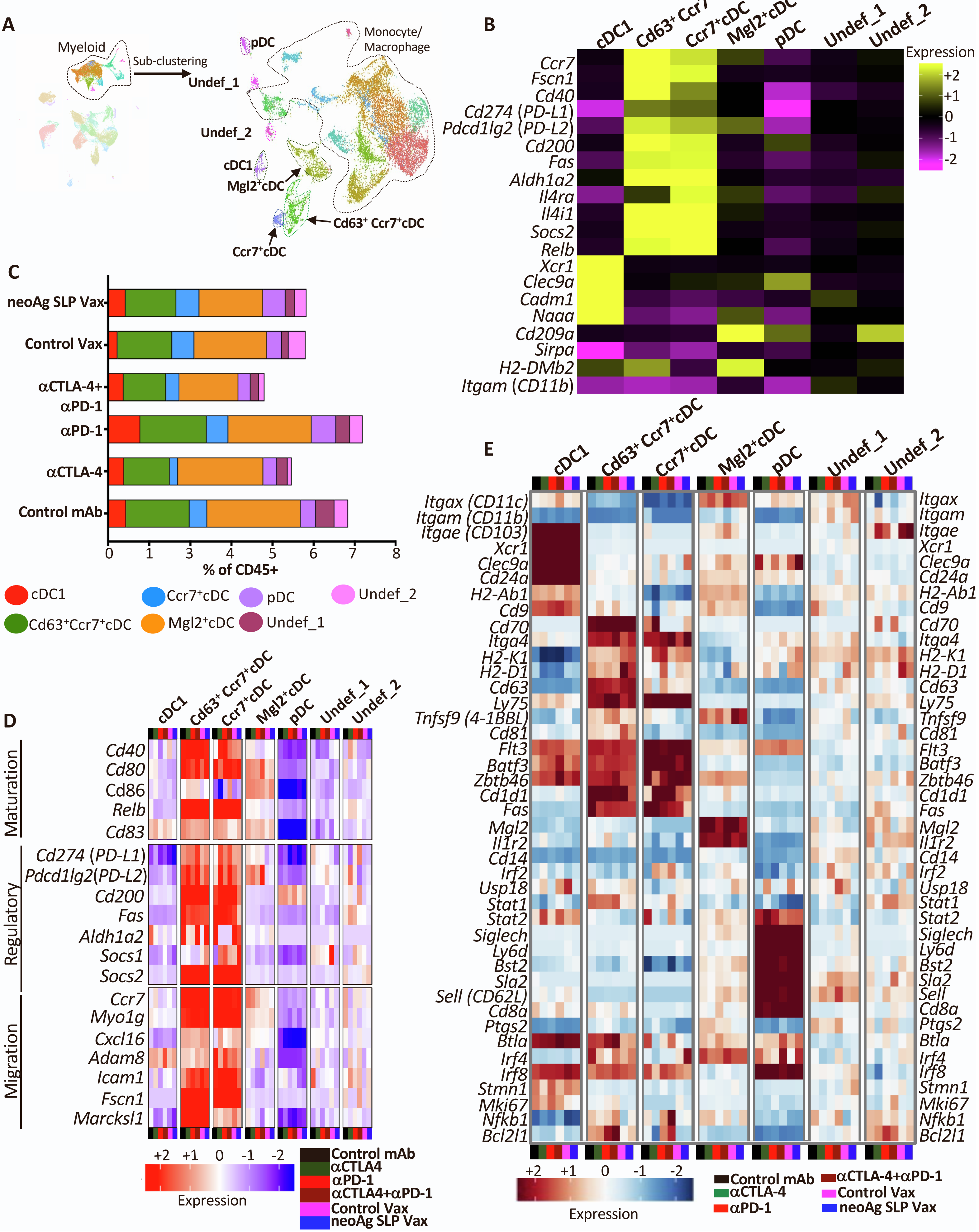
A
Myeloid
Sub-clustering
pDC
Monocyte/ Macrophage
Undef_1
Undef_2
cDC1
Mgl2+cDC
Cd63+ Ccr7+cDC
Ccr7+cDC
B
cDC1
Cd63+ Ccr7+cDC
Ccr7+cDC
Mgl2+cDC
pDC
Undef_1
Undef_2
Expression
+2
+1
0
-1
-2
Ccr7
Fscn1
Cd40
Cd274 (PD-L1)
Pdcd1lg2 (PD-L2)
Cd200
Fas
Aldh1a2
Il4ra
Il4i1
Socs2
Relb
Xcr1
Clec9a
Cadm1
Naaa
Cd209a
Sirpa
H2-DMb2
Itgam (CD11b)
C
neoAg SLP Vax
Control Vax
αCTLA-4+αPD-1
αPD-1
αCTLA-4
Control mAb
0 1 2 3 4 5 6 7 8
% of CD45+
cDC1
Cd63+Ccr7+cDC
Ccr7+cDC
Mgl2+cDC
pDC
Undef_1
Undef_2
D
cDC1
Cd63+ Ccr7+cDC
Ccr7+cDC
Mgl2+cDC
pDC
Undef_1
Undef_2
Maturation
Cd40
Cd80
Cd86
Relb
Cd83
Regulatory
Cd274 (PD-L1)
Pdcd1lg2(PD-L2)
Cd200
Fas
Aldh1a2
Socs1
Socs2
Migration
Ccr7
Myo1g
Cxcl16
Adam8
Icam1
Fscn1
Marcksl1
+2 +1 0 -1 -2
Expression
Control mAb
αCTLA4
αPD-1
αCTLA4+αPD-1
Control Vax
neoAg SLP Vax
E
cDC1
Cd63+ Ccr7+cDC
Ccr7+cDC
Mgl2+cDC
pDC
Undef_1
Undef_2
Itgax (CD11c)
Itgam (CD11b)
Itgae (CD103)
Xcr1
Clec9a
Cd24a
H2-Ab1
Cd9
Cd70
Itga4
H2-K1
H2-D1
Cd63
Ly75
Tnfsf9 (4-1BBL)
Cd81
Flt3
Batf3
Zbtb46
Cd1d1
Fas
Mgl2
Il1r2
Cd14
Irf2
Usp18
Stat1
Stat2
Siglech
Ly6d
Bst2
Sla2
Sell (CD62L)
Cd8a
Ptgs2
Btla
Irf4
Irf8
Stmn1
Mki67
Nfkb1
Bcl2l1
Itgax
Itgam
Itgae
Tnfsf9
Sell
+2 +1 0 -1 -2
Expression
Control mAb
αCTLA-4
αPD-1
αCTLA-4+αPD-1
Control Vax
neoAg SLP Vax

Figure S9. scRNAseq analysis of dendritic cell (DC) clusters from Y1.7LI tumor bearing mice treated with neoAg SLP vaccines or ICT.

(**A**) UMAP displaying myeloid cell sub-clustering and DC annotations (See also Figures 2A and 6A). (**B**) Heat map displaying normalized expression of select genes in each DC cluster. (**C**) Graphs depicting frequency of DCs in each cluster by condition and treatment represented as percent of live $CD45^+$ cells. (**D**) Heat map displaying normalized expression of select genes in each DC cluster by treatment condition. (**E**) Heat map displaying normalized expression of select genes in each DC cluster by treatment condition.

Figure S10

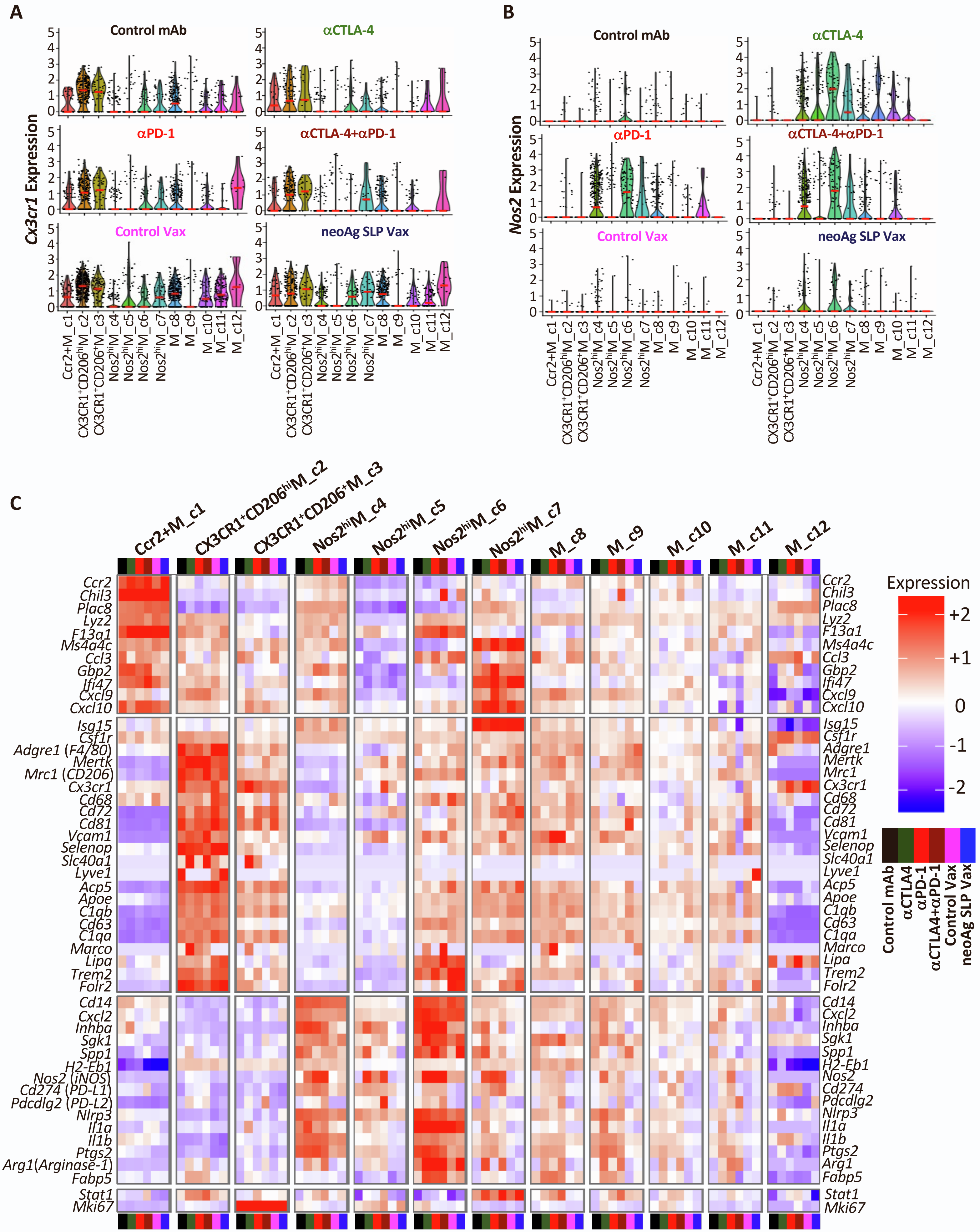
A
Control mAb
αCTLA-4
αPD-1
αCTLA-4+αPD-1
Control Vax
neoAg SLP Vax
Cx3cr1 Expression
Ccr2+M_c1
CX3CR1+CD206hiM_c2
CX3CR1+CD206+M_c3
Nos2hiM_c4
Nos2hiM_c5
Nos2hiM_c6
Nos2hiM_c7
M_c8
M_c9
M_c10
M_c11
M_c12
B
Nos2 Expression
C
Expression
+2
+1
0
-1
-2
Control mAb
αCTLA4
αPD-1
αCTLA4+αPD-1
Control Vax
neoAg SLP Vax
Ccr2
Chil3
Plac8
Lyz2
F13a1
Ms4a4c
Ccl3
Gbp2
Ifi47
Cxcl9
Cxcl10
Isg15
Csf1r
Adgre1 (F4/80)
Mertk
Mrc1 (CD206)
Cx3cr1
Cd68
Cd72
Cd81
Vcam1
Selenop
Slc40a1
Lyve1
Acp5
Apoe
C1qb
Cd63
C1qa
Marco
Lipa
Trem2
Folr2
Cd14
Cxcl2
Inhba
Sgk1
Spp1
H2-Eb1
Nos2 (iNOS)
Cd274 (PD-L1)
Pdcdlg2 (PD-L2)
Nlrp3
Il1a
Il1b
Ptgs2
Arg1(Arginase-1)
Fabp5
Stat1
Mki67

Figure S10. scRNAseq analysis of macrophage clusters from Y1.7LI tumor bearing mice treated with neoAg vaccines or ICT. Related to Figure 6.

(**A**) Violin plots denoting expression level of *Cx3cr1* transcript per cell in each monocyte/macrophage cluster by treatment condition. (**B**) Violin plots denoting expression level of *Nos2* (iNOS) transcript per cell in each monocyte/macrophage cluster by treatment condition. (**C**) Heat map displaying normalized expression of select genes in each monocyte/macrophage cluster by treatment condition.

Figure S11

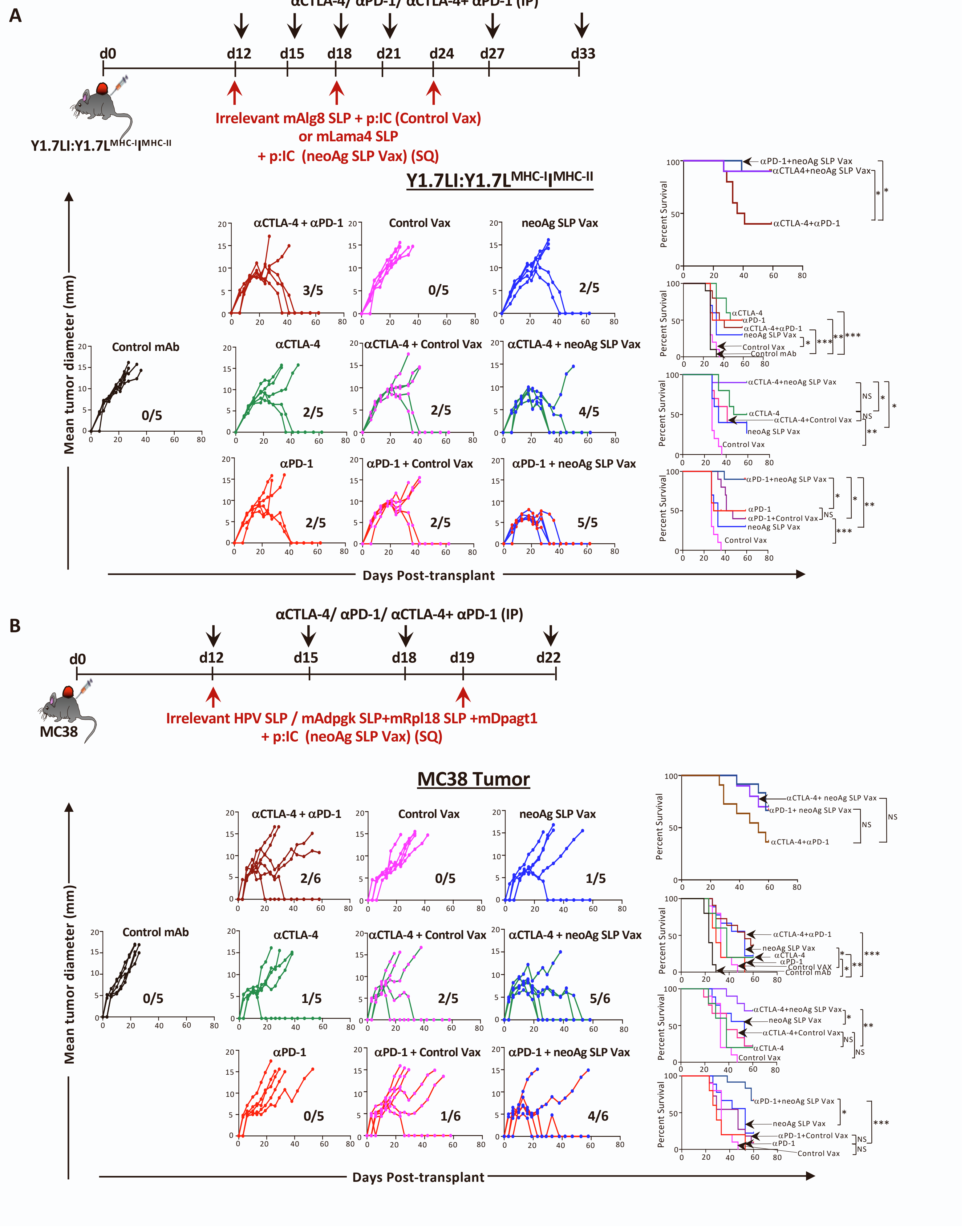
A
αCTLA-4/ αPD-1/ αCTLA-4+ αPD-1 (IP)
d0
d12
d15
d18
d21
d24
d27
d33
Y1.7LI:Y1.7L MHC-I MHC-II
Irrelevant mAlg8 SLP + p:IC (Control Vax)
or mLama4 SLP
+ p:IC (neoAg SLP Vax) (SQ)
Y1.7LI:Y1.7L MHC-I MHC-II
Mean tumor diameter (mm)
Days Post-transplant
Percent Survival
Control mAb
0/5
αCTLA-4 + αPD-1
3/5
Control Vax
0/5
neoAg SLP Vax
2/5
αCTLA-4
2/5
αCTLA-4 + Control Vax
2/5
αCTLA-4 + neoAg SLP Vax
4/5
αPD-1
2/5
αPD-1 + Control Vax
2/5
αPD-1 + neoAg SLP Vax
5/5
αPD-1+neoAg SLP Vax
αCTLA4+neoAg SLP Vax
αCTLA-4+αPD-1
αCTLA-4
αPD-1
αCTLA-4+αPD-1
neoAg SLP Vax
Control Vax
Control mAb
αCTLA-4+neoAg SLP Vax
αCTLA-4
αCTLA-4+Control Vax
neoAg SLP Vax
Control Vax
αPD-1+neoAg SLP Vax
αPD-1
αPD-1+Control Vax
neoAg SLP Vax
Control Vax
NS
B
αCTLA-4/ αPD-1/ αCTLA-4+ αPD-1 (IP)
d0
d12
d15
d18
d19
d22
MC38
Irrelevant HPV SLP / mAdpgk SLP+mRpl18 SLP +mDpagt1
+ p:IC (neoAg SLP Vax) (SQ)
MC38 Tumor
Mean tumor diameter (mm)
Days Post-transplant
Percent Survival
Control mAb
0/5
αCTLA-4 + αPD-1
2/6
Control Vax
0/5
neoAg SLP Vax
1/5
αCTLA-4
1/5
αCTLA-4 + Control Vax
2/5
αCTLA-4 + neoAg SLP Vax
5/6
αPD-1
0/5
αPD-1 + Control Vax
1/6
αPD-1 + neoAg SLP Vax
4/6
αCTLA-4+ neoAg SLP Vax
αPD-1+ neoAg SLP Vax
αCTLA-4+αPD-1
αCTLA-4+αPD-1
neoAg SLP Vax
αCTLA-4
αPD-1
Control VAX
Control mAb
αCTLA-4+neoAg SLP Vax
neoAg SLP Vax
αCTLA-4+Control Vax
αCTLA-4
Control Vax
αPD-1+neoAg SLP Vax
neoAg SLP Vax
αPD-1+Control Vax
αPD-1
Control Vax
NS

Figure S11. Anti-CTLA-4 or anti-PD-1 ICT broaden the therapeutic window for NeoAg SLP vaccines when used in combination. (**A**) Tumor growth and cumulative survival of WT C57BL/6J mice transplanted with Y1.7LI melanoma cells on d. 0 and treated beginning on d. 12 with different monotherapies: control mAb, anti-CTLA-4, anti-PD-1, irrelevant SLP + pI:C (Control Vax), or relevant mLama4 SLP + pI:C (neoAg SLP Vax); or combination therapies: anti-CTLA-4 + anti-PD-1 combination ICT, anti-CTLA-4 + control VAX, anti-CTLA-4 + neoAg SLP Vax, anti-PD-1 + control VAX, or anti-PD-1 + neoAg SLP Vax. (**B**) Tumor growth and cumulative survival of WT C57BL/6J mice transplanted with MC38 cells on d. 0 and treated beginning on d. 12 with different monotherapies: control mAb, anti-CTLA-4, anti-PD-1, irrelevant HPV SLP + pI:C (Control Vax), or relevant mAdpgk SLP + mRpl18 SLP + mDpagt1 SLP + pI:C (neoAg SLP Vax); or combination therapies: anti-CTLA-4 + anti-PD-1 combination ICT, anti-CTLA-4 + Control Vax, anti-CTLA-4 + neoAg SLP Vax, anti-PD-1 + Control Vax, or anti-PD-1 + neoAg SLP Vax. Tumor growth data in (**A**) and (**B**) are presented as individual mouse tumor growth as mean tumor diameter with fraction indicating (# of mice rejecting tumor)/(# of mice used in experiment) and are representative of three independent experiments. Cumulative survival curves in (**A**) and (**B**) include mice from three independent experiments (*$P < 0.01$, **$P < 0.05$, ***$P < 0.001$, log-rank (Mantel–Cox) test).

Figure S12

Myeloid Subsets

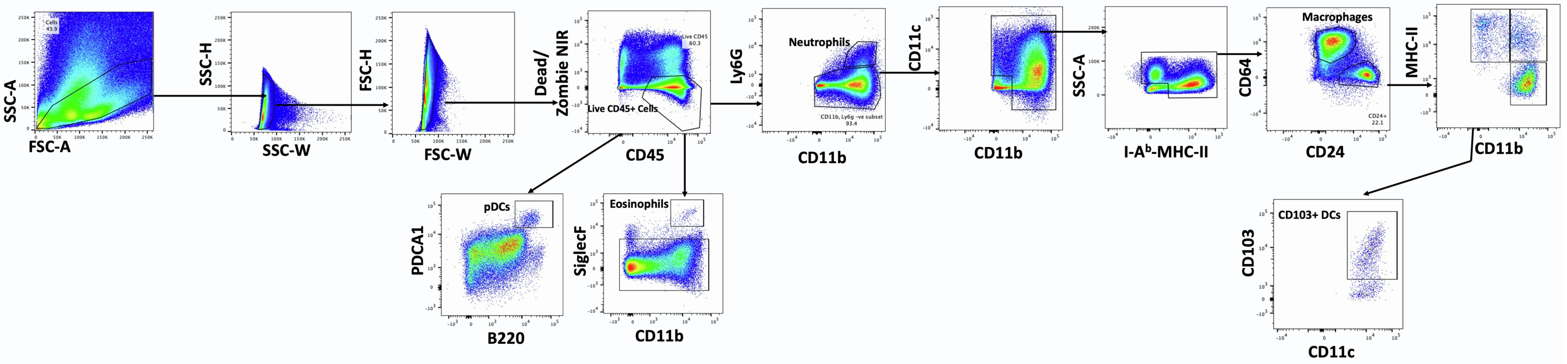

Lymphoid Subsets

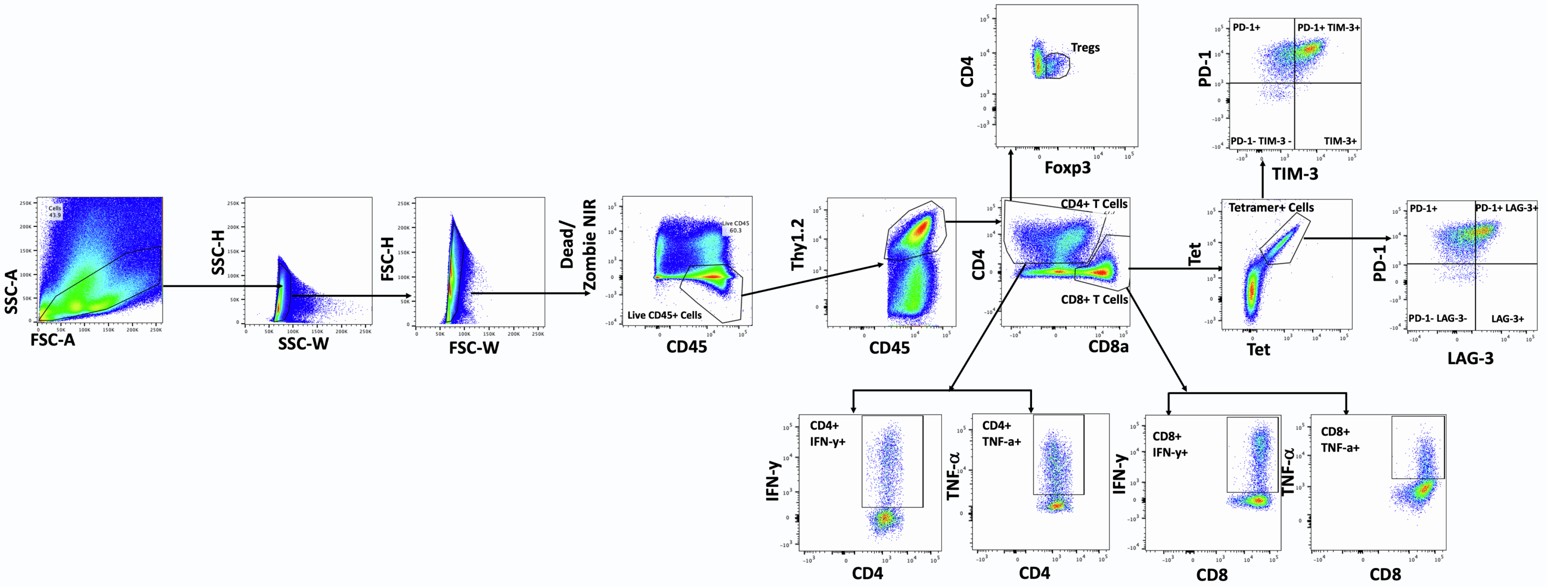

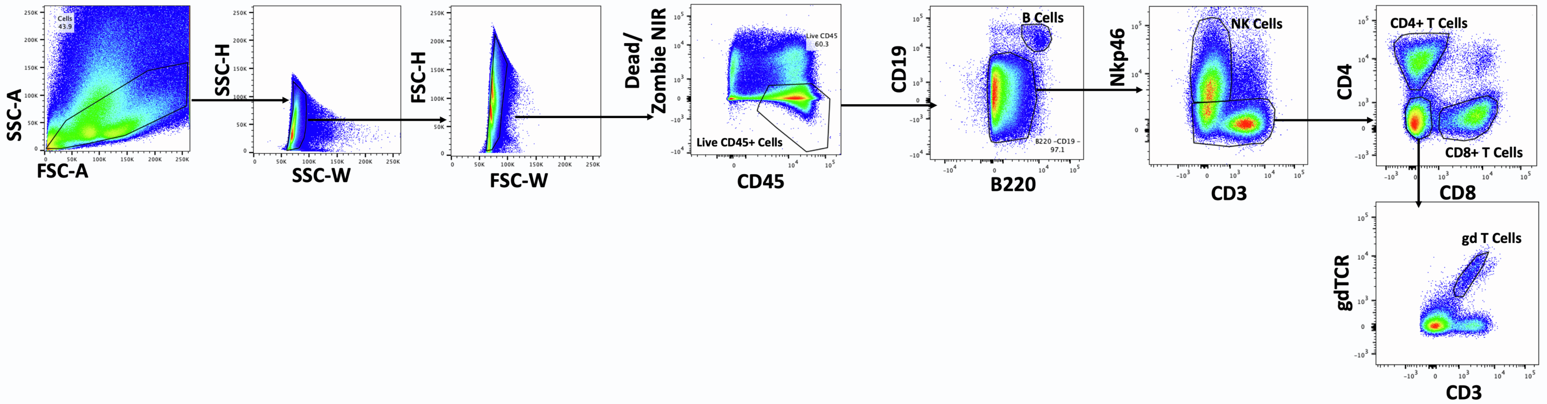

Figure S12. Gating strategy for identifying intratumoral immune cells. Flow cytometry dot plots and gating of intratumoral myeloid and lymphoid populations.
